# Subphenotyping sepsis based on organ interaction trajectory using a deep temporal graph clustering model: a retrospective cohort study

**DOI:** 10.1016/j.eclinm.2025.103691

**Published:** 2025-12-05

**Authors:** Xue Feng, Lei Sun, Jintao Zhu, Xuan Yao, Zhongheng Zhang, Qing Pan, Luping Fang, Gangmin Ning

**Affiliations:** a College of Information Engineering, Zhejiang University of Technology, Hangzhou, 310023, China; b Department of Biomedical Engineering, Zhejiang University, Hangzhou, 310027, China; c Department of Emergency Medicine, Sir Run Run Shaw Hospital, Zhejiang University School of Medicine, Hangzhou, 310016, China

**Keywords:** Sepsis, Phenotype, Deep temporal graph clustering, Organ interactions, Trajectory-based

## Abstract

**Background:**

Sepsis is a heterogeneous syndrome with varying degrees of multi-organ dysfunction. Identifying dynamic inter-organ interactions is critical for accurate sepsis subphenotyping and targeted therapy, yet remains unexplored. In this study, we aimed to quantify the dynamic trajectories of organ interactions to define sepsis phenotypes, supporting personalized treatment and clinical decision-making.

**Methods:**

We proposed a novel deep temporal graph clustering model to identify sepsis phenotypes by quantifying dynamic multi-organ interactions within 48 h post-diagnosis. The model was trained and validated on the Medical Information Mart for Intensive Care III (MIMIC-III) dataset (admissions from 2001 to 2012) and externally validated on the eICU Collaborative Research (eICU) dataset (admissions from 2014 to 2015). Its effectiveness was benchmarked against state-of-the-art clustering algorithms. Patient characteristics, multi-organ system states coupling patterns, and prognostic outcomes were compared across the identified phenotypes. Extreme gradient boosting (XGBoost) was used for early phenotype classification at 4 h post-diagnosis. To enhance clinical applicability, a user-friendly web interface was developed. Propensity score matching and weighted logistic regression were employed to evaluate the effects of the fluid management strategies on in-hospital mortality of patients with various phenotypes.

**Findings:**

A total of 10,181 and 6208 unique sepsis patients were employed as the cohorts for the model development and external validation, respectively. Three distinct phenotypes were identified and labeled as Phenotype A, B, and C, exhibiting significant differences in baseline characteristics, organ system states coupling patterns, and outcomes (P-value < 0.05). Phenotype A had the lowest mortality (5.59%) and accounted for the largest proportion of patients (46.34%). In contrast, Phenotype C represented the highest mortality (38.27%) and comprised the smallest proportion (22.78%). Phenotype A was characterized by sustained synchronous improvement across organ system states. Phenotype B showed persistent decoupling of organ system states. Phenotype C exhibited a rapid transition from early asynchrony to synchronization. The model demonstrated robust clustering performance in external validation. The simplified classifier showed high predictive performance, achieving an area under the receiver operating characteristic curve (AUROC) of 0.84 (95% CI [0.83, 0.86]) for phenotype prediction at 4 h post-diagnosis. The beneficial fluid management strategies varied across different phenotypes, highlighting the need for targeted fluid intervals.

**Interpretation:**

This study characterizes sepsis phenotypes using organ interaction trajectories and identifies three heterogeneous patterns of disease progression. These patterns offer new insights into the underlying pathophysiological mechanisms of sepsis, which can support the design of clinical trials on disease progression and guide the optimal allocation of intensive care resources.

**Funding:**

This study was funded by the 10.13039/501100001809National Nature Science Foundation of China (No. 32371372) and the 10.13039/501100012166National Key Research and Development Program of China (No. 2022YFC2009503).

**Research in context**

**Evidence before this study**

The complex interaction among multiple organ systems in sepsis exhibits significant heterogeneity and dynamic variability, posing challenges for precise therapeutic interventions and prognostic management. Recently, clustering-based approaches have been proposed to identify distinct sepsis phenotypes, aiming to elucidate the underlying heterogeneity. We searched in PubMed, Google Scholar, and preprint platforms (medRxiv, bioRxiv, and arXiv) for articles published until May 2024, using the search terms [“sepsis”] AND [“subphenotype” OR “phenotype” OR “subclass” OR “subtype”] AND [“organ dysfunction” OR “multi-organ failure” OR “organ crosstalk” OR “organ interaction”] AND [“machine learning” OR “artificial intelligence” OR “clustering”]. We found that existing studies have employed various traditional clustering methods to define sepsis phenotypes based on dynamic physiological trajectories. However, critical limitations remain: existing approaches fail to adequately capture the complex and dynamic interaction among organ dysfunctions, thereby hindering the development of prognosis-driven and precision-guided fluid management strategies.

**Added value of this study**

This study proposed a novel deep temporal graph clustering model to quantitatively analyze organ interaction trajectories in sepsis. Using this model, we identified three clinically distinct phenotypes in a cohort of 10,181 patients from the Medical Information Mart for Intensive Care III (MIMIC-III) dataset and externally validated these findings in an independent cohort of 6208 patients from eICU Collaborative Research (eICU). Compared to traditional phenotype clustering methods, our model achieves two key advancements: (1) introduced the first clustering approach based on organ interaction trajectories, enabling quantitative characterization of dynamic organ network interactions, (2) examined organ system states coupling patterns across phenotypes. The identified phenotypes exhibit significant differences in long-term prognostic outcomes, organ system states coupling patterns, and clinical manifestations. Notably, the identified patterns — sustained synchronous improvement (Phenotype A), persistent decoupling (Phenotype B), and a rapid transition from asynchrony to synchronization (Phenotype C) — offer new insights into the underlying pathophysiology of the disease. The progression of these phenotypes cannot be fully explained by conventional severity scores, such as the Sequential Organ Failure Assessment (SOFA). Moreover, the beneficial fluid management strategies differ across phenotypes, providing valuable guidance for clinical decision-making and the optimization of ICU resource allocation. To facilitate clinical application, we developed a phenotype-based extreme gradient boosting (XGBoost) classifier, providing a user-friendly tool for clinicians to enhance precision-guided decision-making in sepsis management.

**Implications of all the available evidence**

The proposed novel model demonstrated its effectiveness in characterizing sepsis organ interaction trajectories, enhancing the understanding of the heterogeneous progression of sepsis. Our study provided a valuable tool for sepsis patient subphenotyping in future clinical trials and may optimize the allocation of intensive care resources.

## Introduction

Sepsis is life-threatening organ dysfunction caused by a dysregulated host response to infection.^1^^,^^2^ It is associated with high disability and mortality, particularly in intensive care unit (ICU) patients, where it is a leading cause of death.^3^ Globally, more than 29.5% of ICU patients experience sepsis,^4^ which accounts for 30%–50% of all ICU deaths.^5^ Timely and precise intervention is critical for reducing sepsis-related complications and mortality.^6^^,^^7^ However, the heterogeneity among sepsis patients presents significant challenges to effective treatment management.^8^, ^9^, ^10^

Accurate and rational classification of sepsis patients is a critical prerequisite for personalized precision medicine.^11^ Current studies primarily employ clustering algorithms to identify distinct sepsis endotypes and phenotypes,^9^^,^^12^, ^13^, ^14^, ^15^, ^16^ aiming to inform stratified treatment strategies. Genomic-based endotype classification is time-consuming and costly, while the rapid progression of sepsis necessitates timely and adaptive clinical decision-making. Consequently, many studies have focused on refining sepsis phenotypes using readily accessible clinical monitoring data.^9^^,^^10^^,^^15^

Given the highly dynamic nature of sepsis progression, Bhavani et al. categorized patients into four phenotypes based on body temperature trajectories^17^^,^^18^ and other vital signs,^19^ revealing significant clinical heterogeneity among subgroups. Xu et al. applied dynamic time warping and hierarchical clustering to delineate four sepsis subtypes from Sequential Organ Failure Assessment (SOFA) score trajectories: rapid and delayed deterioration, and rapid and delayed improvement phenotypes.^20^ It suggests that the temporal evolution of organ dysfunction is closely associated with sepsis phenotypic heterogeneity. Previous studies showed that organs exhibited complex crosstalk, where damage to one organ could worsen injury in others, increasing the complexity of sepsis management.^21^^,^^22^ Such dynamic multi-organ interactions markedly complicated phenotype classification based on clinical presentation. Existing approaches typically treat SOFA scores or their organ-specific subscore trajectories as independent time series,^23^ overlooking the spatiotemporal interdependencies among organ systems and thereby limiting insights into multi-organ interaction mechanisms. It constrains the precision of organ state characterization and hinders accurate phenotype identification, ultimately impeding individualized treatment decisions. Systematic modeling of dynamic inter-organ dysfunction trajectories is crucial for understanding the natural course of sepsis and identifying subtypes responsive to targeted therapies.

Moreover, existing studies primarily employ conventional clustering algorithms for sepsis phenotyping, which are limited in capturing the complex, high-dimensional nature of sepsis and fall short in characterizing its underlying heterogeneity.^24^ Deep clustering methods have recently emerged as promising alternatives, offering enhanced phenotypic stratification through superior nonlinear feature representation.^25^ Nevertheless, most current deep clustering models lack temporal awareness and fail to model dynamic feature interactions, limiting their ability to capture the evolving graph structures inherent in sepsis trajectories. In addition, it struggles to identify patient subgroups with similar clinical presentations and prognostic outcomes, impeding the development of truly homogeneous phenotype clusters.^26^^,^^27^ Therefore, it is essential to develop a deep clustering algorithm capable of capturing interactions among multi-organ trajectories, thereby exploring new sepsis phenotypes and guiding more precise treatment.

**Fig. 1:**
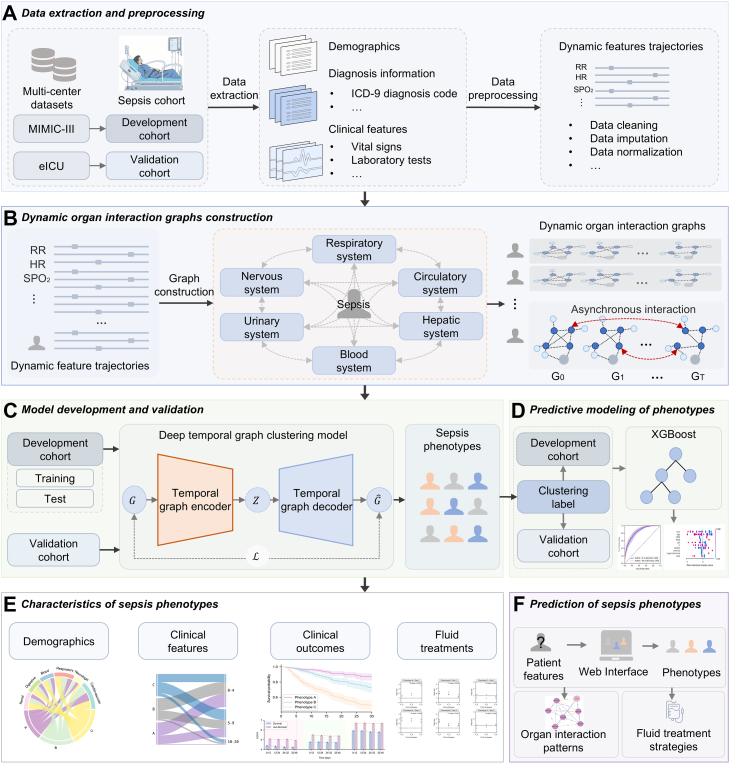
Schematic of the study workflow. A. Data extraction and preprocessing. Clinical data were collected from two publicly available databases: Medical Information Mart for Intensive Care III (development cohort) and eICU (external validation cohort). Temporal features were preprocessed via data cleaning, imputation, and normalization. B. Dynamic organ interaction graph construction. Time-resolved organ interaction graphs were constructed to represent key physiological systems. For each patient, a sequence of graphs (G_1_ to G_T_) captured asynchronous inter-organ interactions over time. C. Model development and validation. A deep temporal graph clustering model was developed using the graph sequences as input. Low-dimensional embeddings Z were learned and clustered to identify distinct sepsis phenotypes. D. Predictive modeling of phenotypes. The phenotypes served as labels to train an extreme gradient boosting (XGBoost) classifier. Performance was evaluated using receiver operating characteristic curves, and feature importance was analyzed. E. Characteristics of sepsis phenotypes. Identified phenotypes were compared in terms of demographics, clinical features, outcomes, and fluid management strategies. F. Prediction of sepsis phenotypes. A web-based tool was implemented to predict phenotypes from early patient data, providing organ interaction patterns and phenotype-specific fluid therapy recommendations.

In this study, we proposed a novel deep clustering algorithm named outcome guided deep temporal graph clustering network (ODTGCNet). It was designed to identify and verify sepsis phenotypes using multi-organ interaction trajectories from retrospective electronic medical records (EHRs). The heterogeneous correlations between clinical manifestations, multi-organ system states coupling patterns, prognostic outcomes, and fluid therapy strategies among different sepsis phenotypes were explored. To facilitate clinical application, a simple classifier was constructed based on easily accessible clinical features to identify sepsis phenotypes early, and the importance of different features for phenotype definition was explored. Overall, we aimed to reveal different multi-organ dynamic interaction patterns in sepsis and highlight promising clinical application prospects in sepsis management. The overall study design is depicted in Fig. 1.

## Methods

### Datasets and study cohorts

EHR data from two publicly available critical care databases were used for model development and validation. The development cohort was derived from the Medical Information Mart for Intensive Care III (MIMIC-III) database, comprising ICU admissions from 2001 to 2012.^28^ The external validation cohort was obtained from the multicenter eICU Collaborative Research (eICU) database, which includes admissions from 2014 and 2015.^29^ A detailed description of the two databases is provided in Supplementary Methods 1.1.

The selection process for the sepsis cohort is illustrated in Supplementary Fig. S1. The sepsis cohort was determined based on the Sepsis-3.0 diagnostic criteria.^1^ Sepsis was defined as a suspected infection (inferred from antibiotic prescriptions and fluid sampling for microbial culture) accompanied by a SOFA score ≥2. The determination of sepsis onset adhered to specific temporal diagnostic criteria: if microbial sampling was performed first, antibiotics must be administered within 72 h; if antibiotics were initiated first, microbial sampling must occur within 24 h. The onset time was defined by the earliest occurrence of these relevant events. Eligible patients were those aged ≥16 years, diagnosed with sepsis within 24 h of ICU admission, and with a hospital stay of at least 48 h after sepsis onset. For patients with multiple ICU admissions, only the first admission was analyzed. Patients with missing in-hospital survival status or documented withdrawal or failure of treatment were excluded.

### Clinical features selection and data preprocessing

Based on existing research and expert recommendations,^30^^,^^31^ six physiological systems were selected for subphenotyping, each represented by time-varying clinical features of organ function. These features were sampled every 4 h during the first 48 h following sepsis diagnosis, yielding 12-time steps. When multiple measurements occurred within a single interval, the average was used. To ensure model reliability, features with a missing rate exceeding 40% across the cohort were excluded, as were patients with more than 40% missing features. Additionally, patients lacking essential bedside monitoring data (e.g., respiratory rate, heart rate) within the first 24 h post-diagnosis were removed. The final study cohort included 10,181 patients with 33 features from the MIMIC-III database and 6208 patients from the eICU database. Baseline demographics, comorbidities, vital signs, clinical scores, and outcomes of study cohorts in two databases are summarized in Supplementary Table S1. The MIMIC-III dataset was randomly divided into training, validation, and test sets in a 6:2:2 ratio, with the validation set used to optimize hyperparameter selection. The entire cohort from the eICU dataset served as an independent external validation set.

Time-series data were imputed using forward and backward filling. For features entirely missing across a patient's sampling window, the cohort mean was used to avoid complete data loss. Outliers were identified and removed using the interquartile range (IQR) method to ensure data consistency. Sensitivity analyses regarding missing data imputation and outlier handling are detailed in Supplementary Methods 1.1. All features were then normalized.

### Sepsis organ interaction graph design

A sepsis organ interaction graph G=(V,E) was constructed based on the physiological systems and clinical characteristics encompassed by the SOFA score, as depicted in Supplementary Fig. S2. Nodes V represent entities, while edges E denote the relationship between nodes. The graph's nodal attributes were temporally evolving, effectively mirroring the patient's fluctuating physiological state. It comprises 13 nodes, including one patient node, six system nodes, and six clinical feature nodes. The patient node was defined by demographic features, including age, gender, and weight. Physiological system nodes represented six organ systems — circulatory, urinary, hematologic, hepatic, respiratory, and nervous — with each node characterized by a vector of associated disease diagnoses. Physiological characteristic nodes represented corresponding laboratory tests and vital signs. All physiological system nodes were fully connected to the patient node, while physiological characteristic nodes were connected only to their respective system nodes. Node features for each system were defined based on disease presence, including only diagnoses that occurred more than 1000 times in the study cohort. A binary encoding scheme was applied, assigning 1 for disease presence and 0 for absence. Details of the nodes and their features are provided in Supplementary Table S2.

### Subphenotyping model development and validation

**Fig. 2:**
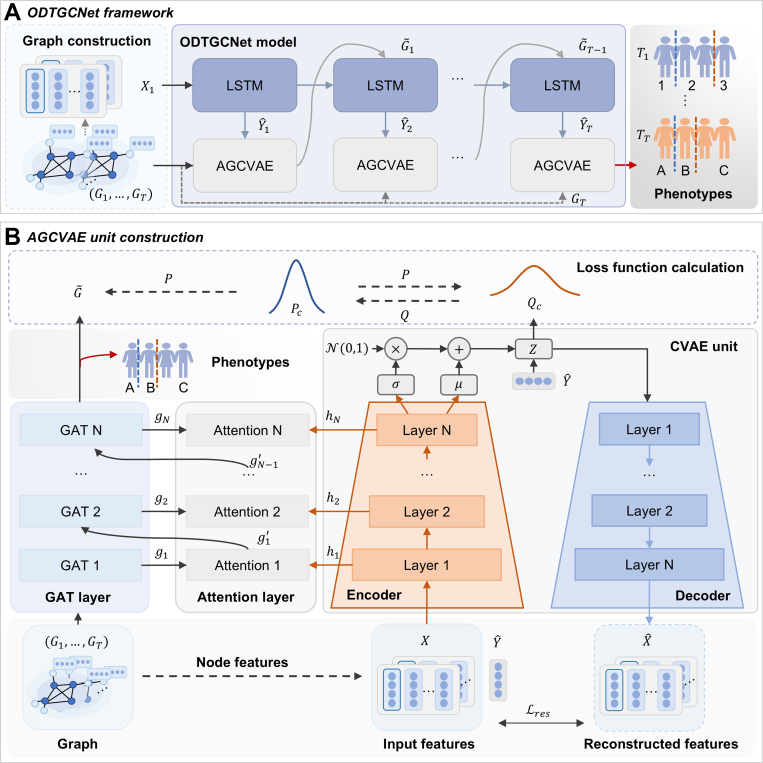
The overall structure for the proposed ODTGCNet model. A. ODGCNet framework. The ODTGCNet model is capable of providing dynamic risk grading at each timestep and the final phenotypes. B. AGCVAE unit construction.

We proposed a deep temporal graph clustering model, ODTGCNet, to identify latent patterns within the sepsis dynamic organ interaction graph sequence. As illustrated in Fig. 2, the model takes a sequence of sepsis patient-specific graphs $\left\{G_{1},G_{2},...,G_{T}\right\}$ as input, with a binary prognostic label Y∈{0,1}, where 1 indicates death and 0 indicates survival. The model outputs K cluster centroids, denoted as C={C(1),C(2),…,C(K)}. At each time step t, it produces a stratified risk prediction represented as $C_{t}$.

The overall model architecture is as follows: a long short-term memory (LSTM) module is first used to capture temporal dependencies and generate an outcome prediction at each time step. It serves as a conditional input to the conditional variational autoencoder (CVAE) module at the next time step, guiding representation learning. At the initial time step, the LSTM takes the node feature sequence $X_{1}$ from $G_{1}$ as input. In subsequent steps, it receives the output from the preceding CVAE module. An attention-based graph conditional variational autoencoder (AGCVAE) module is constructed to extract deep representations of patient features at each time point. It integrates a graph attention network (GAT) to perform feature aggregation and information propagation over the organ graph, where learnable edge weights are assigned to neighboring nodes via an attention mechanism. In addition, a CVAE is employed to model the latent distribution of node features, enabling adaptive fusion of structural and semantic representations through attention-based integration. The AGCVAE takes as input the graph sequence and the predicted outcomes from the LSTM module $\left\{{\hat{Y}}_{1},{\hat{Y}}_{2},\ldots ,{\hat{Y}}_{T}\right\}$. The CVAE generates latent representations $\left\{h_{1},h_{2},...,h_{N}\right\}$ conditioned on node features and predicted labels $\hat{Y}$, while the GAT processes the graph structure G to produce structural embeddings $\left\{g_{1},g_{2},...,g_{N}\right\}$. It fuses the two representations through an adaptive attention mechanism to obtain joint embeddings $\{{g_{1}}^{\prime },{g_{2}}^{\prime },...,g_{N}\prime \}$, which are then fed into the next GAT layer for further modeling. More details construction of the proposed model and network architecture are presented in Supplementary Methods 1.2 and Supplementary Table S3.

To achieve unified optimization of clustering and representation learning, a target-oriented self-supervised loss function was introduced to direct the parameter updates of the entire model. Referring to the previous study, a centroid-based probability distribution Q is defined. By minimizing the kullback-leibler (KL) divergence between this distribution and an auxiliary distribution P, both clustering quality and feature representation performance are enhanced. In the unsupervised learning task, the clustering objective function of CVAE is formulated as follows.

$$\left\{ \begin{array}{c} L_{C}=KL\left(P∥Q\right)=\sum_{i}\sum_{u}p_{iu}\;\log\frac{p_{iu}}{q_{iu}}\\ q_{ij}=\frac{{\left(1\;+\;{\left\|z_{i}\;-\;\mu_{j}\right\|}^{2}\right)}^{-1}}{\sum_{j}{\left(1\;+\;{\left\|z_{i}\;-\;\mu_{j}\right\|}^{2}\right)}^{-1}}\\ p_{ij}=\frac{\frac{q_{ij}^{2}}{\sum_{i}q_{ij}}}{\sum_{j}\left(\frac{q_{ij}^{2}}{\sum_{i}q_{ij}}\right)} \end{array}\right.$$

where $q_{ij}$ represents the similarity between the embedded vector $z_{i}$ and the cluster center $\mu_{j}$, which is quantified using the student's t-distribution. The $p_{ij}$ denotes the auxiliary target distribution. The initial cluster centers $\mu_{j}$ are obtained via K-Means. Since the auxiliary distribution P is defined based on the distribution Q, minimizing $L_{C}$ constitutes a form of self-supervised training.

For the GAT model, the clustering objective function is defined as $L_{G}$. A simple and flexible inner-product decoder is employed to predict the connectivity between nodes, where $\hat{A}$ represents the reconstructed adjacency matrix. To measure the reconstruction error between A and $\hat{A}$, a cross-entropy loss function $L_{AR}$ is utilized.

$$\begin{array}{c} L_{G}=KL\left(P∥{{\tilde{G}}^{N}}^{\prime }\right)=\sum_{i}\sum_{u}p_{iu}\;\log\frac{p_{iu}}{{{\tilde{G}}^{N}}_{\mathrm{iu}}^{\prime }} \end{array}$$

$$\begin{array}{c} \left\{ \begin{array}{l} {\hat{A}}_{ij}=sigmoid\left({z_{i}}^{T}z_{j}\right)\\ L_{AR}=\sum_{l=1}^{L}loss\left(A_{ij},{\hat{A}}_{ij}\right) \end{array}\right. \end{array}$$

Furthermore, a cross-entropy loss function is employed to quantify the discrepancy between the model's predicted outcomes and the actual patient outcome labels, guiding the model to identify prognosis-sensitive clustering phenotypes.

$$\begin{array}{c} L_{S}=-\frac{1}{M}\sum_{1}^{M}y_{t}\;\log\;{\hat{y}}_{t}\;+\;\left(1\;-\;y_{t}\right)\log\left(1\;-\;{\hat{y}}_{t}\right) \end{array}$$

Considering the overall objective of the ODTGCNet model, a composite loss function is formulated, where α, β, γ, and σ are weighting coefficients that balance the relative contributions of different components, ensuring coordinated optimization of various tasks during model training.

$$\begin{array}{c} L=L_{G}+\alpha L_{C}+\beta L_{AR}+\gamma L_{CVAE}+\sigma L_{S} \end{array}$$

The model was trained end-to-end, culminating in the output of temporal risk stratification and clustering results. To enable dynamic labeling of each patient's risk status, phenotype classification was performed at the 48-h mark, while risk stratification was generated at fixed 4-h intervals throughout the observation window. The output of the model is formulated as:

$$C={\mathrm{argmax}}_{j}q_{ij}$$

where $q_{ij}$ denotes the similarity between the embedded point $z_{i}$ and the cluster center $\mu_{j}$. The specific architecture and implementation details of the proposed model are provided in Supplementary Methods 1.2.

The optimal number of clusters k is determined using the Elbow Method.^32^ By plotting the relationship between the sum of squared errors (SSE) and varying values of k, an elbow-shaped inflection point is identified. The value of k at this inflection point indicates the appropriate number of clusters within the data. The detailed calculation procedure for determining k is provided in the Supplementary Methods 1.3. As illustrated in Supplementary Fig. S3, the SSE decreased progressively with an increasing number of clusters k. Once k reached the true number of clusters, further increases yielded diminishing returns, with the rate of SSE reduction markedly slowing and eventually plateauing. Consequently, the optimal number of clusters at various time points following sepsis diagnosis was identified as 3.

To evaluate the effectiveness of the proposed method, state-of-the-art (SOTA) models, including the deep embedding clustering algorithm^33^^,^^34^ and the deep graph clustering algorithm,^35^^,^^36^ were selected as baselines for comparison. In addition, a series of ablation experiments was conducted to systematically evaluate the contribution and effectiveness of each component of the ODTGCNet model (detailed in Supplementary Methods 1.4). All models were trained on the MIMIC-III dataset and externally validated on the eICU database to assess generalizability and robustness. The clustering performance of the model was evaluated using the silhouette coefficient (SC)^37^ and the Davies-Bouldin index (DBI)^38^ (Supplementary Methods 1.3).

### Subphenotype classifier construction and web interface construction

To facilitate clinical application, an efficient and lightweight phenotype classifier was developed using extreme gradient boosting (XGBoost), based on patient features at 4 h combined with clustering results from the ODGCNet model as labels. The development cohort served as the training set, with internal validation performed using 5-fold cross-validation. The validation cohort was used as an external testing set. The model was evaluated using the area under the receiver operating characteristic curve (AUROC) and the area under the precision–recall curve (AUPRC). Shapley Additive exPlanations (SHAP) were used to further interpret feature contributions. Web interface implementation was detailed in Supplementary Methods 1.5.

### Characterizing multi-organ states coupling patterns in sepsis phenotypes

To investigate the dynamic multi-organ states coupling patterns across different sepsis phenotypes, phenotype-specific organ system graphs were constructed. The proposed model represented patient's organ functional state as a deep embedding vector, and cosine similarity was applied to quantify the similarity between organ state embeddings, defined as the organ state coupling strength (detailed in Supplementary Methods 1.2.1). A higher coupling strength indicated more similar and synchronous functional states, whereas a lower coupling strength indicated dissimilar and asynchronous states. Based on patients' clinical outcomes, organ states coupling strength matrices at 4, 12, and 48 h were generated and compared across phenotypes, with the distribution of synchronous inter-organ coupling visualized using heatmaps. To capture the temporal evolution of coupling strength, a single organ system was designated as the target node, and heatmaps were plotted to show its coupling strength with other systems over time. To characterize asynchronous coupling patterns, the target organ's state at 48 h was fixed, and its coupling strength with other organ systems at earlier time points was tracked. These analyses revealed the evolving similarity among key organ systems, including the heart-kidney and lung–coagulation axes, and highlighted phenotype-specific differences in multi-organ states coupling throughout the course of illness.

### Investigating prognostic value of phenotypes for clinical outcomes

To assess the prognostic value of the identified patient phenotypes, in-hospital mortality was used as the endpoint, and separate logistic regression models were constructed using phenotypes alone, SOFA (including baseline and 48-h change) alone, or Acute Physiology and Chronic Health Evaluation II (APACHE II) scores alone. Model performance was evaluated using AUROC, recall, precision, F1 score, and specificity. To further determine the independent prognostic value of phenotype classification, multivariable logistic regression was performed, adjusting for key clinical confounders, including age, sex, baseline SOFA, Elixhauser Comorbidity Index (ECI), respiratory rate, and mean arterial pressure, with odds ratios (ORs) and 95% confidence intervals (CIs) calculated for each predictor. Additionally, we assessed the independent predictive ability of phenotypes for mortality while controlling for APACHE II scores. To evaluate the independent prognostic value of phenotype classification within the same level of organ dysfunction, we compared in-hospital mortality across phenotypes within each SOFA score stratum (2–6, 7–10, ≥11).^39^

### Heterogeneous treatment effect of intravenous fluid strategies

To investigate the heterogeneity in intravenous fluid responsiveness among different sepsis phenotypes, we included patients with a treatment duration exceeding 24 h. For each patient, total fluid volume administered within 0–12 h and 12–24 h after diagnosis was calculated,^40^ and in-hospital survival outcomes were recorded (details on fluid types and filtering criteria are provided in Supplementary Methods 1.6). The total fluid volume within 0–12 h and 12–24 h was categorized into three levels, low, moderate, and high volume (Fluid Strategies 0–2: 0–30 mL/kg, 30–50 mL/kg, and > 50 mL/kg), respectively, to assess the potential impact of fluid therapy on prognosis. To control for confounding, a multinomial logistic regression model incorporating baseline patient characteristics was used to estimate propensity scores (PS), which were then used to compute stabilized inverse probability weights (SIPW). A weighted multinomial logistic regression model was constructed with in-hospital mortality as the outcome variable, and fluid strategy, phenotype, and their interaction term as primary predictors. Covariates included age, sex, SOFA score, arterial lactate, Glasgow Coma Scale (GCS) score, heart rate, systolic and diastolic blood pressure, respiratory rate, oxygen saturation, ICU type, maximum vasopressor dose, hemoglobin, fraction of inspired oxygen (FiO_2_), and body temperature. To evaluate whether the effect of fluid strategy differed significantly across phenotypes, we performed a likelihood ratio test comparing the interaction model with a reduced model excluding the interaction term. Using Fluid Strategy 0 (low volume) as the reference group, ORs and 95% CIs were derived to quantify the association between fluid strategy and mortality. Additionally, to assess whether ICU type modified the effect of fluid strategy on outcomes, we constructed a logistic regression model including an interaction term between ICU type and fluid strategy, and conducted a likelihood ratio test accordingly (see Supplementary Methods 1.7 for details).

### Statistical analysis

Statistical analyses were performed using SPSS 26.0 software. Prior to group comparisons, the normality of continuous variables was assessed using the Shapiro–Wilk test. For variables that met the assumption of normal distribution, parametric tests (Student's t-test or Analysis of Variance (ANOVA)) were applied. For non-normally distributed variables, non-parametric tests (Mann–Whitney U test or Kruskal–Wallis H test) were used to evaluate differences in central tendency across independent groups. A P-value < 0.05 for two sides was considered statistical significance. Continuous variables were summarized as medians and interquartile ranges. To assess survival risk within 28 days after sepsis diagnosis, Kaplan–Meier (KM) survival analysis was employed. The KM method is a widely used univariate survival analysis technique, which visually presents the changes in survival data over time through survival curves. In KM curves, the *x*-axis represents survival time, the *y*-axis indicates survival probability, the starting point corresponds to the initiation of the follow-up, and the downward slope reflects patient survival risk.

### Ethics committee approval

All patient data were retrospectively collected from the electronic medical record systems, third-party public databases. The MIMIC-III and eICU databases used in this study are publicly available and de-identified. Accordingly, ethics approval and informed consent were not required for this retrospective secondary-data analysis.

### Role of the funding source

We declare that the funder has no role in study design, data collection, data analysis, data interpretation, writing of the report, and decision to submit the paper for publication.

## Results

### Patients and study cohorts

A total of 2432 patients were included in development cohort, with a median age of 65.41 years (standard derivation (SD): 16.54), and 1404 (57.7%) are male. The overall in-hospital mortality was 15.8%, and the mean ICU length of stay was 6.88 days (SD: 8.12). The average SOFA score obtained within the first 4 h of ICU admission was 5.00 (SD: 2.71). The validation cohort from the eICU dataset (n = 6208) demonstrated a demographic distribution similar to that of the development cohort. Compared to the development cohort, the validation cohort exhibited a slightly higher in-hospital mortality (16.3%), a shorter ICU length of stay (5.05 days (SD: 5.54)), and a marginally higher initial SOFA score (5.27 (SD: 2.19)).

### Heterogeneous characteristics across derivation of subphenotypes

**Fig. 3:**
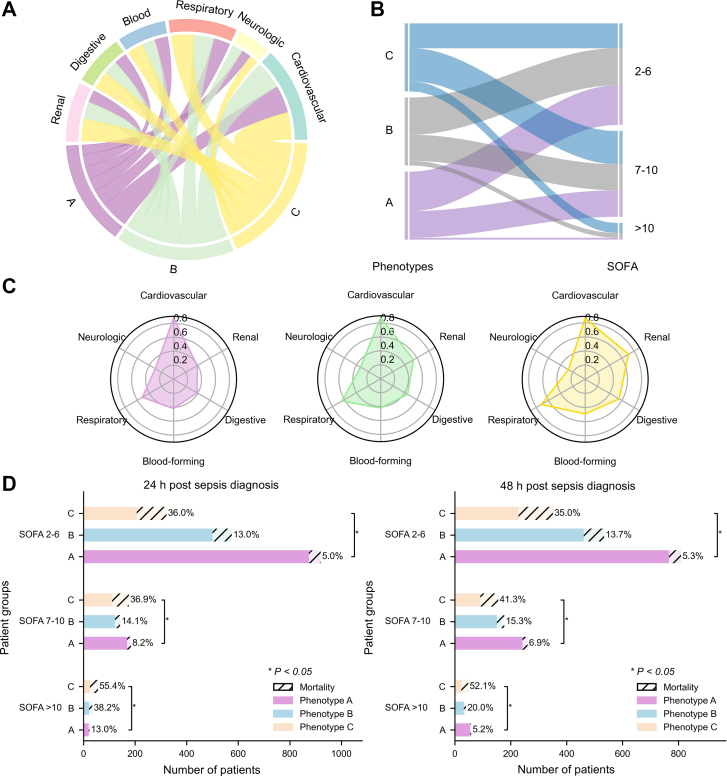
Comparison of organ dysfunction across sepsis phenotypes in the medical information mart for intensive care III test cohort. A. Chord diagram of comorbidities across phenotypes. This diagram visualizes the distribution of organ system dysfunctions across different phenotypes. The affected organ systems include the renal, gastrointestinal, hematologic and hematopoietic, respiratory, neurological, and circulatory systems. The bandwidth represents the proportion of patients with a specific organ dysfunction within each phenotype. B. Alluvial plot of Sequential Organ Failure Assessment (SOFA) score distribution among phenotypes. The left column represents sepsis phenotypes, while the right column categorizes SOFA scores into three levels. Each phenotype is color-coded, and the alluvial plot illustrates the relationship between different sepsis phenotypes and SOFA score distributions. C. Radar chart of comorbidities across phenotypes. This radar chart depicts the prevalence of comorbidities among different phenotypes, using the same organ systems as shown in A. D. Phenotype distribution and mortality across SOFA strata at 24 and 48 h after sepsis diagnosis. The *x*-axis represents the number of patients; the *y*-axis denotes phenotype groups. ∗P-value < 0.05 indicates a significant difference.

The proposed deep temporal graph clustering model effectively identified and characterized three distinct sepsis phenotypes, designated Phenotypes A, B, and C. The clinical characteristics of each phenotype are presented in Table 1 and Fig. 3. Statistical analyses confirmed significant differences among the phenotypes (P-value < 0.05). Phenotype A comprised the largest proportion of patients (n = 1127 (46.34%)), whereas Phenotype C represented the smallest (n = 554 (22.78%)). Patients in Phenotype A were the youngest (62.28 years (SD: 16.69)) and exhibited the lowest disease severity, as indicated by the lowest average SOFA score of 4.48 (SD: 2.24). In contrast, Phenotype C included the oldest patients (70.63 years (SD: 15.42)) and demonstrated the highest SOFA score of 6.16 (SD: 3.28). It underscores substantial variation in age and disease severity across the identified sepsis phenotypes.

**Table 1:** Characteristics of sepsis phenotypes in the medical information mart for intensive care III database.

Characteristics	All patients	Phenotypes			P-value
		A	B	C	
**Number of patients**	2432	1127	751	554	
**Demographic information**					
**Gender (%)**					0.044^a^
Male	1404 (57.7)	681 (60.4)	415 (55.3)	308 (55.6)	
Female	1028 (42.3)	446 (39.6)	336 (44.7)	246 (44.4)	
**Race (%)**					0.359
Asian	37 (1.5)	18 (1.6)	9 (1.2)	10 (1.8)	
Black	187 (7.7)	83 (7.4)	59 (7.9)	45 (8.1)	
Hispanic	79 (3.3)	49 (4.3)	21 (2.8)	9 (1.6)	
White	1759 (72.3)	813 (72.1)	557 (74.2)	389 (70.2)	
Unknown	370 (15.2)	164 (14.6)	105 (14.0)	101 (18.2)	
Age, years (mean (std))	65.41 (16.54)	62.28 (16.69)	66.26 (16.04)	70.63 (15.42)	0.000^a^
Weight, kg (mean (std))	83.09 (22.79)	86.24 (23.42)	82.27 (23.18)	77.81 (19.75)	0.000^a^
**ICU types (%)**					0.000^a^
CCU	295 (12.1)	130 (11.5)	84 (11.2)	81 (14.6)	
CSRU	555 (22.8)	380 (33.7)	136 (18.1)	39 (7.0)	
MICU	907 (37.3)	303 (26.9)	312 (41.5)	292 (52.7)	
SICU	335 (13.8)	140 (12.4)	114 (15.2)	81 (14.6)	
TSICU	340 (14.0)	174 (15.4)	105 (14.0)	61 (11.0)	
**Comorbidities (%)**					
Cardiovascular diseases	2121 (87.2)	984 (87.3)	659 (87.7)	478 (86.3)	0.728
Neurological diseases	744 (30.6)	341 (30.3)	251 (33.4)	152 (27.4)	0.064
Respiratory diseases	1463 (60.2)	584 (51.8)	482 (64.2)	397 (71.7)	0.000^a^
Hematologic diseases	1061 (43.6)	473 (42.0)	316 (42.1)	272 (49.1)	0.013^a^
Gastrointestinal diseases	1083 (44.5)	439 (39.0)	336 (44.7)	308 (55.6)	0.000^a^
Renal diseases	1245 (51.2)	442 (39.2)	410 (54.6)	393 (70.9)	0.000^a^
**Clinical scores (mean (std))**					
SOFA score	5.00 (2.71)	4.48 (2.24)	4.91 (2.63)	6.16 (3.28)	0.000^a^
GCS score	14.42 (1.46)	14.53 (1.28)	14.46 (1.27)	14.13 (1.92)	0.000^a^
ECI score	0.16 (1.81)	−0.16 (1.53)	0.22 (1.82)	0.71 (2.16)	0.000^a^
**Vital signs**					
Respiratory rate, breaths/min	19.11 (4.51)	18.17 (4.04)	19.56 (4.75)	20.41 (4.66)	0.000^a^
Heart rate, beats/min	88.53 (16.29)	86.84 (15.43)	89.33 (16.88)	90.87 (16.82)	0.000^a^
Systolic blood pressure, mmHg	117.36 (16.27)	117.41 (15.69)	119.09 (16.83)	114.88 (16.38)	0.000^a^
Diastolic blood pressure, mmHg	59.98 (10.41)	60.70 (10.21)	60.26 (10.49)	58.13 (10.51)	0.000^a^
Mean arterial pressure, mmHg	77.66 (11.06)	78.39 (10.73)	78.26 (11.36)	75.39 (11.02)	0.000^a^
Oxygen saturation, %	97.56 (2.55)	97.95 (1.93)	97.52 (2.25)	96.80 (3.64)	0.000^a^
Body temperature, °C	36.88 (0.78)	36.89 (0.74)	36.95 (0.76)	36.76 (0.86)	0.000^a^
**Outcome**					
ICU length of stay, days (mean (std))	6.88 (8.12)	6.10 (7.16)	7.03 (7.14)	8.26 (10.62)	0.000^a^
Hospital length of stay, days (mean (std))	13.80 (12.67)	12.60 (11.77)	14.65 (12.1)	15.06 (14.69)	0.000^a^
In-hospital mortality (%)	384 (15.8)	63 (5.68)	108 (14.38)	212 (38.27)	0.000^a^

CCU, coronary care unit; CSRU, cardiac surgery recovery unit; MICU, medical intensive care unit; SICU, surgical intensive care unit; TSICU, trauma surgical intensive care unit; SOFA, Sequential Organ Failure Assessment; GCS, Glasgow Coma Scale; ECI, Elixhauser Comorbidity Index.

a P-value < 0.05 indicates a significant difference.

The distribution of comorbidities across sepsis phenotypes was shown in Fig. 3A and C. Statistical analysis revealed that cardiovascular complications were common across all phenotypes. Notably, Phenotype C exhibited more severe multi-organ dysfunction, as indicated by a significantly higher ECI compared to those in Phenotypes A and B. Furthermore, Phenotype C was associated with higher prevalences of respiratory (71.66%), hematologic (49.10%), gastrointestinal (55.60%), and renal complications (70.94%). These patients demonstrated signs of renal impairment, characterized by the highest levels of creatinine and blood urea nitrogen and the lowest urine output; hemodynamic instability, reflected by the lowest mean arterial pressure; anemia or coagulopathy, indicated by the lowest hemoglobin levels; and metabolic acidosis, as evidenced by the lowest bicarbonate levels. Compared with Phenotype B, Phenotypes A and C showed coagulation abnormalities, as indicated by lower platelet counts. It highlighted significant differences in comorbidities and laboratory characteristics among the sepsis phenotypes.

We further investigated the relationship between sepsis phenotypes and SOFA scores. As illustrated in Fig. 3B, Phenotype C exhibited significantly higher SOFA scores, indicating greater disease severity compared to other phenotypes. As shown in Fig. 3D, patients within the same SOFA score strata could be classified into three phenotypes (A, B, and C), and this pattern was consistently observed across different score levels.

As illustrated in Supplementary Figs. S4 and S5, to investigate the association between subphenotypes and mortality, we provided time-resolved risk stratification scores (ranging from 0 to 2) across multiple time points (see Supplementary Methods 1.8 for details). We further analyzed the relationship between temporal risk levels and corresponding mortality. The results demonstrated that the proposed model was capable of generating timely and dynamic mortality risk alerts throughout the clinical course.

### Validation of sepsis subphenotypes

To evaluate the reproducibility and stability of the sepsis subphenotypes derived from the development cohort, we conducted external validation using the ODTGCNet model with identical configurations. It identified three subgroups with similar characteristics in validation cohort. Supplementary Table S4, Supplementary Figs. S6, S7 and S8 further demonstrate the reproducibility of the subphenotypes. The results obtained from the eICU dataset were largely consistent with those from MIMIC-III, further validating the robustness of the model and the effectiveness of phenotype classification. To confirm the robustness of the phenotypes across diverse clinical settings, stratified analyses were performed across different ICU types. The results indicated that phenotypes exhibited consistent distribution patterns and prognostic differences within all ICU subgroups (as shown in Supplementary Methods 1.9, Supplementary Figs. S9 and S10, and Supplementary Tables S5–S9).

### Heterogeneity in multi-organ states coupling patterns

**Fig. 4:**
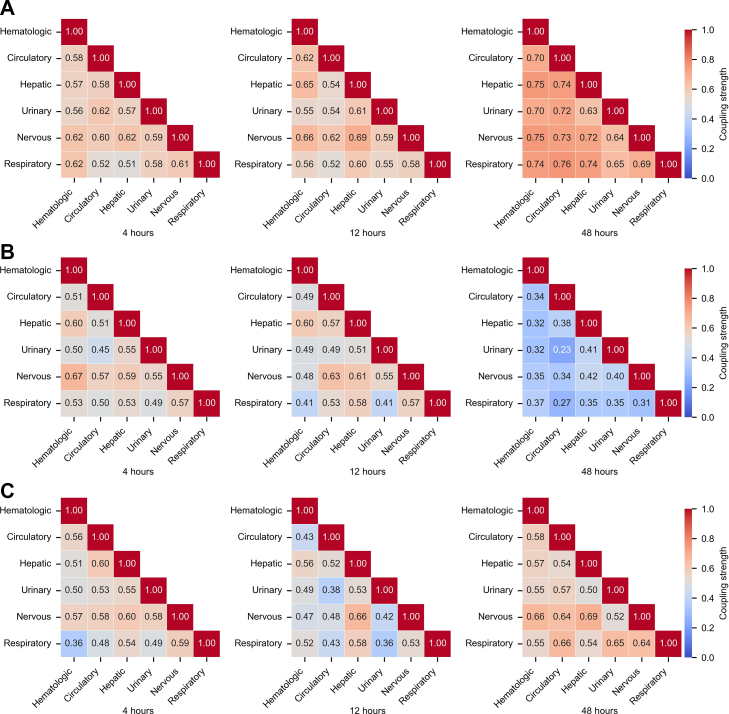
Heatmaps of temporal changes in inter-organ states coupling strength across phenotypes. A. Temporal coupling pattern for Phenotype A. B. Temporal coupling pattern for Phenotype B. C. Temporal coupling pattern for Phenotype C. Time points include 4, 12, and 48 h after sepsis diagnosis. The analyzed organ systems comprise the hematologic, circulatory, hepatic, urinary, nervous, and respiratory systems. Coupling strength refers to the similarity between organ system states. Heatmap color intensity represents the magnitude of coupling strength between organ systems. Distinct coupling patterns were observed among the three phenotypes, with statistically significant differences (P-value < 0.05).

As shown in Fig. 4 and Supplementary Fig. S11, significant differences in multi-organ system states coupling patterns were observed across phenotypes (P-value < 0.05). By incorporating patients’ actual clinical outcomes, distinct temporal patterns of multi-organ states coupling within the first 48 h post-diagnosis were identified. Phenotype A exhibited a gradual increase in inter-organ coordination over time, reaching the highest coupling strength at 48 h, and was associated with the lowest mortality. Phenotype B showed a progressive decline in coupling strength, indicating persistent desynchronization among organ systems. Phenotype C displayed a biphasic pattern, characterized by an initial decline followed by a marked increase in coupling strength during the late phase.

To further examine these findings, the organ state similarities between the kidney and lung systems were compared between Phenotypes A and C at the 48-h time point. Despite a comparable similarity score (0.65) in embedding space, clinical indicators revealed divergent physiological profiles. As illustrated in Supplementary Table S10, in Phenotype A, respiratory parameters remained within normal ranges, with low FiO_2_ requirements, preserved oxygenation capacity (high arterial oxygen partial pressure to inspired oxygen fraction ratio (PaO_2_/FiO_2_), normal oxygen saturation (SpO_2_)), near-normal renal function (creatinine, blood urea nitrogen (BUN)), and stable acid-base status (normal bicarbonate (HCO_3_^-^) and pH). In contrast, Phenotype C exhibited markedly elevated respiratory rates, increased FiO_2_ demand, impaired oxygenation (low PaO_2_/FiO_2_), significant renal dysfunction (elevated creatinine and BUN), and metabolic acidosis (low HCO_3_^-^, reduced pH). These findings indicate that the late-phase coupling observed in Phenotype C reflects a synchronized deterioration of multiple organ systems.

Furthermore, as illustrated in Supplementary Fig. S12, the coupling strength between each organ's current state (at 48 h) and its prior states was quantified. Phenotype A showed sustained high coupling throughout the observation period, peaking at 48 h. Phenotype B demonstrated persistently low intra-organ coupling, indicating a lack of temporal coherence. Phenotype C exhibited low early coupling followed by a sharp late-phase increase, suggesting a transition from divergent to highly synchronized organ responses. The organ coupling patterns of phenotypes across different SOFA groups (2–6, 7–10, ≥11) are shown in the Supplementary Fig. S13E. The results were consistent with the original analysis, confirming the robustness of the model performance.

### Clinical outcomes across different sepsis phenotypes

As shown in Table 1, Phenotype A exhibited the lowest in-hospital mortality (5.59%) and the shortest hospital length of stay (6.1 days) among all phenotypes. In contrast, Phenotype C had the highest in-hospital mortality (38.27%) and the longest hospital stay (8.3 days). To further assess survival outcomes during ICU admission, the KM survival analysis was performed to estimate the cumulative 28-day mortality risk following sepsis diagnosis.

**Fig. 5:**
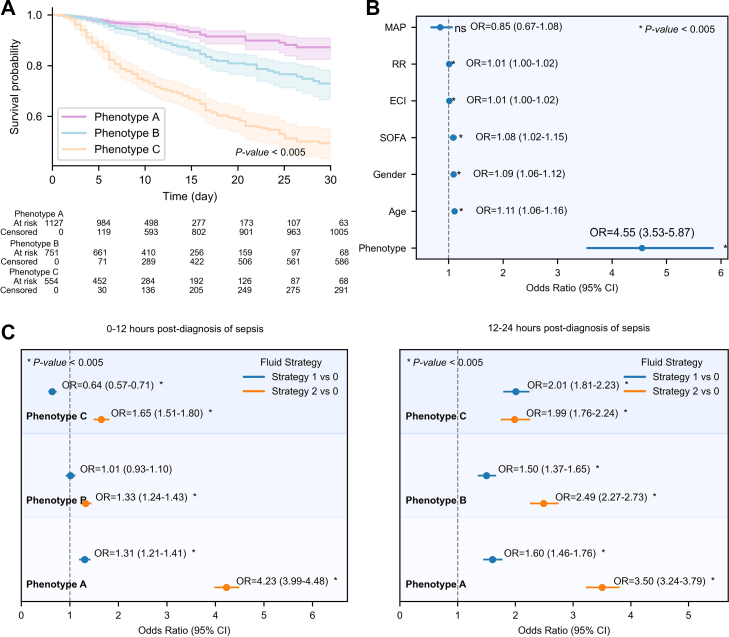
Survival analysis curves and Sequential Organ Failure Assessment (SOFA) trajectories for patients with different sepsis phenotypes. A. Kaplan–Meier survival curve: The *x*-axis represents the time since sepsis diagnosis, and the *y*-axis represents the survival probability. B. Forest plot of mortality risk associated with phenotypes and clinical variables. The *x*-axis shows odds ratios (OR), and the *y*-axis lists the variables. MAP, mean arterial pressure; RR, respiratory rate; ECI, Elixhauser Comorbidity Index. C. Odds ratios of mortality risk associated with fluid strategies across phenotypes. The analysis covers time intervals: 0–12 h and 12–24 h post sepsis diagnosis. ∗P-value < 0.05, statistically significant.

As illustrated in Fig. 5A, Phenotype A demonstrated the lowest 28-day cumulative mortality. In contrast, Phenotype C had a significantly higher cumulative mortality (P-value < 0.05). Notably, the survival curve for Phenotype C showed the steepest decline, indicating the fastest accumulation of mortality. It is clinically significant, suggesting that Phenotype C requires closer monitoring and potentially earlier intervention to prevent rapid deterioration. Phenotype B followed an intermediate trajectory, while Phenotype A displayed the most gradual decline, reflecting the slowest increase in cumulative mortality risk.

Fig. 3D demonstrates significant differences in mortality between phenotypes within the same SOFA category. For example, in the SOFA 2–6 group, mortality was 5.0% in Phenotype A compared with 36.0% in Phenotype C. Across all SOFA strata, mortality increased progressively from Phenotype A to C. As shown in Supplementary Fig. S13A–D, the AUROC for SOFA (baseline plus 48-h change) was 0.66 (95% CI [0.63, 0.68]), while APACHE II achieved an AUROC of 0.67 (95% CI [0.64, 0.70]). In comparison, the phenotype-based model reached an AUROC of 0.74 (95% CI [0.71, 0.77]), demonstrating superior predictive performance. As illustrated in Fig. 5B, using Phenotype C as an example, even after adjusting for baseline SOFA score, ECI, and other covariates, Phenotype C remained significantly associated with increased mortality (OR = 4.55, 95% CI [3.53, 5.87]). A similar association was observed after further adjustment for APACHE II score (Supplementary Table S11).

### Heterogeneous fluid treatment effect among subphenotypes

A likelihood ratio test comparing models with and without the interaction term between fluid therapy and phenotype demonstrated a statistically significant difference (P-value < 0.05). As shown in the Fig. 5C, both the direction and magnitude of response to fluid strategies varied significantly across phenotypes. Specifically, in Phenotype A, more aggressive fluid therapy, particularly Strategy 2 (high volume), was associated with significantly increased mortality risk at 0–12 h (OR = 4.23, 95% CI [3.99, 4.48]) and 12–24 h (OR = 3.50, 95% CI [3.24, 3.79]). In Phenotype B, fluid Strategies 1 (moderate volume) and 2 (high volume) were linked to modestly elevated mortality risk at 0–12 h (OR = 1.01, 95% CI [0.93, 1.10], OR = 1.33, 95% CI [1.24, 1.43]), which further increased at 12–24 h (both > 1.5). In Phenotype C, Strategy 1 (moderate volume) within 0–12 h was significantly associated with reduced mortality risk (OR = 0.54, 95% CI [0.57, 0.71]), whereas excessive fluid administration (Strategy 2) rapidly increased risk (OR = 1.65, 95% CI [1.51, 1.80]); both strategies were associated with higher mortality at 12–24 h.

To assess whether ICU type influences the response of different phenotypes to fluid strategies, a logistic regression model including an interaction term between ICU type and fluid strategy was constructed and compared with a simplified model without interaction using a likelihood ratio test. As shown in Supplementary Table S12, a statistically significant difference was observed only in Phenotype A during the 0–12 h window, while no significant interaction was identified for Phenotypes B and C. Supplementary Fig. S10E and F display the OR for different fluid strategies at 0–12 h in each phenotype, with and without the inclusion of cardiac surgery ICU (CSRU) patients, respectively. The two analyses demonstrated consistent trends. Notably, in Phenotype A, Strategies 1 (moderate volume) and 2 (high volume) were associated with slightly lower mortality risk compared to Strategy 0 (low volume). To further examine the applicability of fluid strategies in a more homogeneous population, a sensitivity analysis was performed in patients admitted exclusively to medical ICUs (MICU). Associations between fluid volume and in-hospital mortality were assessed for each phenotype at 0–12 and 12–24 h. As shown in Supplementary Fig. S14, the trend of response to fluid strategies remained generally consistent across phenotypes within the MICU subgroup, supporting the robustness of the findings.

### Model comprehensive clustering performance comparison

As shown in Supplementary Fig. S15 and Supplementary Table S13, the proposed ODTGCNet model consistently outperformed the SOTA models in clustering performance on both the MIMIC-III and eICU datasets. Supplementary Fig. S16 visualizes the clustering outcomes of DAEGC, SDCN, and ODTGCNet model. According to Supplementary Table S14, ODTGCNet enabled temporal risk stratification and early warning at critical time points (4-, 16-, and 32-h post-diagnosis). The ablation studies (Supplementary Tables S15 and S16 and Supplementary Figs. S17 and S18) validated the contribution of each model component, all of which enhance clustering performance. More details are shown in Supplementary Methods 1.10.

### Construction of a simple phenotype classifier

**Fig. 6:**
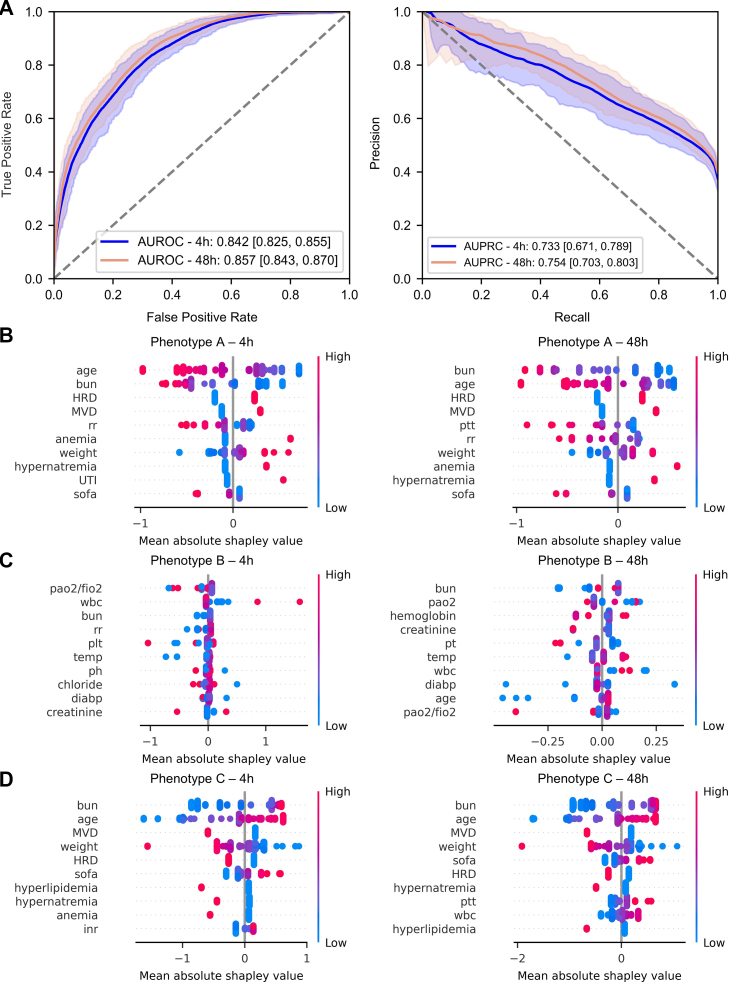
Construction of the phenotype classifier and SHapley Additive exPlanations (SHAP) analysis of feature importance. A. Receiver operating characteristic curves (ROC) of the phenotype classifier at 0–4 h and 44–48 h. The *x*-axis denotes the false positive rate (FPR), and the *y*-axis denotes the true positive rate (TPR). AUROC values are presented, with the area under each curve displayed. B–D. SHAP-based feature importance rankings for different phenotypes at 4 and 48 h. The *x*-axis shows the mean absolute SHAP values, and the *y*-axis lists the top 10 features ranked by importance. Point color represents feature value (red = high, blue = low), and SHAP magnitude indicates each feature's impact on the prediction.

As illustrated in Fig. 6, the phenotype classifier demonstrated favorable performance, achieving a higher AUROC at 48 h (0.86 (95% CI [0.84, 0.87])) than at 4 h (0.84 (95% CI [0.83, 0.86])), indicating improved predictive accuracy with longer observation periods. To assess the contribution of each feature to model predictions, SHAP was applied to interpret the XGBoost output. As shown in Fig. 6B–D and Supplementary Methods 1.11, SHAP summary plots illustrated the ranked feature importance and value distributions for Phenotypes A, B, and C at 4 and 48 h. For Phenotype A, key features at 4 h included age, BUN, heart-related diseases (HRD), multi-vessel disease (MVD), respiratory rate (RR), and hypernatremia. For Phenotype B, early predictions relied on PaO_2_/FiO_2_, white blood cell count (WBC), BUN, RR, and platelets, whereas creatinine, PaO_2_, hemoglobin, prothrombin time (PT), and temperature became more relevant at 48 h. For Phenotype C, BUN, age, MVD, weight, HRD, and SOFA score were important at 4 h, while HRD, SOFA, hypernatremia, hyperlipidemia, and WBC gained importance by 48 h. Higher values of certain features, such as HRD, were associated with increased SHAP values, indicating a stronger contribution to higher predicted risk. The web interfaces are shown in Supplementary Fig. S19.

## Discussion

In this study, we proposed a novel deep temporal graph clustering model to identify three sepsis phenotypes (A, B, and C) from multiple EHR datasets based on multi-organ interaction trajectories. Each phenotype exhibited distinct clinical characteristics and outcomes, reflecting different patterns of organ dysfunction and necessitating tailored management strategies. The identified phenotypes revealed dynamic heterogeneity in coupling strength and coordination among organ system states, providing clinical insights beyond conventional severity and organ dysfunction scores such as SOFA. Coupling strength refers to the similarity between organ system states. By quantifying organ system state coupling strength, clinicians can identify disease progression patterns, enabling personalized treatment. Specifically, Phenotype A was systemically coherent, characterized by highly synchronized recovery of organ system states that reflected a coordinated restorative process. This group included younger patients with fewer comorbidities, lower initial SOFA scores, and demonstrated the most favorable clinical prognosis. Phenotype B was persistently disordered, exhibiting sustained decoupling and prolonged asynchrony among organ systems, which indicated a disordered and dysregulated pathological state. Phenotype C was sequentially decoupled and catastrophic, showing a rapid transition from initial asynchronous dysfunction to synchronized collapse, which suggested a catastrophic coupling mechanism of organ failure. It was associated with the most severe multi-organ failure, the highest initial SOFA scores, and the poorest survival outcomes. To support clinical application, we developed a simple classifier and a web-based interface for early phenotype identification. We also examined differences in response to intravenous fluid therapy during the first 24 h after sepsis diagnosis. The results suggested that personalized fluid therapy should be considered in clinical practice to improve patient outcomes.

Sepsis is a clinical syndrome caused by infection, characterized by a dysregulated host response and life-threatening organ dysfunction.^2^ Organ dysfunction is defined by an increase in the SOFA score of more than two points.^1^ Based on this criterion, Xu et al. applied traditional clustering methods to SOFA trajectories and identified distinct sepsis phenotypes.^20^ It showed that these phenotypes differed in pathophysiological dynamics, disease progression, and the extent of organ damage. However, the SOFA score evaluates organ systems in an independent manner, limiting its ability to capture the complex and dynamic interactions between organs. During sepsis, organ systems are interconnected through intricate immunological, neurological, and metabolic pathways, forming a highly integrated network of inter-organ crosstalk.^21^^,^^22^ These networks can trigger bidirectional influences among organs and even systemic dysregulation.^21^^,^^41^^,^^42^ For example, the lung–kidney axis is considered a key mechanism underlying the co-occurrence of acute respiratory distress syndrome (ARDS) and acute kidney injury (AKI).^43^ The coexistence of AKI and acute lung injury (ALI) has been shown to significantly increase mortality in critically ill patients.^44^^,^^45^ Mounting evidence indicates that sepsis-associated organ dysfunction is not a linear or isolated process, but rather reflects a systemic state of multi-organ disintegration.^41^^,^^42^ Organ injuries often evolve asynchronously, with dysfunction in a single organ exerting secondary effects on distant organs through metabolic disturbances, inflammatory mediators, and neurohormonal signaling. This functional decoupling disrupts homeostatic regulation across organ systems and is widely recognized as a major contributor to clinical deterioration and poor outcomes in sepsis,^46^ consistent with the findings of our study (Phenotype B and C).

To quantify dynamic inter-organ interactions in sepsis, we developed a multi-organ interaction graph and identified patient phenotypes based on their interaction trajectories. The temporal evolution of organ states coupling strength (Fig. 4) revealed distinct patterns of synchrony and asynchrony in organ system states over time. For instance, Phenotype C exhibited a “decoupling followed by resynchronization” pattern, indicating that early systemic dysregulation may rapidly progress into coordinated multi-organ failure. This progression was not readily captured by the SOFA scoring system. Existing clustering methods based on SOFA components failed to reflect dynamic inter-organ synergy or antagonism, limiting their ability to detect such phenotypes.^9^^,^^16^^,^^18^^,^^19^^,^^47^ In contrast, our model explored temporal coupling among organ systems, moving beyond isolated trajectory analysis. For example, mortality increased markedly from Phenotype A to C despite similar SOFA scores (Fig. 3D), suggesting that the proposed model identified latent risk heterogeneity not reflected by conventional scoring. Organ-specific dynamics varied fundamentally across phenotypes, even when SOFA scores were comparable, and such differences were not captured by traditional metrics. Instead, the present method delineated dynamic organ interactions, uncovered underlying pathophysiological heterogeneity, and demonstrated potential to redefine the boundaries of high-risk populations, offering implications for precision resource allocation in critical care. While dynamic SOFA and APACHE II scores provide prognostic information,^48^ phenotype-based stratification is able to offer additional predictive value beyond them (Fig. 5B, Supplementary Fig. S13A–D, and Supplementary Table S11). Further analyses revealed that significant differences in organ interaction patterns persisted between phenotypes after stratification by SOFA score ranges (Supplementary Fig. S13E). This consistency supported the interpretation that organ states coupling dynamics were intrinsic to the phenotypes rather than artifacts of SOFA-based grouping. Additionally, to quantify the dynamic prognostic risk of patients, we introduced a time-resolved risk stratification indicator, which demonstrated the model's ability to dynamically capture the evolving death risk trends across different phenotypes (as shown in Supplementary Fig. S5 and S7).

Fluid resuscitation is a cornerstone of sepsis management.^7^ Identifying distinct fluid responsiveness patterns may facilitate more individualized and effective resuscitation. Phenotype A exhibited high sensitivity to fluid input (Fig. 5). Excessive fluid administration may disrupt the coordinated recovery of organ systems, leading to perfusion imbalance and increased mortality risk. Previous studies have identified fluid overload within the first 24 h as an independent predictor of mortality.^49^, ^50^, ^51^ Patients with this phenotype may benefit from a restrictive fluid strategy to preserve internal homeostasis and avoid interfering with ongoing recovery. Phenotype B showed attenuated responsiveness to fluid therapy. Due to decoupled organ dynamics and impaired regulatory mechanisms, additional fluid had minimal impact on mortality.^52^ For these patients, fluid management should rely more heavily on assessments of volume responsiveness to prevent ineffective or harmful accumulation. Phenotype C demonstrated a narrow therapeutic window for fluid responsiveness. Moderate early fluid resuscitation may improve perfusion (e.g., Strategy 1 (moderate volume) reduced mortality within the first 12 h), whereas delayed or excessive fluid loading increased the risk of volume overload, pulmonary complications, and rapid multiorgan failure.^52^ A prior analysis of 23,513 patients with severe sepsis and septic shock showed that on ICU Day 1, mortality slightly decreased with each liter of fluid administered in the low-volume range, but increased significantly with each additional liter beyond the high-volume threshold.^49^ The cohort's average mortality was approximately 26%, comparable to that observed in Phenotype C. Consistent with our findings, a U-shaped relationship was observed between early fluid volume and mortality in Phenotype C, underscoring the need for precise control of both timing and total fluid volume in this subgroup.

To assess whether ICU type influences phenotype classification and fluid responsiveness, we conducted a series of stratified and sensitivity analyses. All three phenotypes were observed across ICU types, and within each ICU, a consistent mortality gradient was maintained (lowest in Phenotype A, highest in Phenotype C), suggesting that the clustering captured temporal patterns of disease progression rather than admission source or unit-specific features. Further supporting this, univariate logistic regression models demonstrated that phenotype membership yielded significantly better discrimination of in-hospital mortality compared to ICU type, underscoring its independent prognostic value. Recognizing that postoperative cardiac surgery patients (e.g., CSRU) might introduce heterogeneity in treatment response, we conducted a series of sensitivity analyses. A logistic regression model including an interaction term between ICU type and fluid strategy revealed a statistically significant interaction only in Phenotype A during the first 12 h, suggesting a possible modifying effect of ICU type on fluid responsiveness in this group. However, when CSRU patients were excluded, the interaction effect disappeared entirely, and the odds ratio patterns for fluid strategies remained largely unchanged. This indicates that the initial interaction likely resulted from compositional bias rather than a true modification effect. Furthermore, a subgroup analysis limited to MICU patients confirmed that phenotype-dependent differences in fluid responsiveness persisted in a more clinically homogeneous population. These findings reinforce that the identified phenotypes primarily reflect dynamic inter-organ interaction patterns and demonstrate stable treatment–response profiles across ICU types, supporting their applicability for individualized therapeutic stratification.

There were several limitations in this study. First, all cohorts were derived from multicenter databases in the United States, and variability in ICU admission practices may limit the generalizability of the identified phenotypes to other healthcare settings.^53^ Although MIMIC-III and eICU are among the most comprehensive and publicly available structured ICU datasets, their broad temporal spans may introduce bias due to evolving definitions of sepsis and shifts in clinical management over time. Furthermore, the mechanism of missing data may not be completely random, posing a risk of selection bias. Future studies should incorporate more advanced techniques for handling missing data and utilize more recent, representative datasets for external validation, thereby enhancing both the timeliness and applicability of the findings. Second, to enable timely and generalizable patient stratification in resource-limited clinical settings, our model was developed using only routinely collected clinical data. However, the absence of multi-omics data constrained the investigation of underlying biological mechanisms associated with each phenotype. Previous studies have emphasized the importance of mechanistic biomarkers in improving the biological interpretability of clinical phenotypes.^16^^,^^54^ Future research should integrate transcriptomic and metabolomic data to identify phenotype-specific inter-organ interaction hubs, such as the renal and coagulation axis, assess differential pathway enrichment, and inform the development of network-based targeted interventions. Third, as fluid management strategies were evaluated retrospectively, the association between fluid volume and mortality should be interpreted as correlational rather than causal. Despite the use of propensity score matching to reduce confounding, residual bias may persist, particularly due to indication bias, whereby more severely ill patients receive greater fluid volumes. Clinical interventions such as vasopressors and mechanical ventilation may exert significant influences on organ states. Future research should consider incorporating prior information, including ICU type and key treatment strategies, into organ interaction modeling to identify phenotypes enriched with specific therapeutic characteristics and support personalized treatment planning. In addition, fluid strategies analysis may be affected by immortal time bias. A 24-h survival threshold was applied to reduce this risk. The use of time-dependent methods, such as time-varying Cox regression, may further improve the accuracy of assessing the dynamic relationship between fluid therapy and clinical outcomes. Moreover, the study only evaluated total daily fluid input and did not consider important aspects of fluid management, such as fluid balance strategies or fluid responsiveness. The fluid intervention strategy did not strictly distinguish between resuscitation fluids and “fluid creep” (e.g., hypertonic saline, glucose-containing solutions, or mannitol). These fluids may serve different therapeutic purposes and sometimes reflect disease severity rather than true resuscitative practice.^55^ A more precise distinction between resuscitation and non-resuscitation fluids would enhance the clinical interpretability of the findings and better inform causal inferences. Future research should integrate high-frequency nursing documentation and medication administration data to more comprehensively characterize fluid management across different phenotypes.^55^ Fourth, as our study was based on a deep learning-based model, coupling strength was derived from deep embeddings of multi-organ interaction trajectories, and its changes could not be consistently mapped to a single clinical variable in every patient, limiting clinical interpretability. Enhancing the clinical interpretability of the model remains a key direction for future research. Finally, this study restricted clustering to patients’ initial ICU admissions to prevent data non-independence from multiple stays. However, this may omit dynamic changes during subsequent ICU episodes. Future work should incorporate repeated admissions to assess trajectory continuity and phenotype stability. Prospective multicenter randomized controlled trials are ultimately required to validate the clinical effectiveness of phenotype-guided fluid management strategies.

In conclusion, we identified three phenotypes based on dynamic multi-organ interaction trajectories, each exhibiting significant heterogeneity in baseline characteristics, organ system state coupling patterns, and clinical outcomes. Beneficial fluid management strategies varied across different phenotypes, highlighting the need for targeted interventions and further providing valuable guidance for clinical decision-making. Defined organ system coupling patterns enables precise prognostic stratification and individualized fluid therapy guidance, providing a foundation for elucidating multi-organ dysfunction mechanisms and advancing precision therapies. Using a simple classifier and web interface, clinicians can easily identify sepsis phenotypes at the bedside and tailor intravenous fluid therapy accordingly. Further prospective studies are necessary to validate our findings and evaluate the practical applicability.

## Contributors

XF, GN, LF, and QP conceived and designed the study. XF developed and validated the deep learning system supervised by GN and LF with clinical input from ZZ and LS. XF and JZ did the statistical analysis. LS, JZ, and XY also contributed to computational analysis and validations. ZZ and QP provided critical reading and suggestions. XF drafted the manuscript with input from LS, JZ, and LS. All authors contributed to revisions of the manuscript. The final version of the manuscript was read and approved by all authors. GN, LF, and QP contributed equally to the work as senior authors. The corresponding author and senior authors had full access to all data. All authors had full access to all the data in the study and had final responsibility for the decision to submit for publication. XF, GN, LF, and QP had accessed and verified the underlying data and had final responsibility for the decision to submit for publication.

## Data sharing statement

All de-identified data and codes that support the findings of this study are available from the corresponding author upon reasonable request (gmning@zju.edu.cn). The packaged classifier application can be found at https://github.com/ppwqmyxtc/sepsis_subphenotypes.

## Declaration of interests

All authors declare no competing interests.

## Appendix A Supplementary data

Supplementary Metrials
