# Supplementary material for: Subphenotyping sepsis based on organ interaction trajectory using a deep temporal graph clustering model: a retrospective cohort study
Source: eClinicalMedicine. 2025 Dec 5;90:103691. doi: 10.1016/j.eclinm.2025.103691 (PMC12766501; doi:10.1016/j.eclinm.2025.103691)
Supplement: Supplementary Metrials [file mmc1.docx]

Supplementary Appendix

Supplementary to: Subphenotyping sepsis based on organ interanion trajectory using a deep temporal graph clustering model: a retrospective cohort study.

[1. Supplementary Methods 1](#_Toc209176717)

[1.1 Datasets and data preprocessing 1](#_Toc209176718)

[1.1.1 Data source 1](#_Toc209176719)

[1.1.2 Sensitivity analysis for missing and outlier data handling 2](#_Toc209176720)

[1.2 ODTGCNet model development and validation 2](#_Toc209176721)

[1.2.1 Model construction 2](#_Toc209176722)

[1.2.2 Model implementation settings 6](#_Toc209176723)

[1.3 Model evaluation metrics 6](#_Toc209176724)

[1.3.1 Number of clusters determining 6](#_Toc209176725)

[1.3.2 Silhouette Coefficient calculating 7](#_Toc209176726)

[1.3.3 Davies-Bouldin index calculating 7](#_Toc209176727)

[1.4 Experiments design 7](#_Toc209176728)

[1.4.1 Baseline models settings 7](#_Toc209176729)

[1.4.2 Ablation experiments settings 8](#_Toc209176730)

[1.5 Web interface construction 8](#_Toc209176731)

[1.6 Intravenous fluid analysis across phenotypes 8](#_Toc209176732)

[1.7 The impact of ICU type on phenotype-specific fluid strategies 10](#_Toc209176733)

[1.8 Relationships between temporal risk stratifications and mortality 10](#_Toc209176734)

[1.9 Phenotype variation across different ICU types in sepsis patients 11](#_Toc209176735)

[1.10 Model comprehensive clustering performance comparison 13](#_Toc209176736)

[1.11 Construction of a simple phenotype classifier 14](#_Toc209176737)

[2. Supplementary Figures 14](#_Toc209176738)

[Supplementary Fig. S1 14](#_Toc209176739)

[Supplementary Fig. S2 15](#_Toc209176740)

[Supplementary Fig. S3 16](#_Toc209176741)

[Supplementary Fig. S4 17](#_Toc209176742)

[Supplementary Fig. S5 18](#_Toc209176743)

[Supplementary Fig. S6 19](#_Toc209176744)

[Supplementary Fig. S7 20](#_Toc209176745)

[Supplementary Fig. S8 21](#_Toc209176746)

[Supplementary Fig. S9 22](#_Toc209176747)

[Supplementary Fig. S10 23](#_Toc209176748)

[Supplementary Fig. S11 24](#_Toc209176749)

[Supplementary Fig. S12 25](#_Toc209176750)

[Supplementary Fig. S13 26](#_Toc209176751)

[Supplementary Fig. S14 27](#_Toc209176752)

[Supplementary Fig. S15 27](#_Toc209176753)

[Supplementary Fig. S16 28](#_Toc209176754)

[Supplementary Fig. S17 28](#_Toc209176755)

[Supplementary Fig. S18 29](#_Toc209176756)

[Supplementary Fig. S19 30](#_Toc209176757)

[3. Supplementary Tables 30](#_Toc209176758)

[Supplementary Table S1 30](#_Toc209176759)

[Supplementary Table S2 31](#_Toc209176760)

[Supplementary Table S3 32](#_Toc209176761)

[Supplementary Table S4 32](#_Toc209176762)

[Supplementary Table S5 33](#_Toc209176763)

[Supplementary Table S6 33](#_Toc209176764)

[Supplementary Table S7 34](#_Toc209176765)

[Supplementary Table S8 34](#_Toc209176766)

[Supplementary Table S9 35](#_Toc209176767)

[Supplementary Table S10 36](#_Toc209176768)

[Supplementary Table S11 36](#_Toc209176769)

[Supplementary Table S12 36](#_Toc209176770)

[Supplementary Table S13 36](#_Toc209176771)

[Supplementary Table S14 36](#_Toc209176772)

[Supplementary Table S15 37](#_Toc209176773)

[Supplementary Table S16 37](#_Toc209176774)

[Supplementary Table S17 37](#_Toc209176775)

[Supplementary Table S18 37](#_Toc209176776)

[Supplementary Reference 37](#_Toc209176777)

# **1. Supplementary Methods**

## **1.1 Datasets and data preprocessing**

### **1.1.1 Data source**

The development cohort was derived from the Medical Information Mart for Intensive Care III database (MIMIC-III) database, comprising intensive care unit (ICU) admissions from 2001 to 2012. The external validation cohort was obtained from the multicenter eICU collaborative research database (eICU), which includes admissions from 2014 and 2015.

The MIMIC-III database is a publicly available, de-identified single-center ICU database comprising 38,597 adult patients (aged $\geq$16 years) to the Beth Israel Deaconess Medical Center in Boston, Massachusetts, between 2001 and 2012. The dataset spans a broad range of clinical domains, including demographics, vital signs (typically recorded hourly), laboratory measurements, medication administration, nursing notes, fluid balance, imaging and diagnostic codes, procedure records, and discharge or mortality outcomes. Each ICU stay contains, on average, approximately 4,579 charted observations and 380 laboratory tests. The database includes around 40 relational tables, enabling integrated analyses and machine learning applications. Widely cited in critical care research, MIMIC-III has been used for outcome prediction, quality improvement, clinical education, and algorithm development^1^.

The eICU database is a multi-center ICU database developed through a collaboration between Philips Healthcare and the MIT Laboratory for Computational Physiology. It contains 200,859 ICU admissions from 139,367 unique patients across 208 hospitals in the United States during 2014-2015. The database captures de-identified, high-resolution clinical data, including vital signs, care plans, acute physiology and chronic health evaluation (APACHE) severity scores, admission diagnoses, treatments, and medication administration. eICU comprises approximately 31 CSV tables. These tables provide detailed records of pharmacologic interventions, nursing assessments, diagnostic codes, APACHE score components, and therapeutic procedures. Owing to its multicenter design and high temporal granularity, eICU has been widely utilized in machine learning and statistical modeling studies, including predictions of ICU mortality, length of stay, and mechanical ventilation duration. Its diverse institutional sources enhance the generalizability and robustness of research findings ^2^.

We further analyzed and clarified the demographic and clinical representativeness of the two datasets:

**(1) Demographic representativeness**

Both MIMIC-III and eICU are derived from large academic medical centers in the United States and encompass diverse patient populations across regions and clinical settings.

**(2) Clinical diversity**

The two cohorts include a wide range of ICU types (medical, surgical, trauma, etc.), with clinical scores and vital signs capturing varying degrees of illness severity. Hemodynamic parameters and organ function indicators reflect the heterogeneous clinical manifestations of sepsis.

**(3) Limitations and generalizability**

Both datasets are limited to U.S. hospitals and may not fully represent patient populations or healthcare contexts in other regions or countries. Additionally, the long-time span of the data (e.g., MIMIC-III spans 2001-2012) may introduce variability due to evolving diagnostic criteria and treatment strategies. In summary, despite these limitations, the use of two large and heterogeneous cohorts provides a robust foundation for model development and validation, enhancing the model’s generalizability and clinical relevance.

### **1.1.2 Sensitivity analysis for missing and outlier data handling**

To assess the robustness of our clustering results to data preprocessing strategies, we conducted a sensitivity analysis addressing both missing data and outliers. In the primary analysis, missing values were imputed using a combination of forward filling, backward filling, and cohort-level mean substitution. As a complementary approach, we applied Multiple Imputation by Chained Equations (MICE) to account for potential imputation bias due to data missingness mechanisms.

For outlier handling, in addition to the interquartile range (IQR)-based exclusion of extreme values used in the main analysis, we further implemented Winsorization to cap variable values at predefined percentile thresholds, thereby reducing the influence of extreme observations without discarding data.

The clustering results generated by the ODTGCNet model remained stable across different preprocessing strategies. As shown in Supplementary Table S17, clustering performance metrics—including the Davies-Bouldin Index and Silhouette Coefficient—were comparable between the baseline method, MICE-imputed data, and Winsorized data. No significant shifts were observed in cluster assignments, suggesting minimal sensitivity of the model to variation in imputation or outlier treatment.

These findings indicate that our clustering framework is robust to reasonable variations in data preprocessing, supporting the reliability and generalizability of the phenotypes identified.

## **1.2 ODTGCNet model development and validation**

### **1.2.1 Model construction**

#### **Conditional variational autoencoder construction**

The proposed AGCVAE unit is shown in Fig. 3 (C). The CVAE is utilized to learn data information from the graph and generate predictive labels. CVAE can be viewed as a supervised extension of the variational autoencoder (VAE), where the input consists of raw data $x\in X$ and corresponding labels $y\in Y$. By incorporating label information into both the encoder and decoder, CVAE achieves precise control over the generation of data belonging to specific categories. The encoder of the CVAE can be represented as $q_{\varphi}\left( z | x,y \right)$, with input vectors $X$ and $Y$. The decoder is represented as $p_{\theta}\left( x|y,z \right)$, with input vectors $Y$ and $Z$, where $Y=\hat{Y}$ represents the predicted labels for patient outcomes. The encoder transforms the input $[X_{m},\hat{Y}_{m}]$ into the latent vector $Z_{m}$, and the input is reconstructed through the decoder as $\tilde{X}_{m}$, as shown in Eq. (2).

$$\begin{aligned} Z_{m}=E\left( [X_{m},\hat{Y}_{m}] \right)\#\left( 1 \right) \end{aligned}$$

$$\begin{aligned} \tilde{X}_{m}=D\left( [Z_{m},\hat{Y}_{m}] \right)\#\left( 2 \right) \end{aligned}$$

where $E\left( \cdot\right)$ and $D\left( \cdot\right)$ represent the mapping relationships of the encoder and decoder, respectively. The encoder is composed of $N$ fully connected layers, with the output of each layer denoted as $\left\{ H_{1},H_{2},\ldots,H_{n},\ldots,H_{N} \right\}$.

Essentially, the CVAE models the conditional probability $p(x|y)$ parametrically, with the optimization objective focused on maximizing the log-likelihood estimate $logp_{\theta}\left( x|y \right)$, as shown in Eq. (3). This optimization process is achieved by maximizing the evidence lower bound (ELBO) $\mathcal{L}_{\mathrm{ELBO}}$, as depicted in Eq. (4). Furthermore, the loss function of the CVAE is presented in Eq. (5).

$$logp_{\theta}\left( x|y \right)=\left( \int q_{\varphi}\left( z | x,y \right)dz \right)\log p_{\theta}\left( x|y \right)$$

$$=\int q_{\varphi}\left( z | x,y \right)\log p_{\theta}\left( x|y \right)dz$$

$$=\int q_{\varphi}\left( z | x,y \right)\log\frac{p_{\theta}\left( z,x,y \right)}{p_{\theta}\left( z|x,y \right)p_{\theta}\left( y \right)}dz$$

$$=\int q_{\varphi}\left( z | x,y \right)\log\frac{q_{\varphi}\left( z | x,y \right)}{p_{\theta}\left( z|x,y \right)}\frac{p_{\theta}\left( z,x,y \right)}{q_{\varphi}\left( z | x,y \right)p_{\theta}\left( y \right)}dz$$

$$=\int q_{\varphi}\left( z | x,y \right)\log\frac{q_{\varphi}\left( z | x,y \right)}{p_{\theta}\left( z|x,y \right)}dz+\int q_{\varphi}\left( z | x,y \right)\log\frac{p_{\theta}\left( z,x,y \right)}{q_{\varphi}\left( z | x,y \right)p_{\theta}\left( y \right)}dz$$

$$\begin{aligned} =KL\left( q_{\varphi}\left( z | x,y \right)\parallel p_{\theta}\left( z|x,y \right) \right)+\mathcal{L}_{\mathrm{ELBO}}\left( p_{\theta},q_{\varphi} \right)\geq\mathcal{L}_{\mathrm{ELBO}}\left( p_{\theta},q_{\varphi} \right)\#\left( 3 \right) \end{aligned}$$

$$\mathcal{L}_{\mathrm{ELBO}}\left( p_{\theta},q_{\varphi} \right)=\int q_{\varphi}\left( z | x,y \right)\log\frac{p_{\theta}\left( z,x,y \right)}{q_{\varphi}\left( z | x,y \right)p_{\theta}\left( y \right)}dz$$

$$=\int q_{\varphi}\left( z | x,y \right)\log\frac{p_{\theta}\left( x | y,z \right)p_{\theta}\left( z | y \right)p_{\theta}\left( y \right)}{q_{\varphi}\left( z | x,y \right)p_{\theta}\left( y \right)}dz$$

$$=\int q_{\varphi}\left( z | x,y \right)\log\frac{p_{\theta}\left( z | y \right)}{q_{\varphi}\left( z | x,y \right)}dz+\int q_{\varphi}\left( z | x,y \right)\log p_{\theta}\left( x | y,z \right)dz$$

$$\begin{aligned} =-KL\left( q_{\varphi}\left( z | x,y \right)\parallel p_{\theta}\left( z|y \right) \right)+\mathbb{E}_{q_{\varphi}\left( z | x,y \right)}\left[ \log p_{\theta}\left( x|y,z \right) \right]\#\left( 4 \right) \end{aligned}$$

$$\begin{aligned} \mathcal{L}_{CVAE}\left( x,y;\theta,\varphi\right)=\frac{1}{L}\sum_{l=1}^{L} \log p_{\theta}\left( x|y,z \right)-KL\left( q_{\varphi}\left( z | x,y \right)\parallel p_{\theta}\left( z|y \right) \right)\#\left( 5 \right) \end{aligned}$$

In the CVAE loss function $\mathcal{L}_{CVAE}\left( x,y;\theta,\varphi\right)$, the first term represents the reconstruction error, while the second term denotes the Kullback-Leibler (KL) divergence between the posterior distribution $q_{\varphi}\left( z | x,y \right)$ and the prior distribution $p_{\theta}\left( z|y \right)$.

The latent variable $z$ is statistically independent of the input variable, that is, $p_{\theta}\left( z|y \right)=p_{\theta}\left( z \right)$, where $p\left( z \right)$ is the prior distribution of the latent variable, given as the standard normal distribution $\mathcal{N}(0,I)$. As shown in Eq. (6), by employing the reparameterization trick, the model can perform backpropagation of errors through Gaussian latent variables, thereby enabling the efficient execution of the CVAE training process via gradient descent algorithms.

$$\begin{aligned} \left\{ \begin{aligned} z=\mu_{z}+\sigma_{z}⨀\epsilon\\ \epsilon\sim\mathcal{N}\left( 0,I \right) \end{aligned} \right.\#\left( 6 \right) \end{aligned}$$

#### **Adaptive information fusion layer design**

To achieve a comprehensive integration of the multi-level deep representations obtained by the encoder and the multi-level structural information representations captured by the GAT, an adaptive information fusion strategy has been proposed. This strategy incorporates an attention mechanism to generate a more holistic data representation.

$$\begin{aligned} \left\{ \begin{aligned} \#Q=W_{Q}\left[ H^{n}, {\tilde{G}^{n}}^{'} \right] \\ \#K=W_{K}\left[ H^{n}, {\tilde{G}^{n}}^{'} \right] \\ \#V=W_{V}\left[ H^{n}, {\tilde{G}^{n}}^{'} \right] \\ \#Attention\left( Q,K,V \right)=softmax\left( \frac{{QK}^{T}}{\sqrt{d_{k}}} \right)V \end{aligned} \right.\#\left( 7 \right) \end{aligned}$$

For the $n^{th}$ adaptive information fusion layer, the inputs consist of the deep representation $H^{n}$ output by the $n^{th}$ layer of the CVAE encoder and the output ${\tilde{G}^{n}}^{'}$ of the GAT at the corresponding level. The output of the fusion layer is denoted as $\left[ \tilde{H}^{n},\tilde{G}^{n} \right]$. The computation of the adaptive information fusion layer adheres to Eq. (7), where $W_{Q}$, $W_{K}$, and $W_{V}$ are linear transformation matrices, and $d_{k}$ is the dimensionality of the vectors in matrix $W_{Q}$. The $softmax$ function is used for normalization.

#### **Graph attention network construction**

In this study, GAT units are employed to extract information from graph-structured data. The self-attention layers in GAT enable nodes to capture features from their neighboring nodes while assigning differentiated weights, thereby achieving refined feature representation. For the $n^{th}$ layer of the GAT network, the input is the output from the adaptive information fusion layer, denoted as $\tilde{G}^{n}\in R^{F}$, represented as $\tilde{G}^{n}=\left\{ \tilde{g}_{1}^{n},\tilde{g}_{2}^{n},\ldots,\tilde{g}_{l}^{n},\ldots,\tilde{g}_{L}^{n} \right\}$, where $F$ is the feature dimension, $l=[1,2,\ldots,L]$ indexes the nodes, and $L$ is the total number of nodes. The output of the GAT layer is given by ${\tilde{G}^{n}}^{'}=\left\{ {\tilde{g}_{1}^{n}}^{'},{\tilde{g}_{2}^{n}}^{'},\ldots,{\tilde{g}_{l}^{n}}^{'},\ldots,{\tilde{g}_{L}^{n}}^{'} \right\}$, where ${\tilde{G}^{n}}^{'}\in R^{F^{'}}$. The attention layer in GAT captures the interaction between node pairs $\left( i,j \right)$ by computing the attention coefficient $\alpha_{ij}$, as defined in Eq. (9).

$$\begin{aligned} e_{ij}{=a}^{T}[W\tilde{g}_{i}\parallel W\tilde{g}_{j}]\#\left( 8 \right) \end{aligned}$$

$$\begin{aligned} \alpha_{ij}=softmax\left( e_{ij} \right)=\frac{\exp\left( LeakyReLU\left( e_{ij} \right) \right)}{\sum_{k\in\mathcal{N}_{i}} \exp\left( LeakyReLU\left( e_{ik} \right) \right)}\#\left( 9 \right) \end{aligned}$$

where, $\mathcal{N}_{i}$ represents the neighborhood set of nodes $i$, and $e_{ij}$ quantifies the importance of node $j$ to node $i$. The parameter $a\in R^{{2F}^{'}}$ denotes a single-layer feedforward neural network, while $W\in R^{F^{'}\times F}$ is the weight matrix. The symbol $\parallel$ denotes the concatenation operation. $LeakyReLU$ is chosen as the activation function to introduce nonlinearity, and $softmax$ is applied for normalization.

Finally, based on the attention weights, the neighborhood representations of each node are linearly aggregated to generate the node’s output representation, as defined in Eq. (10).

$$\begin{aligned} \tilde{g}_{i}^{'}=\sigma\left( \sum_{j\in\mathcal{N}_{i}} \alpha_{ij}W\tilde{g}_{j} \right)\#\left( 10 \right) \end{aligned}$$

#### **Temporal information extraction layer construction**

To enable the ODTGCNet model to capture temporal information in disease progression, a temporal information extraction layer based on an LSTM network is designed to deeply explore and understand the time-dependent patterns of disease features. At time step $t$, the input to the LSTM unit is the output of the GAT unit, denoted as $\tilde{G}_{t-1}^{N^{'}}$, while the outputs are $h_{t}$ and $\hat{Y}_{t}$. The $\hat{Y}_{t}$. represents the model’s prediction of the patient outcome at time step $t$, which serves as the conditional input for the CVAE at the next time step $t+1$. The computation process is defined in Eq. (11).

$$\begin{aligned} \left\{ \begin{aligned} \#f_{t}=\sigma\left( w_{f}\cdot{[h_{t-1},\tilde{G}}_{t-1}^{N^{'}}]+b_{f} \right) \\ \#i_{t}=\sigma\left( w_{i}\cdot{[h_{t-1},\tilde{G}}_{t-1}^{N^{'}}]+b_{i} \right) \\ \#o_{t}=\sigma\left( w_{o}\cdot{[h_{t-1},\tilde{G}}_{t-1}^{N^{'}}]+b_{o} \right) \\ \#\tilde{C}_{t}=\tanh\left( w_{c}\cdot{[h_{t-1},\tilde{G}}_{t-1}^{N^{'}}]+b_{c} \right) \\ \#{C_{t}=f}_{t}*\left( C_{t-1}^{D}+att\tilde{C}_{t-1} \right)+i_{t}*\tilde{C}_{t} \\ \#h_{t}=o_{t}* \tanh\left( C_{t} \right) \end{aligned} \right.\#\left( 11 \right) \end{aligned}$$

Where $w$ and $b$ represent the network’s weight matrix and bias parameters, respectively, while $\sigma$ denotes the $Sigmoid$ function.

#### **Output layer construction**

The output of the ODTGCNet model is denoted as $C_{t}$, representing the clustering results at time step $t$. The output layer of the model is formulated in Eq. (12).

$$\begin{aligned} C=\mathrm{argmax}_{j}q_{ij}\#\left( 12 \right) \end{aligned}$$

where $q_{ij}$ denotes the similarity between the embedded point $z_{i}$ and the cluster center $\mu_{j}$.

#### **Target-oriented self-supervised loss function construction**

In the unsupervised learning task, the clustering objective function of CVAE is formulated in Eq. (13).

$$\begin{aligned} \mathcal{L}_{C}=KL\left( P\parallel Q \right)=\sum_{i} \sum_{u} p_{iu}\log\frac{p_{iu}}{q_{iu}}\#\left( 13 \right) \end{aligned}$$

$$\begin{aligned} q_{ij}=\frac{\left( 1+\left\| z_{i}-\mu_{j} \right\|^{2} \right)^{-1}}{\sum_{j} \left( 1+\left\| z_{i}-\mu_{j} \right\|^{2} \right)^{-1}}\#\left( 14 \right) \end{aligned}$$

$$\begin{aligned} p_{ij}=\frac{\frac{q_{ij}^{2}}{\sum_{i} q_{ij}}}{\sum_{j} \left( \frac{q_{ij}^{2}}{\sum_{i} q_{ij}} \right)}\#\left( 15 \right) \end{aligned}$$

For the GAT model, the clustering objective function is defined in Eq. (16). To measure the reconstruction error between $A$ and $\hat{A}$, a cross-entropy loss function $\mathcal{L}_{AR}$ is utilized, as defined in Eq. (18).

$$\begin{aligned} \mathcal{L}_{G}=KL\left( P\parallel{\tilde{G}^{N}}^{'} \right)=\sum_{i} \sum_{u} p_{iu}\log\frac{p_{iu}}{{{\tilde{G}^{N}}^{'}}_{iu}}\#\left( 16 \right) \end{aligned}$$

$$\begin{aligned} \hat{A}_{ij}=sigmoid\left( {z_{i}}^{T}z_{j} \right)\#\left( 17 \right) \end{aligned}$$

$$\begin{aligned} \mathcal{L}_{AR}=\sum_{l=1}^{L} loss\left( A_{ij},\hat{A}_{ij} \right)\#\left( 18 \right) \end{aligned}$$

Furthermore, as described in Eq. (19), a cross-entropy loss function is employed to quantify the discrepancy between the model’s predicted outcomes and the actual patient outcome labels.

$$\begin{aligned} \mathcal{L}_{S}=-\frac{1}{M}\sum_{1}^{M} y_{t}\log\hat{y}_{t}+\left( 1-y_{t} \right)\log\left( 1-\hat{y}_{t} \right)\#\left( 19 \right) \end{aligned}$$

Considering the overall objective of the ODTGCNet model, a composite loss function, referred to as the target-oriented self-supervised loss function, is formulated, as presented in Eq. (20).

$$\begin{aligned} \mathcal{L=}\mathcal{L}_{G}+\alpha\mathcal{L}_{C}+\beta\mathcal{L}_{AR}+\gamma\mathcal{L}_{CVAE}+\sigma\mathcal{L}_{S}\#\left( 20 \right) \end{aligned}$$

#### **Calculation of coupling strength between organ system states**

The proposed model represented each patient’s organ functional state as a deep embedding vector. Cosine similarity was then used to quantify the similarity between the embedding vectors of different organ systems states. For two organ system states vectors $x$ and $y$, cosine similarity is defined as:

$$\begin{aligned} cosine\left( x,y \right)=\frac{x\cdot y}{\left\| x \right\|\left\| y \right\|}\#\left( 21 \right) \end{aligned}$$

where $x\cdot y$ denotes the dot product, $\left\| x \right\|$ and $\left\| y \right\|$ represent the Euclidean norms of the vectors.

Finally, for each patient or phenotype group, we averaged the pairwise cosine similarities across all organ systems to obtain the coupling strength, which reflected the overall consistency or coordination of multi-organ functional states.

### **1.2.2 Model implementation settings**

The MIMIC-III dataset was randomly divided into training, validation, and test sets in a 6:2:2 ratio, with the validation set used to optimize hyperparameter selection. The entire cohort from the eICU dataset served as an independent external validation set.

The model architecture was as follows: both the encoder and decoder of the conditional variational autoencoder (CVAE) were composed of three-layer neural networks. The encoder’s first two layers contained 64 and 32 neurons, respectively, followed by a variational output layer with a dimensionality of 3. The decoder received inputs of dimension 5, with subsequent hidden layers comprising 64 and 32 neurons, respectively. The $ReLU$ activation functions were applied to the first two layers of both the encoder and decoder. The adaptive information fusion layer incorporated a single-head attention mechanism with a dropout rate of 0.5 to reduce the risk of overfitting. The position-wise feed-forward network (FFN) consisted of two fully connected layers, each with 64 neurons. Residual connections and layer normalization were applied within the adaptive fusion module to prevent network degradation and stabilize activation distributions. The graph attention network module consisted of four attention layers with output dimensions of 64, 32, 16, and 3, respectively. The LSTM module was implemented as a single-layer network with an output dimension of 8. At each time step, the outcome prediction layer produced a two-dimensional output activated by a $Softmax$ function, while at the final time step, it generated a single output using a $Sigmoid$ activation function.

The network weights of the ODTGCNet model were initialized using the Xavier Uniform initialization strategy. The learning rate was set to 0.0003, with 1000 iterations and a batch size of 32. The model’s loss function, as shown in Eq. (20), was optimized using the error backpropagation algorithm, with the adaptive moment estimation (Adam) algorithm employed as the optimizer.

## **1.3 Model evaluation metrics**

### **1.3.1 Number of clusters determining**

We employed the Elbow Method^3^ to determine the optimal cluster number. The clustering objective was defined using the Sum of Squared Errors (SSE), as shown in Eq. (22). SSE quantifies the within-cluster dispersion by summing the squared distances between data points and their respective cluster centroids. A lower SSE indicates tighter, more cohesive clusters, whereas a higher SSE suggests looser internal structure. As the number of clusters increases, SSE typically decreases. However, for well-separated data, a sharp decline in SSE often occurs at a certain point, followed by a plateau. This inflection point can be interpreted as the optimal number of clusters.

$$\begin{aligned} SSE=\sum_{k,i} \left( x_{i,k}-\mu_{k} \right)^{2}\#\left( 22 \right) \end{aligned}$$

where $C_{i}$ denotes the $i$-th cluster, $x_{i}$ represents a data point in $C_{i}$, and $\mu_{i}$ is the centroid of $C_{i}$.

### **1.3.2 Silhouette Coefficient calculating**

The silhouette coefficient^4^ measures the similarity between a sample and other samples within the same cluster, as well as the dissimilarity to samples in different clusters, providing a quantitative assessment of clustering quality. A higher SC value indicates better clustering performance.

$$\begin{aligned} s_{i}=\frac{b_{i}-a_{i}}{\max\{a_{i},b_{i}\}}\#\left( 23 \right) \end{aligned}$$

For a given sample $i$, the average distance to other samples within the same cluster is calculated as $a_{i}$, while the average distance to all samples in any other cluster $j$ is denoted as $b_{ij}$. The silhouette coefficient is defined as the minimum value of $b_{ij}$. The silhouette coefficient ranges from -1 to 1. A coefficient close to 1 indicates that the samples within a cluster are tightly grouped, while samples from different clusters are well-separated, signifying optimal clustering. A negative silhouette coefficient suggests poor clustering performance.

### **1.3.3 Davies-Bouldin index calculating**

The Davies-Bouldin Index^5^ evaluates clustering quality from another perspective by calculating the ratio of intra-cluster compactness to inter-cluster separation. A lower DBI value indicates better clustering performance. The DB index is calculated according to the Eq. (24).

$$\begin{aligned} DB=\frac{1}{n}\sum_{i=1}^{n} \max_{j\neq i} \left( \frac{\sigma_{i}+\sigma_{j}}{d\left( C_{i},C_{j} \right)} \right)\#\left( 24 \right) \end{aligned}$$

where $\sigma_{i}$ represents the average distance from the cluster center to all its sample points, and $d\left( C_{i},C_{j} \right)$ denotes the inter-cluster distance between samples.

## **1.4 Experiments design**

### **1.4.1 Baseline models settings**

To evaluate the effectiveness of the proposed method, state-of-the-art (SOTA) models were selected as baseline models for comparison. Specifically, the chosen baseline models include:

- DEC^6^: A deep clustering algorithm that employs an autoencoder (AE) to obtain low-dimensional feature representations. It defines a centroid-based probability distribution and minimizes the KL divergence between this distribution and an auxiliary target distribution to jointly optimize clustering assignments and feature representation quality.
- IDEC^7^: An improved deep clustering algorithm that extends DEC by incorporating a decoder and optimizing both the reconstruction error and clustering loss function to enhance clustering performance.
- DAEGC^8^: A deep graph clustering algorithm that leverages an attention mechanism to identify the importance of neighboring nodes for the target node. It integrates graph topology and node features into a compact representation while using an inner product decoder to reconstruct the graph structure.
- SDCN^9^: A deep graph clustering algorithm that introduces a transition operator to propagate the representations learned by the autoencoder to corresponding GCN layers. This model employs a dual self-supervised mechanism to guide model parameter updates.

### **1.4.2 Ablation experiments settings**

To systematically evaluate the contribution and effectiveness of each component in the ODTGCNet model, a series of ablation experiments were designed as follows:

- ODTGCNet_VAE_: Replace the CVAE module with a VAE module.
- ODTGCNet_AE_: Replace the CVAE module with an AE module.
- ODTGCNet_Att-_: Replace the adaptive information fusion layer with a fixed information fusion layer.
- ODTGCNet_GCN_: Replace the GAT unit with a GCN unit.
- ODTGCNet$\mathcal{L}_{S}$_-_: Set $\mathcal{L}_{S}=0$ in the loss function.
- ODTGCNet$\mathcal{L}_{AR}$_-_: Set $\mathcal{L}_{AR}=0$ in the loss function.
- ODTGCNet$\mathcal{L}_{C}$_-_: Set $\mathcal{L}_{C}=0$ in the loss function.

## **1.5 Web interface construction**

The web-based interface for sepsis clustering in this study was developed using Streamlit (https://streamlit.io/), a lightweight Python framework designed for rapid prototyping in data science applications. The interface comprises four main components:

- A login page enabling user authentication and access control;
- A navigation and data input panel with a sidebar and standardized upload functionality, supporting structured patient phenotype data in formats such as CSV or Excel;
- A model execution interface that allows users to initiate backend clustering algorithms via button-triggered actions, with real-time status updates;
- A results page displaying clustering performance metrics and patient subtyping outcomes through interactive charts and tables (native Streamlit components).

The layout utilizes “st.sidebar” for navigation and “st.tabs” for organizing content on the main page. Upon data upload, the system performs automated validation and preprocessing, followed by clustering execution upon user confirmation. The results are cached and rendered through interactive components for further exploration.

## **1.6 Intravenous fluid analysis across phenotypes**

This study extracted fluid input data from both Metavision (inputevents_mv) and CareVue (inputevents_cv) systems in the MIMIC-III database. We included commonly used clinical fluids, covering a wide range of types: crystalloids (e.g., normal saline, various dextrose solutions), colloids (e.g., albumin), hypertonic saline (3%, 7.5%, 23.4% NaCl), osmotic agents (e.g., mannitol), and alkalinizing agents (e.g., sodium bicarbonate).

To accurately reflect the effective intravascular volume contribution of different fluids, we applied a tonicity-adjusted conversion factor to standardize all input volumes into tonicity-equivalent volumes (TEV), thereby minimizing confounding due to fluid composition differences. For each record, we calculated TEV based on fluid type and tonicity coefficient, and then aggregated these across all records for each patient. Volumes were further normalized by body weight and stratified within defined time windows (e.g., 0-12h, 12-24h) for statistical analysis. Standardized fluid volumes were expressed in mL/kg and categorized into three clinically relevant dosing strata: <30, 30-50, and >50 mL/kg. Due to inherent data limitations and the scope of this study, we did not further differentiate the individual therapeutic effects of specific fluid categories across phenotypes — e.g., differential responses to crystalloids versus colloids — or assess interaction effects with co-administered treatments such as vasopressors.

Building upon existing studies, the definition and calculation of fluid input data in this study are detailed as follows:

**(1) Data extraction criteria**

Intravenous fluid input volumes for ICU patients were extracted from the MIMIC-III database during their hospital stay. Since MIMIC-III includes two distinct clinical information systems —CareVue and MetaVision — we separately extracted fluid input events from the corresponding tables: inputevents_cv (CareVue) and inputevents_mv (MetaVision), and subsequently merged the datasets for integrated analysis.

**(2) Fluid selection and Item ID scope**

We selected fluid records corresponding to clinically common resuscitation and therapeutic fluids based on specific item ids representing different fluid categories (see Supplementary Table S18):

| **Fluid category** | **Sample Item IDs** | **Representative fluids** |
| --- | --- | --- |
| Crystalloids | 30054, 30055, 30101, 30102, 30103, 30104, 30105, 30108, 226361, 226363 | Normal saline (0.9% NaCl), Dextrose solutions (e.g., D5W), etc. |
| Colloids | 226364, 226365, 226367, 226368, 226369, 226370, 226371, 226372 | Albumin (5% or 25%), etc. |
| Hypertonics | 226375, 226376, 227070, 227071, 227072 | Hypertonic saline |

**(3) Fluid type and tonicity correction**

Different fluids vary in osmotic and colloid osmotic pressures, resulting in differing vascular volume expansion effects. To better reflect the effective circulating volume contributed by each fluid, we applied tonicity correction as follows: The administered volume for each fluid itemid was multiplied by a corresponding correction factor to compute the Total Equivalent Volume (TEV). For example, isotonic saline was assigned a factor of 1, hypertonic saline 0.25, and colloids 3, as defined in prior literature and clinical experience. This correction aims to more accurately represent the vascular expansion effects of diverse fluids.

**(4) Data processing workflow**

- Data extraction: Real-time fluid input data from MetaVision (inputevents_mv) include start and stop times, volumes, and infusion rates; CareVue data (inputevents_cv) lack infusion rates and are treated as STAT (bolus) administrations.
- Fluid filtering: Only records within the specified itemid ranges were retained; invalid, zero-volume, or non-therapeutic fluids were excluded.
- Volume calculation: For each record, TEV was calculated by multiplying input volume with the fluid-specific correction factor; totals were aggregated per patient by fluid type over the ICU stay.
- Data integration: Fluid input totals from MetaVision and CareVue were merged via icustay_id, prioritizing MetaVision data when available.
- Normalization and time windowing: Volumes were normalized by patient body weight and stratified into clinically relevant time windows (e.g., first 12 hours) for statistical analyses.

## **1.7 The impact of ICU type on phenotype-specific fluid strategies**

To further evaluate whether ICU type modifies the effect of fluid strategies, we constructed a logistic regression model including an interaction term between fluid strategy and ICU type: C(fluid_strategy) × C(ICU_type). We compared this interaction model to a reduced model without the interaction term using a likelihood ratio test.

As shown in Supplementary Table S12, a significant interaction effect was observed for Phenotype A within the first 12 hours, suggesting that some postoperative cardiac surgery patients may be included in this group, potentially confounding the phenotype composition and fluid strategy conclusions. No significant interaction was detected for Phenotypes B and C.

Accordingly, for patients in Phenotype A, we conducted a sensitivity analysis excluding CSRU patients and found that, after their removal, the interaction between ICU type and phenotype was no longer statistically significant.

Supplementary Fig. S10 E and F depict the odds ratios of fluid strategies within 0-12 hours across different phenotypes, respectively before and after excluding CSRU patients. We observed consistent OR trends between the two cohorts. Notably, for Phenotype A patients, strategies 1 and 2 both show a slight reduction in OR compared to strategy 0.

We performed a sensitivity analysis limited to medical ICU (MICU) patients. Within each phenotype, we examined the association between fluid input volumes during 0-12 and 12-24 hours and mortality to assess the stability of fluid strategy applicability in a more homogeneous patient population. As shown in Supplementary Fig. S14, the response trends to fluid strategies across phenotypes remain largely consistent, supporting the clinical stratification potential of the identified phenotypes.

## **1.8 Relationships between temporal risk stratifications and mortality**

To enable dynamic labeling of each patient’s risk status, phenotype classification was performed at the 48-hour mark, while risk stratification was generated at fixed 4-hour intervals throughout the observation window.

As illustrated in Supplementary Fig. S4 and Supplementary Fig. S5, Phenotype B exhibited the highest transition rate from risk level 0 to levels 1 or 2, compared to Phenotype A. It elevated transition rate was accompanied by an increased mortality, indicating a higher susceptibility to clinical deterioration in this group. Notably, in Phenotype B, patients who initially presented at risk level 2 maintained persistently high mortality, irrespective of subsequent transitions in risk level. This finding underscores the need for heightened surveillance and early intervention in patients with a high initial risk within this phenotype.

In Phenotype C, the trajectory of risk level worsening was even more marked. This group had the largest proportion of patients who remained at risk level 2, which was associated with the highest observed mortality across all phenotypes - further reinforcing the strong correlation between sustained high-risk status and adverse outcomes. Moreover, even among patients in Phenotype C who demonstrated apparent clinical improvement (e.g., those following a 1–x–0 trajectory), mortality remained higher than in Phenotype A patients who experienced deterioration (e.g., 1–x–2 trajectory), highlighting the persistent poor prognosis associated with Phenotype C.

## **1.9 Phenotype variation across different ICU types in sepsis patients**

To assess whether the identified phenotypes represent underlying pathophysiological differences independent of ICU type, we designed the following analyses.

**(1) Phenotype distribution and mortality stratification across ICU types**

As shown in Supplementary Fig. S9 A, we performed a stratified analysis of phenotype distribution and in-hospital mortality across different ICU types. The results demonstrate that:

- All three phenotypes are represented across all ICU types, indicating that the clustering results are not driven by any single unit;
- Within each ICU type, a consistent mortality gradient is observed — Phenotype A shows the lowest mortality, while Phenotype C exhibits the highest;
- These findings suggest that the identified phenotypes reflect not just static admission characteristics, but rather risk trajectories shaped by dynamic multi-organ interactions over time.

**(2) Phenotypes vs. ICU types: predictive power for mortality**

To further assess whether the identified phenotypes provide prognostic value beyond ICU type, we constructed two separate univariate logistic regression models to predict in-hospital mortality, as shown in Supplementary Fig. S9 B and C:

- Using ICU type alone as the predictor yielded an AUROC of 0.56 (95% CI: [0.53, 0.59]);
- Using phenotype alone as the predictor yielded a significantly higher AUROC of 0.74 (95% CI: [0.71, 0.77]);
- Additional performance metrics including specificity, F1 score, and precision also consistently favored the phenotype-based model.

**(3) Phenotype-specific organ coupling patterns are consistent across ICU types**

We further examined the temporal dynamics of organ system coupling strength across phenotypes within different ICU types. The results reveal that:

- Across all ICU types, the three phenotypes consistently exhibit distinct patterns of inter-organ coordination:
  - Phenotype A: sustained synchronous recovery;
  - Phenotype B: persistent asynchronous dysregulation;
  - Phenotype C: catastrophic evolution from asynchrony to coordinated failure.
- These trends are statistically significant (P $<$ 0.05) and robustly observed in CSRU, MICU, and the overall ICU population.
- Thus, regardless of admission context — such as postoperative cardiac care or trauma—the dynamic evolution of inter-organ coordination, rather than ICU type or clinical background, serves as the primary determinant of phenotype classification.

**(4) Phenotype structure remains robust after excluding CSRU patients**

To further exclude the potential confounding effects of postoperative patients from the CSRU, we conducted a comprehensive sensitivity analysis, as shown in Supplementary Fig. S10:

- Mortality distribution remained largely unchanged: Supplementary Fig. S10 A and B show the patient distribution and in-hospital mortality rates of the three phenotypes before and after excluding CSRU patients. Phenotype A consistently represents a low-mortality, large-sized group (5.7% vs. 8.0%), while Phenotype C remains a high-mortality, small-sized group (38.3% vs. 39.2%). The gradient of mortality risk across phenotypes persisted after CSRU exclusion, suggesting good stability of the phenotype classification.
- Organ coupling patterns were minimally affected (Supplementary Fig. S10 C and D): We further compared the strength of inter-organ interactions, particularly focusing on the circulatory system (which did not differ significantly across phenotypes). The coupling patterns showed minimal changes post-CSRU exclusion. For instance, Phenotype A still exhibited a gradual increase in coupling strength, Phenotype B maintained a persistent decrease, and Phenotype C continued to show a pattern of early desynchronization followed by late-phase collapse over the 48-hour window.

**(5) Differences in baseline population characteristics among the three phenotypes**

Baseline characteristics of the three phenotypes stratified by ICU type are summarized in Supplementary Tables S5-S9 to distinguish phenotype-specific differences from ICU type-related variation.

**① Significant and consistent differences in population structure among the three phenotypes**

We conducted statistical analyses of baseline patient characteristics across all ICU types and observed a consistent gradient trend from Phenotype A to C in demographics and comorbidity burden, including:

- Increasing age: Phenotype C patients were significantly older across ICU types—for example, in CSRU, mean age was 68.7 vs. 75.1 years (A vs. C, P < 0.05); in MICU, 60.1 vs. 68.2 years (P < 0.05).
- Decreasing body weight: Mean weight declined progressively from A to C, suggesting worsening nutritional status or higher chronic disease burden.
- Rising comorbidity burden: Incidence of renal and respiratory diseases increased—for instance, renal disease prevalence in MICU was 56.1% in A versus 77.1% in C (P < 0.05); in CSRU, 30.0% vs. 53.9% (P < 0.05).
- Increasing mortality: Phenotype A consistently showed the lowest mortality, while Phenotype C exhibited significantly higher mortality across all ICU types.

**②** **Significant differences in ICU and hospital length of stay were observed only in CSRU and SICU, but not in other units.**

Further analysis of ICU and hospital stays revealed:

- In CSRU and SICU, phenotype C was associated with significantly prolonged ICU and hospital stays (e.g., in CSRU, ICU stay: phenotype A = 4.1 days vs. C = 13.2 days, P < 0.05);
- In contrast, no significant differences were observed in MICU and TSICU (e.g., in MICU, ICU stay: A–C = 6.4–7.3 days, P= 0.125).

## **1.10 Model comprehensive clustering performance comparison**

As shown in Supplementary Fig. S10 and Supplementary Table S5, on the MIMIC-III dataset, ODTGCNet improved the SC score by 0.23-0.16 and reduced the DBI score by 0.55-0.37 compared to existing models. On the eICU dataset, ODTGCNet achieved an SC score improvement of 0.24-0.06 and a DBI reduction of 0.59-0.29 over the baseline models. Notably, the deep graph clustering models DAEGC and SDCN demonstrated superior performance compared to the DEC and IDEC models, with SDCN ranking second only to ODTGCNet in overall performance.

According to Supplementary Table S6, ODTGCNet consistently outperformed other baseline models in temporal risk stratification performance across all evaluated time windows. The temporal risk stratification performance of the models at different time points is illustrated in Supplementary Fig. S10. Notably, during the 4-hour time window following sepsis diagnosis, the clustering performance of the ODTGCNet model was relatively limited. However, as the observation window extended, the model exhibited a clear improvement or maintained stable clustering performance. At 48 hours, the ODTGCNet model achieved its optimal clustering performance.

As shown in Supplementary Tables S7, to validate the effectiveness of the conditional variational autoencoder and the embedding of prognostic outcomes, the ODTGCNet model was compared with the ODTGCNet_VAE_ model. The results indicated that ODTGCNet achieved an improvement of 0.07 in SC score and a reduction of 0.11 in DBI score on both the MIMIC-III and eICU datasets. Compared with the ODTGCNet_AE_ model, the SC score improved by 0.10 and 0.08, while the DBI score decreased by 0.16 and 0.12 on the MIMIC-III and eICU datasets, respectively. To verify the effectiveness of the adaptive information fusion layer, the ODTGCNet was compared with ODTGCNet_Att-_. It showed that the SC score increased by 0.24 and 0.17, and the DBI score improved by 0.48 and 0.35 on the MIMIC-III and eICU datasets, respectively. To assess the impact of the GAT module, we compared ODTGCNet with ODTGCNet_GCN_. The ODTGCNet model achieved improvements of 0.07 and 0.11 in SC score and reductions of 0.14 and 0.22 in DBI score on the MIMIC-III and eICU datasets, respectively. Among all ablated models, ODTGCNet_Att-_ exhibited the weakest performance. Additionally, it is noteworthy that the ODTGCNet_VAE_ model outperformed the ODTGCNetAE model, further demonstrating the effectiveness of the variational autoencoder.

To investigate the impact of loss function components on the model’s clustering performance, a series of ablation experiments on the loss function were conducted. As shown in Supplementary Tables S7, compared with the complete loss function, the ODTGCNet$\mathcal{L}_{S}$_-_ model suffered the most significant degradation in clustering performance, with SC scores decreasing by 0.18 and 0.24 and DBI scores worsening by 0.33 and 0.52 on the MIMIC-III and eICU datasets, respectively. In contrast, the performance deterioration of the ODTGCNet$\mathcal{L}_{AR}$_-_ model was less pronounced; on the MIMIC-III dataset, both the SC and DBI scores remained unchanged, while on the eICU dataset the SC score decreased by only 0.02 and the DBI score worsened by 0.04.

As illustrated in Supplementary Fig. S14 and S15, a series of comparative experiments were conducted to evaluate the specific impact of loss function coefficients on the model’s clustering performance. The model achieved optimal clustering performance on both the MIMIC-III and eICU datasets when the $\sigma$ coefficient was set to 0.03 and the $\beta$ coefficient to 0.001. In addition, further optimization of the other loss function coefficients yielded an $\alpha$ coefficient of 10 and a $\gamma$ coefficient of 0.05, which further enhanced the model’s clustering performance.

1.11 Construction of a simple phenotype classifier

The XGBoost-based classifier achieved early phenotypes stratification by integrating time-varying clinical features. The temporal dynamics of feature importance revealed shifting inter-organ interaction hubs, offering a foundation for real-time bedside decision support. In Phenotype A, the growing importance of BUN and PTT at 48 hours indicated increasing reliance on organ function markers for late-stage risk prediction. In Phenotype B, early risk was driven by respiratory and infection-related variables, while later predictions were more sensitive to renal and coagulation dysfunction. For Phenotype C, late-stage risk assessment was predominantly associated with markers of inflammation and metabolic dysregulation. Overall, early (4-hour) predictions across all phenotypes depended primarily on vital signs and baseline scores, whereas later (48-hour) predictions were dominated by laboratory and organ-specific indicators, reflecting a dynamic shift in predictive features over time. Notably, phenotypic heterogeneity was evident in feature contributions: PaO_2_/FiO_2_ was a strong predictor in Phenotype B but not in A or C.

# **2. Supplementary Figures**

## **Supplementary Fig. S1**


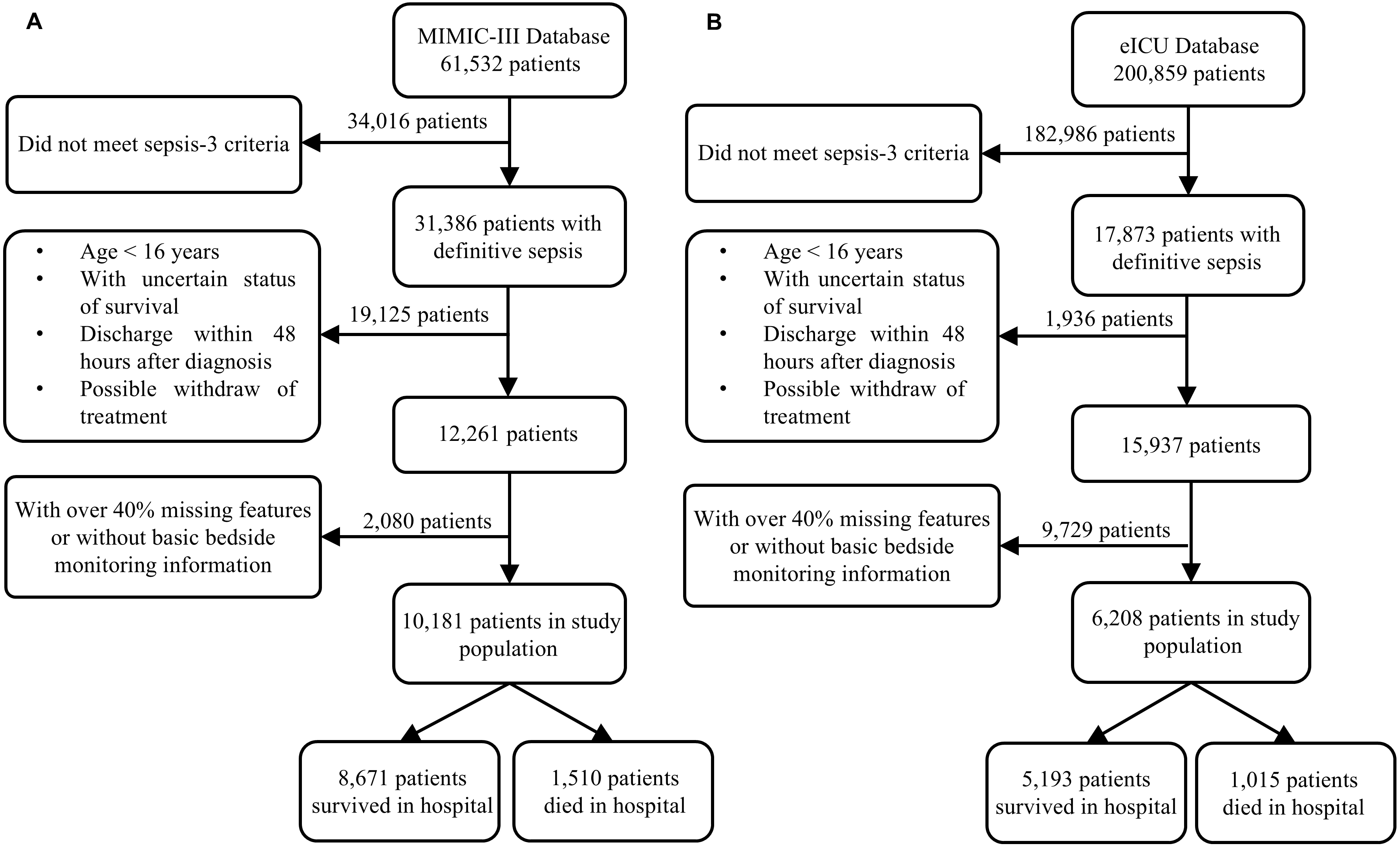


Supplementary Fig. S1. Patient cohort selection flowchart. (A) Selection process for the MIMIC-III patient cohort. (B) Selection process for the eICU patient cohort.

## **Supplementary Fig. S2**


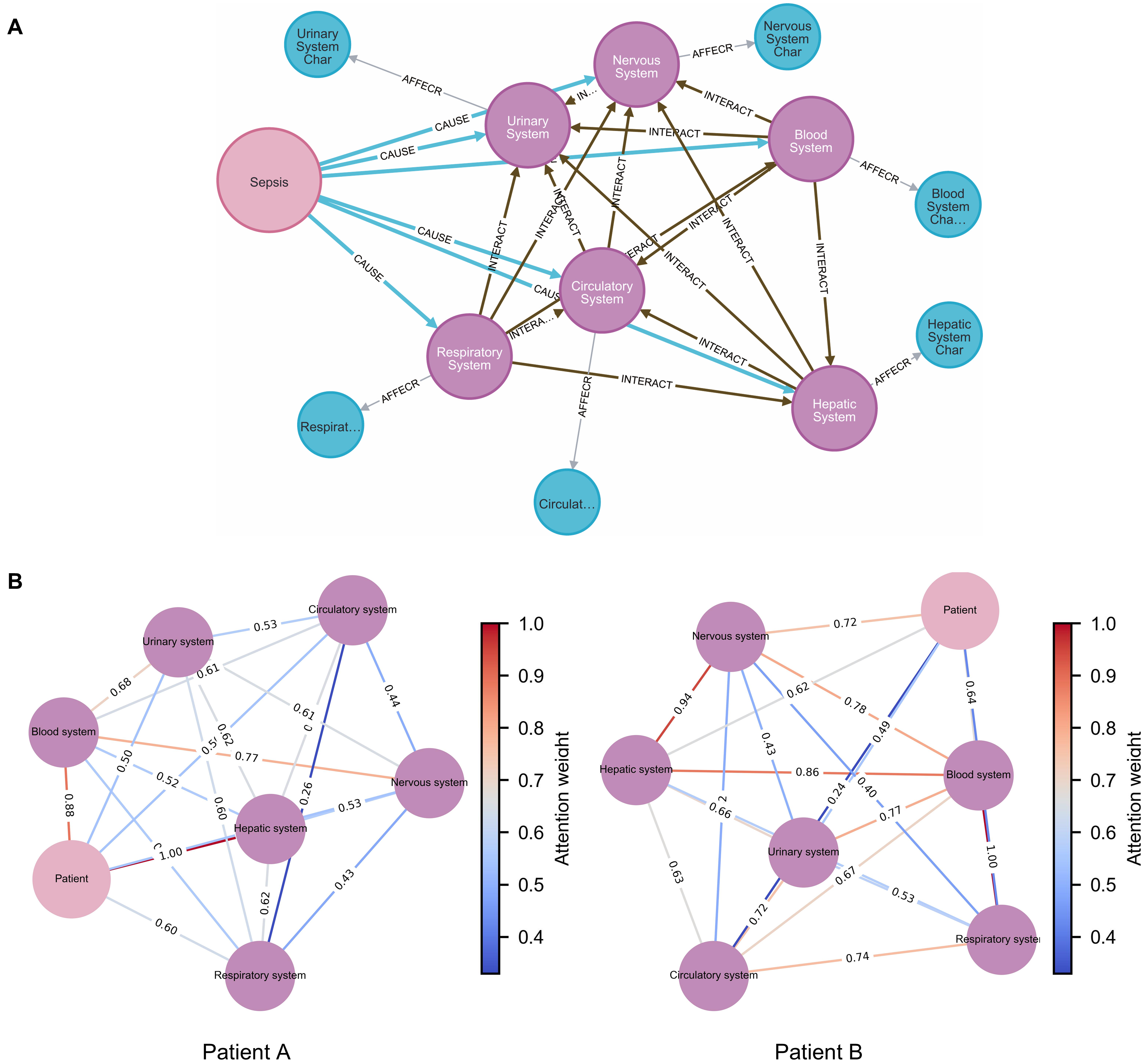


Supplementary Fig. S2. Sepsis organ interaction graph structure. (A) Sepsis organ interaction graph. The nodes in the graph include patient nodes, physiological system nodes, and physiological characteristic nodes. The physiological system nodes encompass the circulatory system, urinary system, blood system, hepatic system, respiratory system, and nervous system. (B) Adaptive graph edge weights. The edge weights shown in the diagram are dynamically assigned according to attention scores learned by the model. These attention-based weights capture the varying strengths of relationships between nodes and enable the graph to adapt to underlying data patterns.

## **Supplementary Fig. S3**


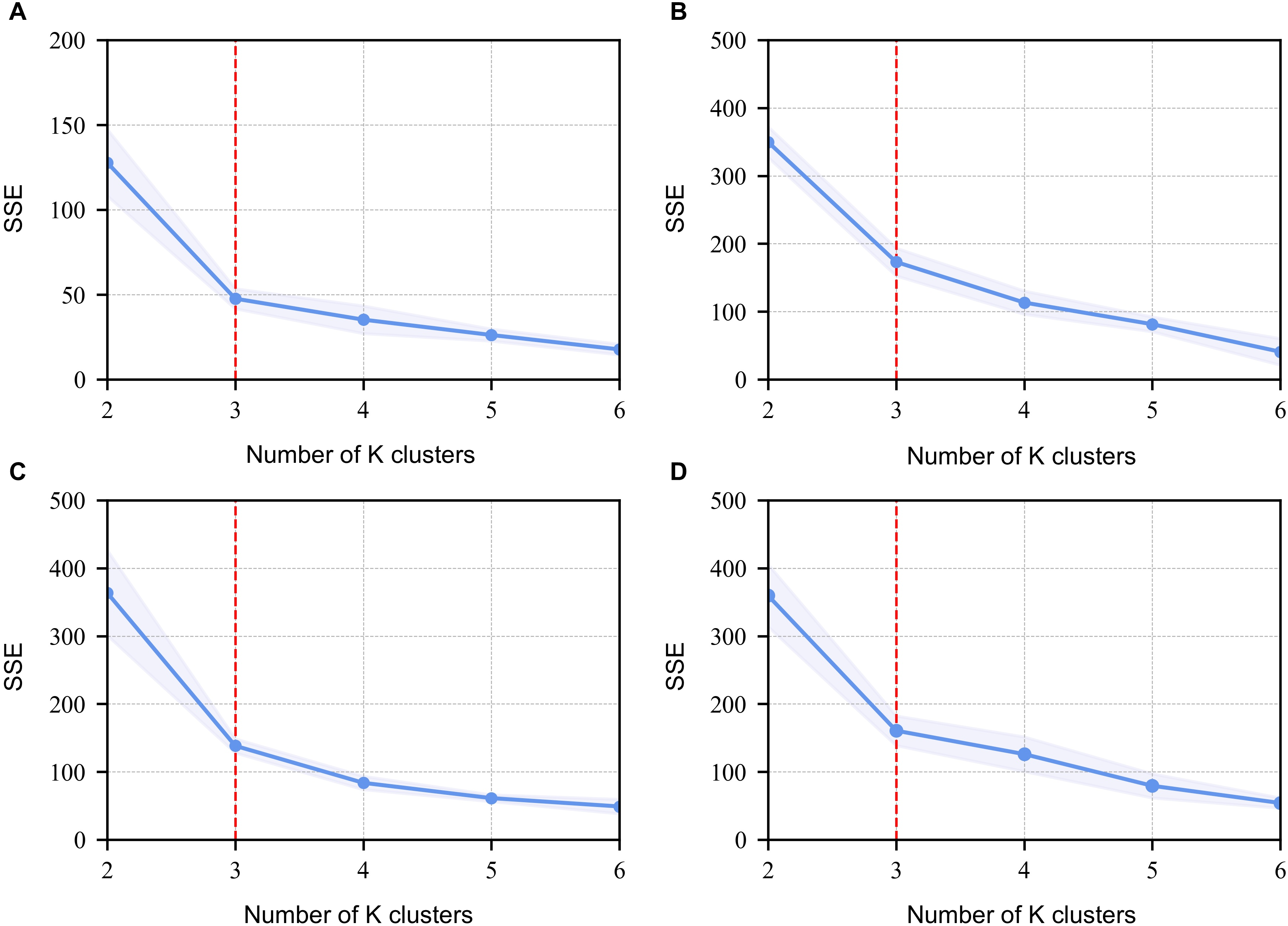


Supplementary Fig. S3. SSE curves with varying cluster numbers K. (A) 4 hours after sepsis diagnosis; (B) 16 hours after sepsis diagnosis; (C) 32 hours after sepsis diagnosis; (D) 48 hours after sepsis diagnosis.

## **Supplementary Fig. S4**


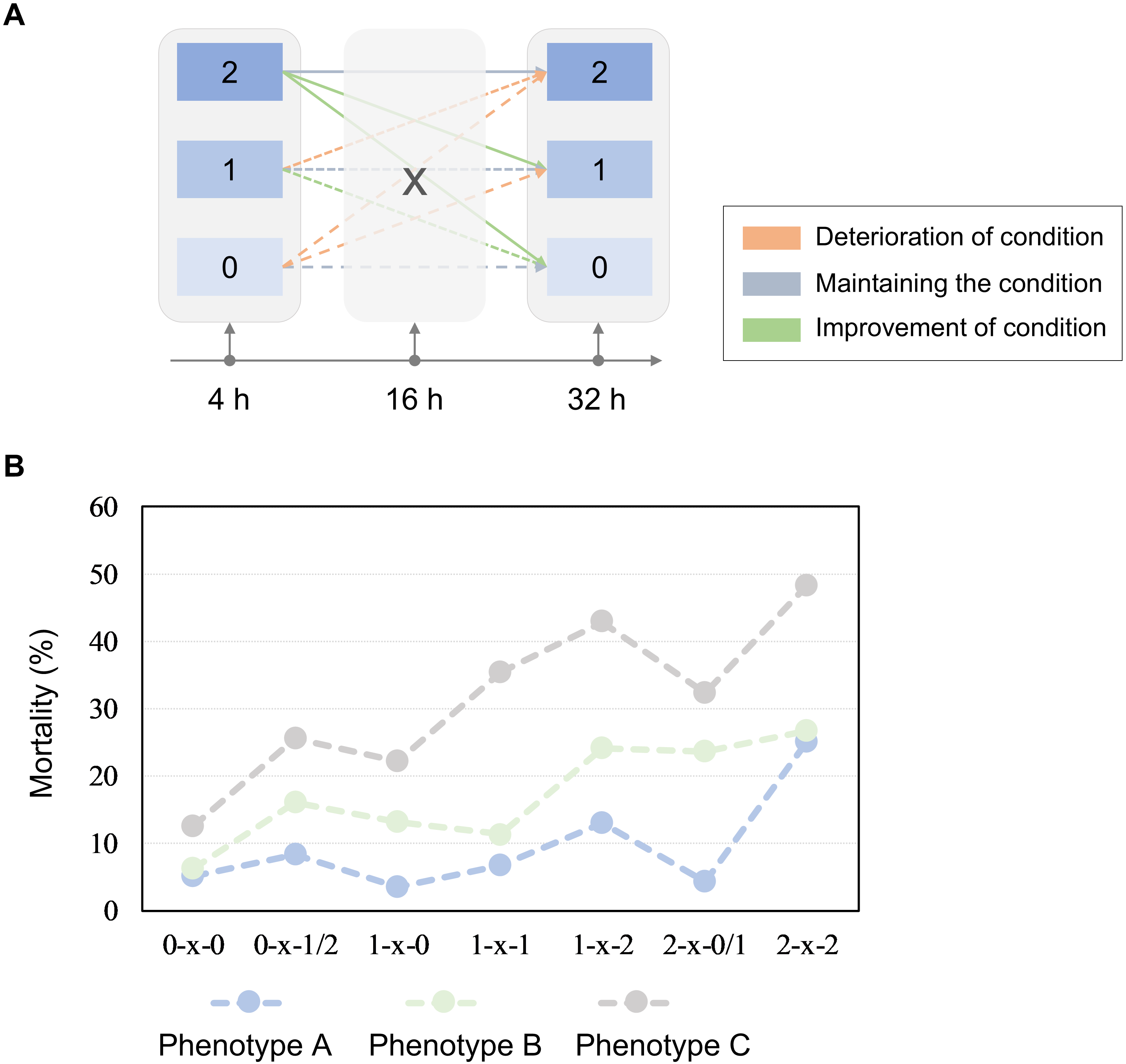


Supplementary Fig. S4. Temporal risk stratification distribution and mortality across sepsis phenotypes. (A) Temporal risk stratification transition patterns; (B) Relationship between different temporal risk stratification transition patterns and mortality. The x-axis represents risk stratification transition patterns, and the y-axis represents mortality rates.

## **Supplementary Fig. S5**


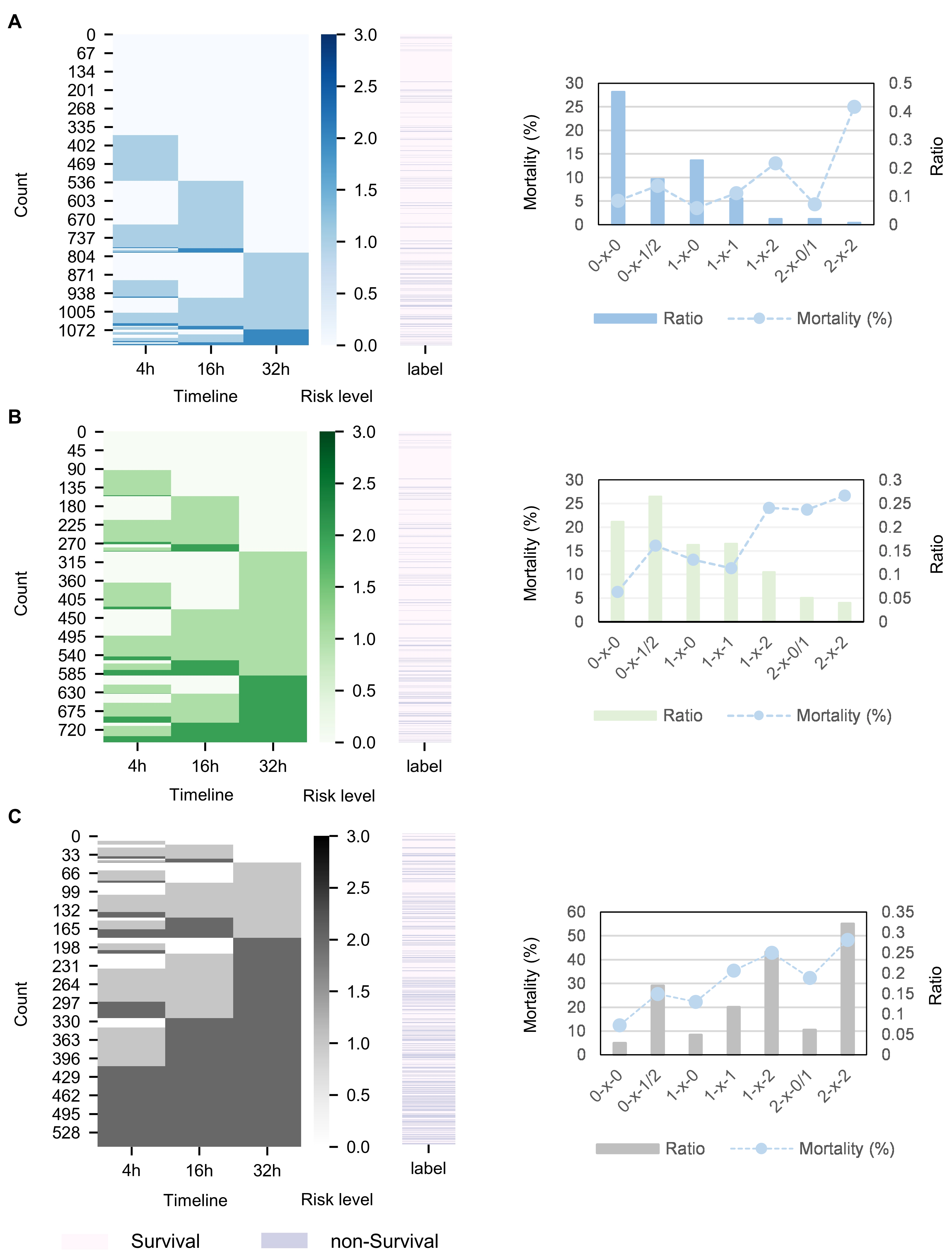


Supplementary Fig. S5. Relationship between temporal risk stratification and mortality across sepsis phenotypes in the MIMIC-III test cohort. (A) Phenotype A; (B) Phenotype B; (C) Phenotype C. In the heatmap, the *x*-axis represents time points, and the *y*-axis represents the number of patients. The color intensity indicates different risk levels, with darker colors representing higher risk levels. The two-color bar on the right side of the heatmap denotes patient outcomes. In the line-bar chart, the *x*-axis represents different risk stratification transition patterns, the left *y*-axis indicates mortality, and the right *y*-axis represents the proportion of patients.

## **Supplementary Fig. S6**


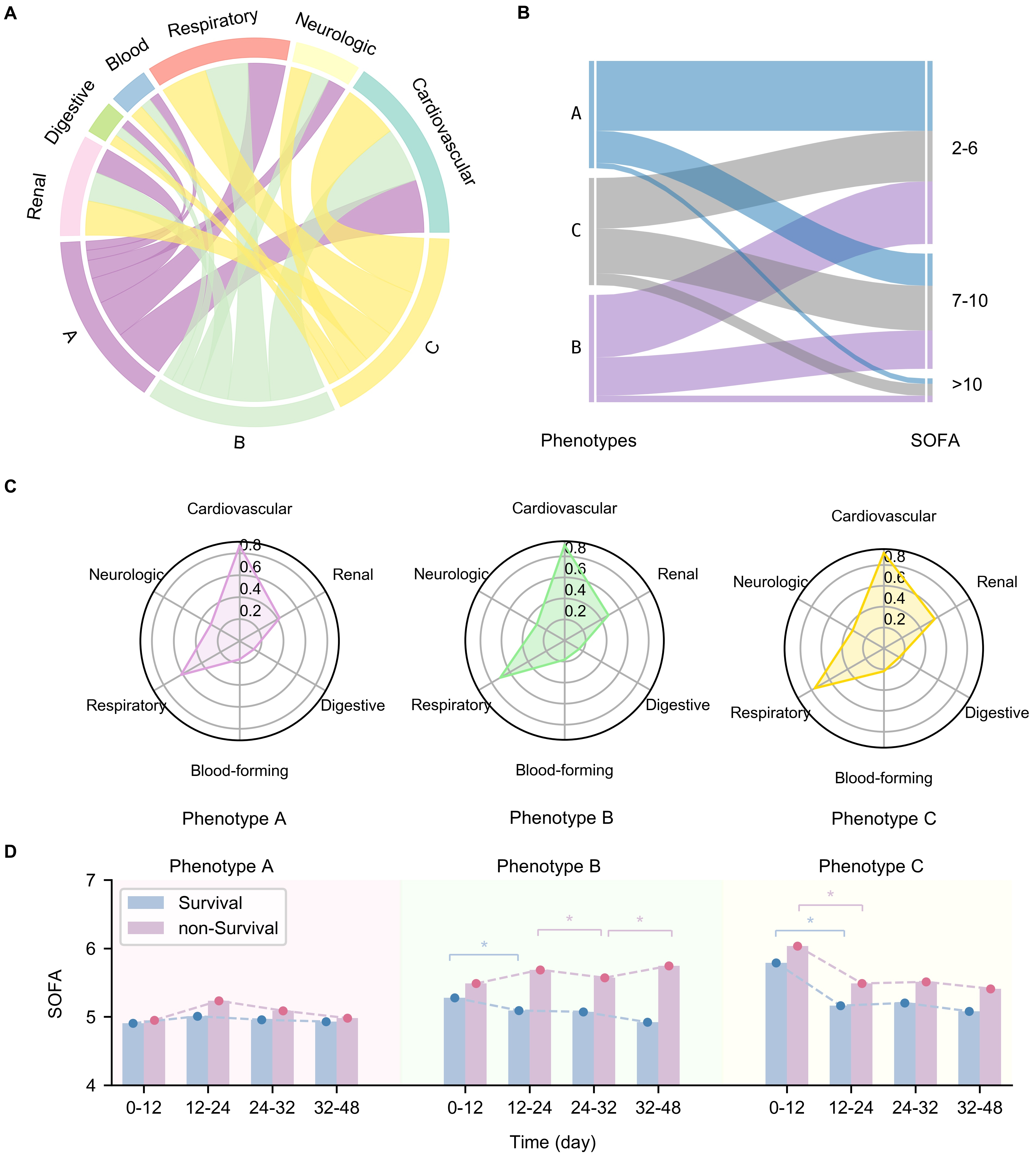


Supplementary Fig. S6. Comparison of organ dysfunction across sepsis phenotypes in the eICU cohort. (A) Chord diagram of comorbidities across phenotypes. This diagram visualizes the distribution of organ system dysfunctions across different phenotypes. The affected organ systems include the renal, gastrointestinal, hematologic and hematopoietic, respiratory, neurological, and circulatory systems. The bandwidth represents the proportion of patients with a specific organ dysfunction within each phenotype. (B) Alluvial plot of SOFA score distribution among phenotypes. The left column represents sepsis phenotypes, while the right column categorizes SOFA scores into three levels. Each phenotype is color-coded, and the alluvial plot illustrates the relationship between different sepsis phenotypes and SOFA score distributions. (C) Radar chart of comorbidities across phenotypes. This radar chart depicts the prevalence of comorbidities among different phenotypes, using the same organ systems as shown in (A). (D) Temporal trends of SOFA scores. The *x*-axis represents time, while the *y*-axis indicates SOFA scores. The curves depict changes in SOFA scores over time, with different colors representing different phenotypes. *P < 0.05 indicates statistically significant differences.

## **Supplementary Fig. S7**


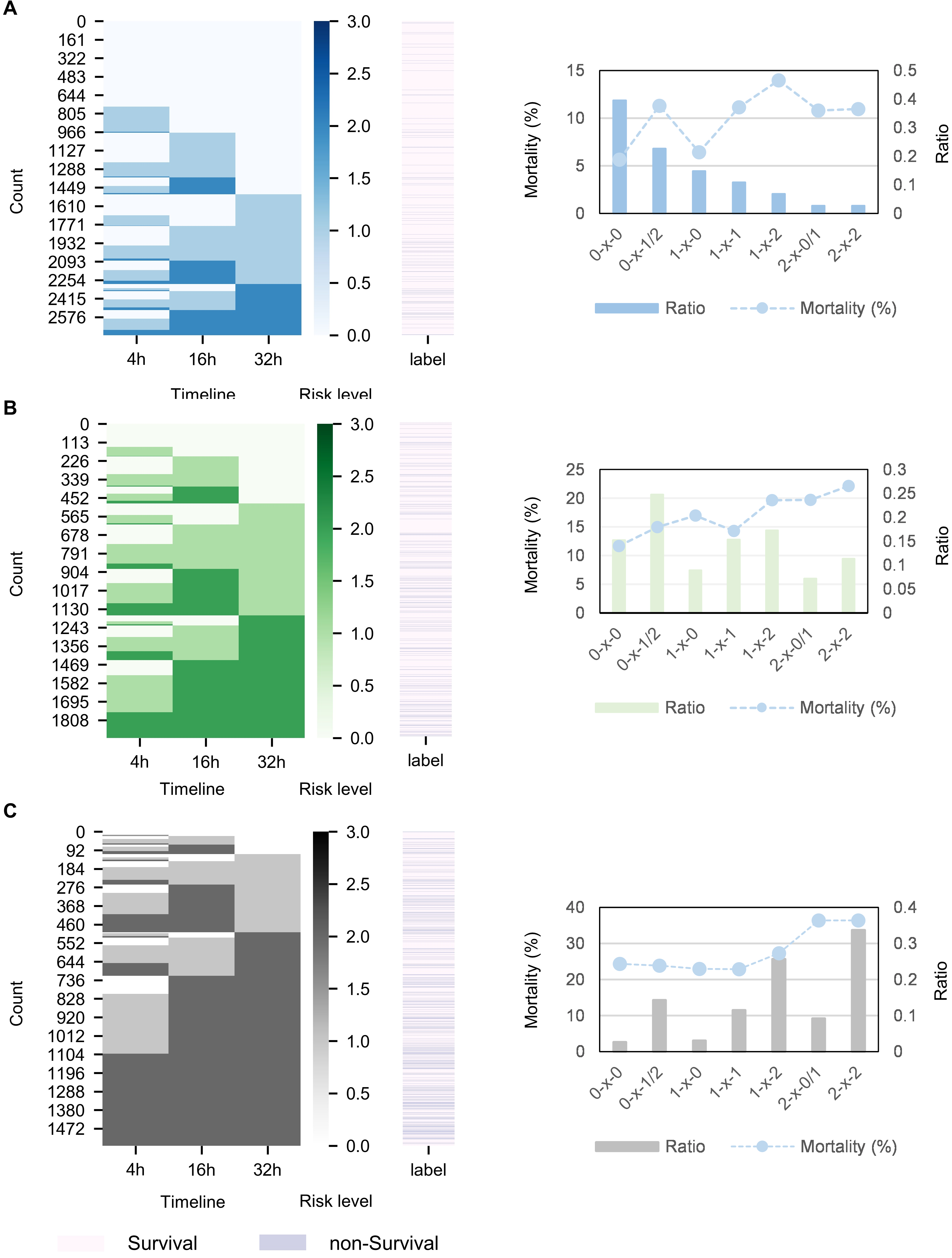


Supplementary Fig. S7. Relationship between temporal risk stratification and mortality across sepsis phenotypes in the eICU cohort. (A) Phenotype A; (B) Phenotype B; (C) Phenotype C. In the heatmap, the *x*-axis represents time points, and the *y*-axis represents the number of patients. The color intensity indicates different risk levels, with darker colors representing higher risk levels. The two-color bar on the right side of the heatmap denotes patient outcomes. In the line-bar chart, the *x*-axis represents different risk stratification transition patterns, the left *y*-axis indicates mortality, and the right *y*-axis represents the proportion of patients.

## **Supplementary Fig. S8**


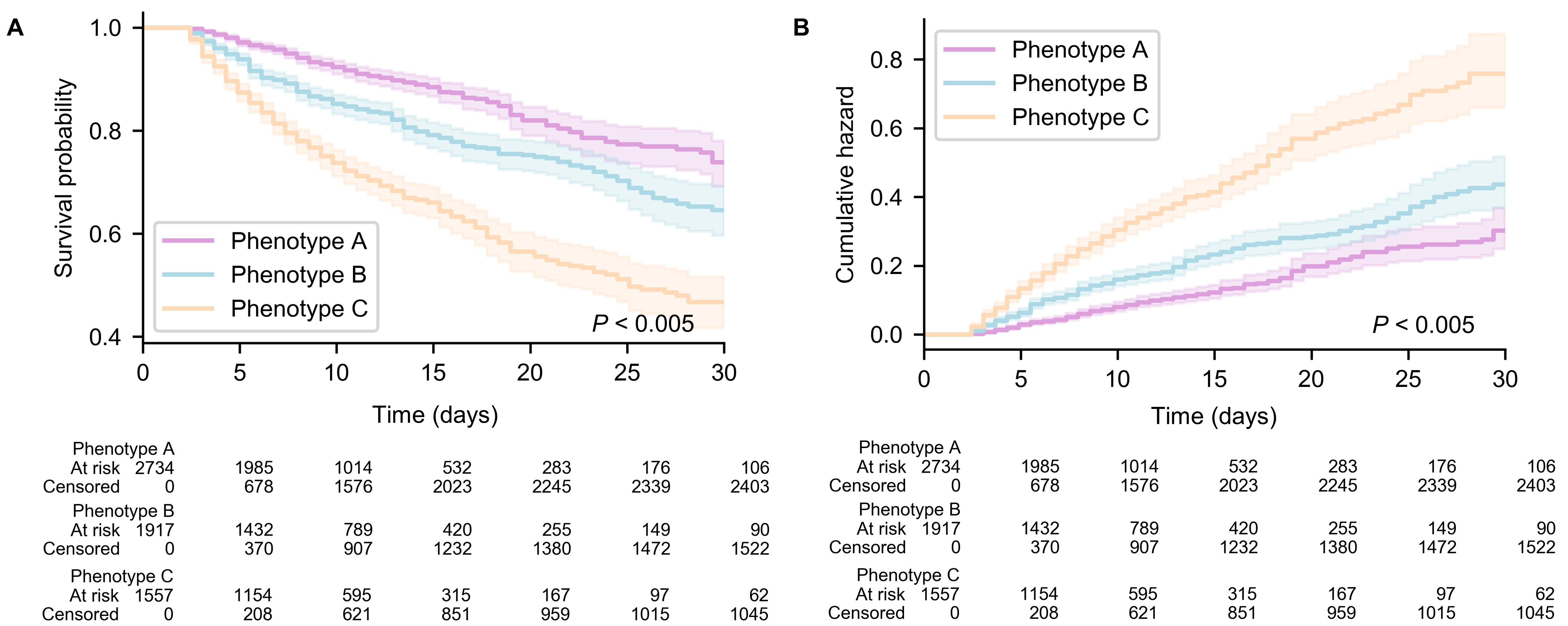


Supplementary Fig. S8. Survival analysis curves for different sepsis phenotypes in eICU dataset. (A) Kaplan-Meier survival curve: The *x*-axis represents the time since sepsis diagnosis, and the *y*-axis represents the survival probability. (B) Kaplan-Meier cumulative risk curve. The *x*-axis represents the time since sepsis diagnosis, and the *y*-axis represents the cumulative risk. *P < 0.005 indicates statistically significant differences.

## **Supplementary Fig. S9**


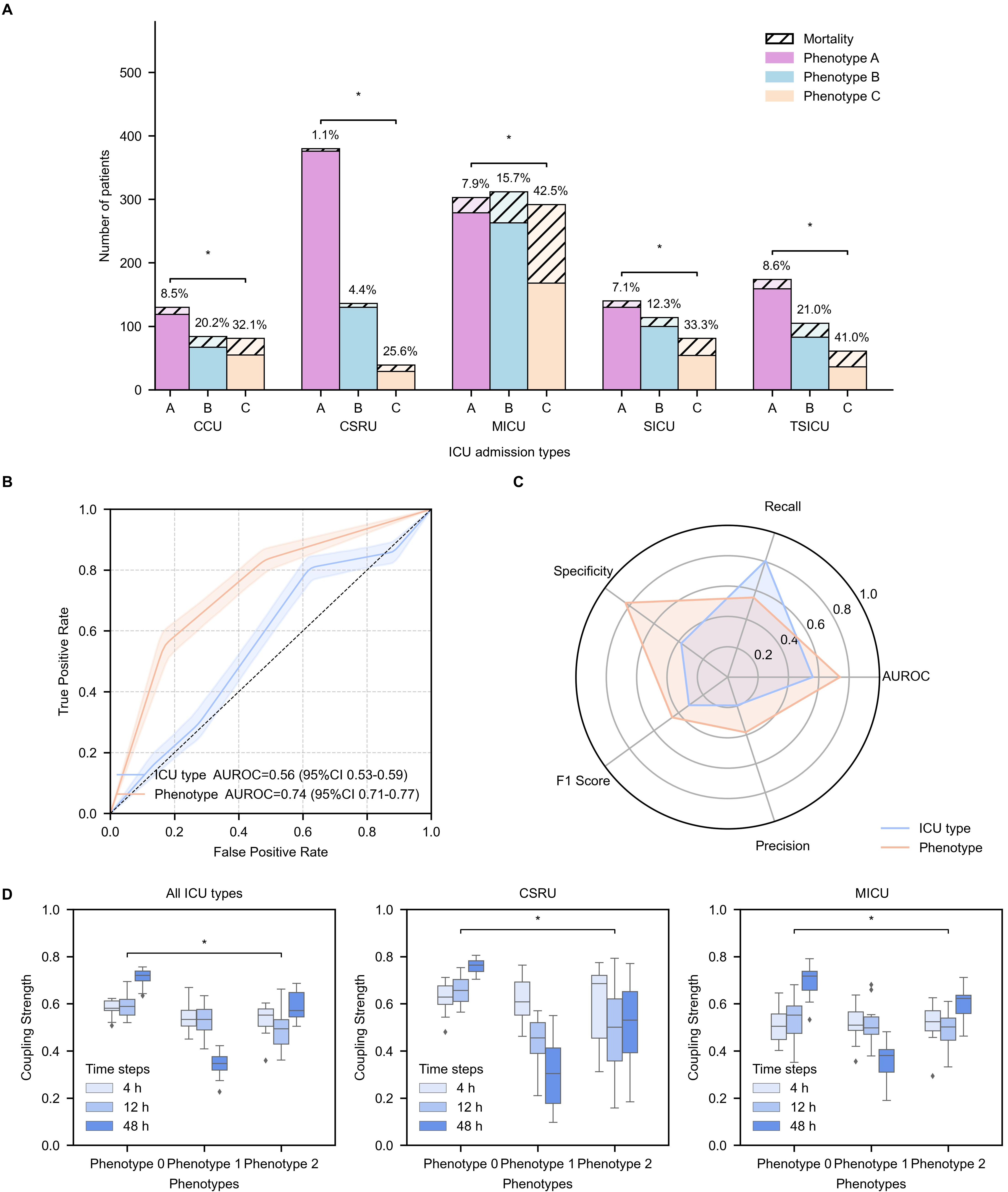


Supplementary Fig. S9. Analysis of ICU type and phenotype associations. (A) Patient counts and in-hospital mortality rates of the three phenotypes across different ICU types. (B) ROC curves comparing the predictive performance of univariate logistic regression models using either phenotype or ICU type alone for mortality prediction. (C) Radar plot showing performance metrics (AUROC, specificity, F1 score, precision) for the two univariate models; the phenotype-based model consistently outperforms the ICU-type model. (D) Boxplots of organ coupling strength across phenotypes and ICU types at different time points (4 h, 12 h, 48 h). The *x*-axis indicates phenotype groups, the *y*-axis shows coupling strength, and box color denotes time points. *P $<$ 0.05 indicates statistically significant differences.

## **Supplementary Fig. S10**


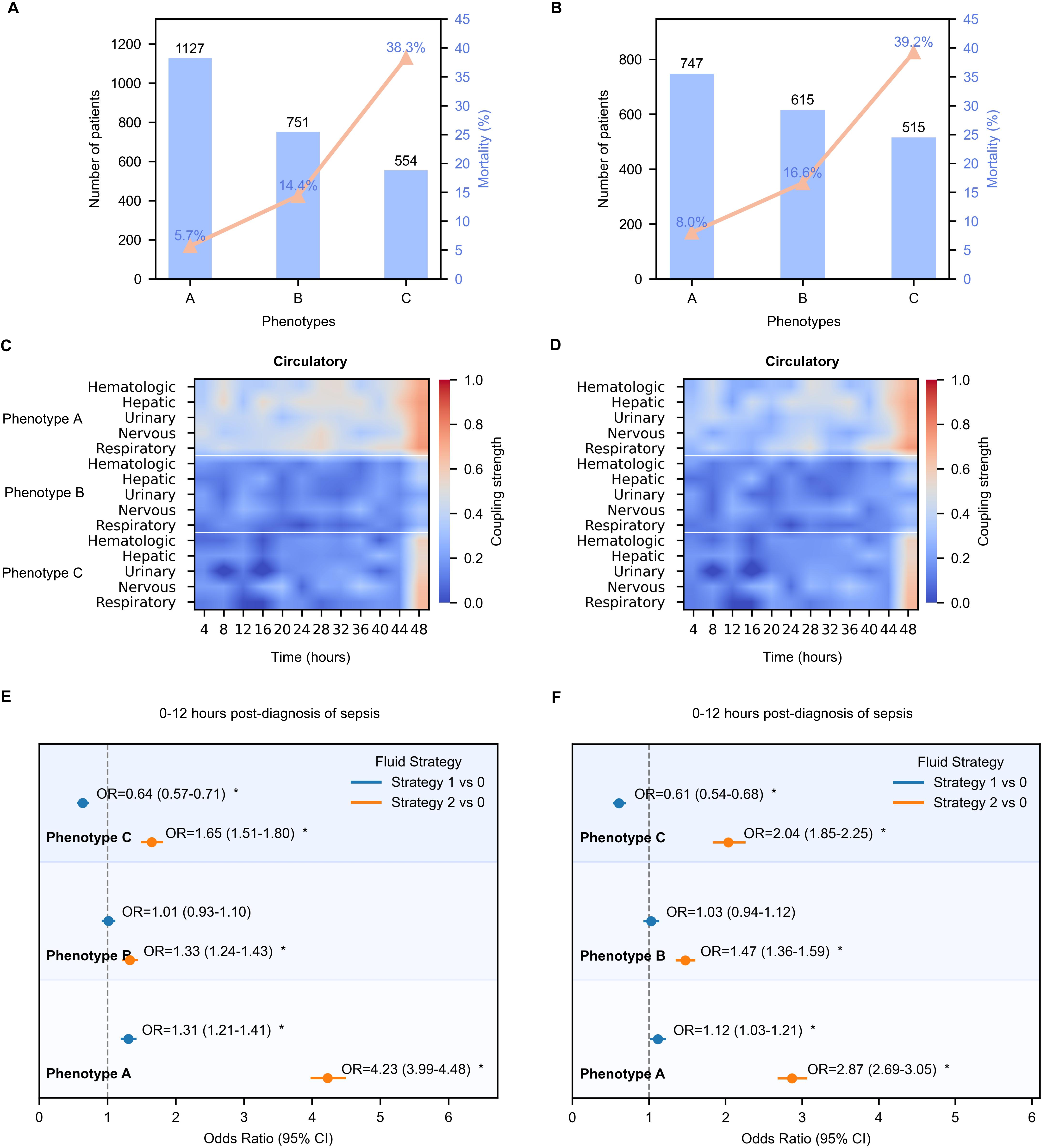


Supplementary Fig. S10. Sensitivity analysis excluding CSRU patients. (A) Mortality and patient distributions across phenotypes without excluding CSRU patients. The left *y*-axis indicates patient count; the right *y*-axis indicates mortality. (B) Mortality and patient distributions across phenotypes after excluding CSRU patients. (C) Asynchronous coupling patterns between the circulatory system and other organ systems across phenotypes without excluding CSRU patients. The *x*-axis represents time steps; color intensity denotes coupling strength. (D) Asynchronous coupling patterns between the circulatory system and other organ systems across phenotypes after excluding CSRU patients. *P<0.05 indicates statistical significance.

## **Supplementary Fig. S11**


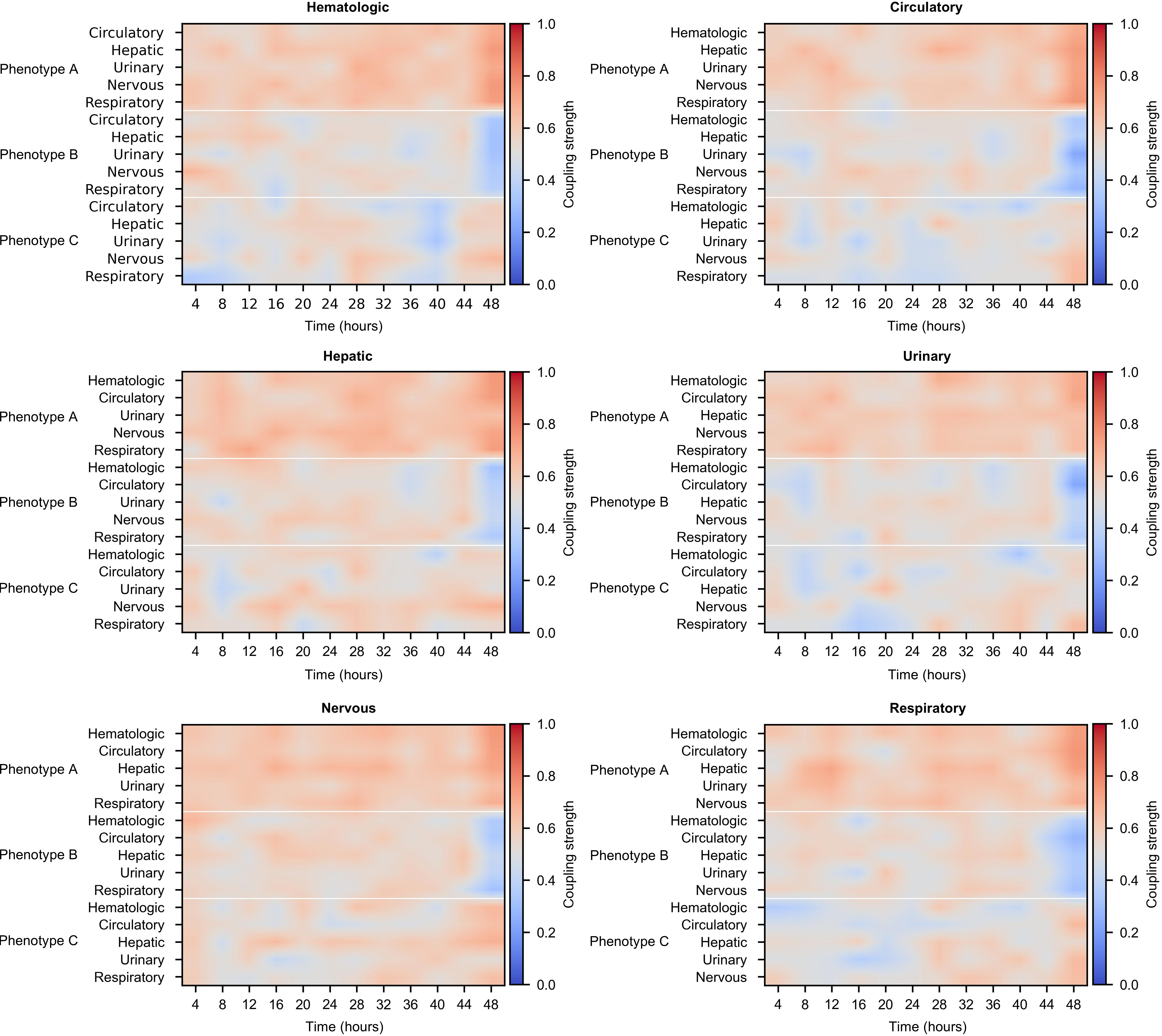


Supplementary Fig. S11. Heatmaps of inter-organ states coupling strength over time across phenotypes. Time window spans from 0 to 48 hours after sepsis diagnosis. The included organ systems are Hematologic, Circulatory, Hepatic, Urinary, Nervous, and Respiratory. Heatmap color intensity reflects the magnitude of synchronous coupling strength between organ systems. Significant differences in coupling patterns were observed among the phenotypes (P < 0.05).

## **Supplementary Fig. S12**


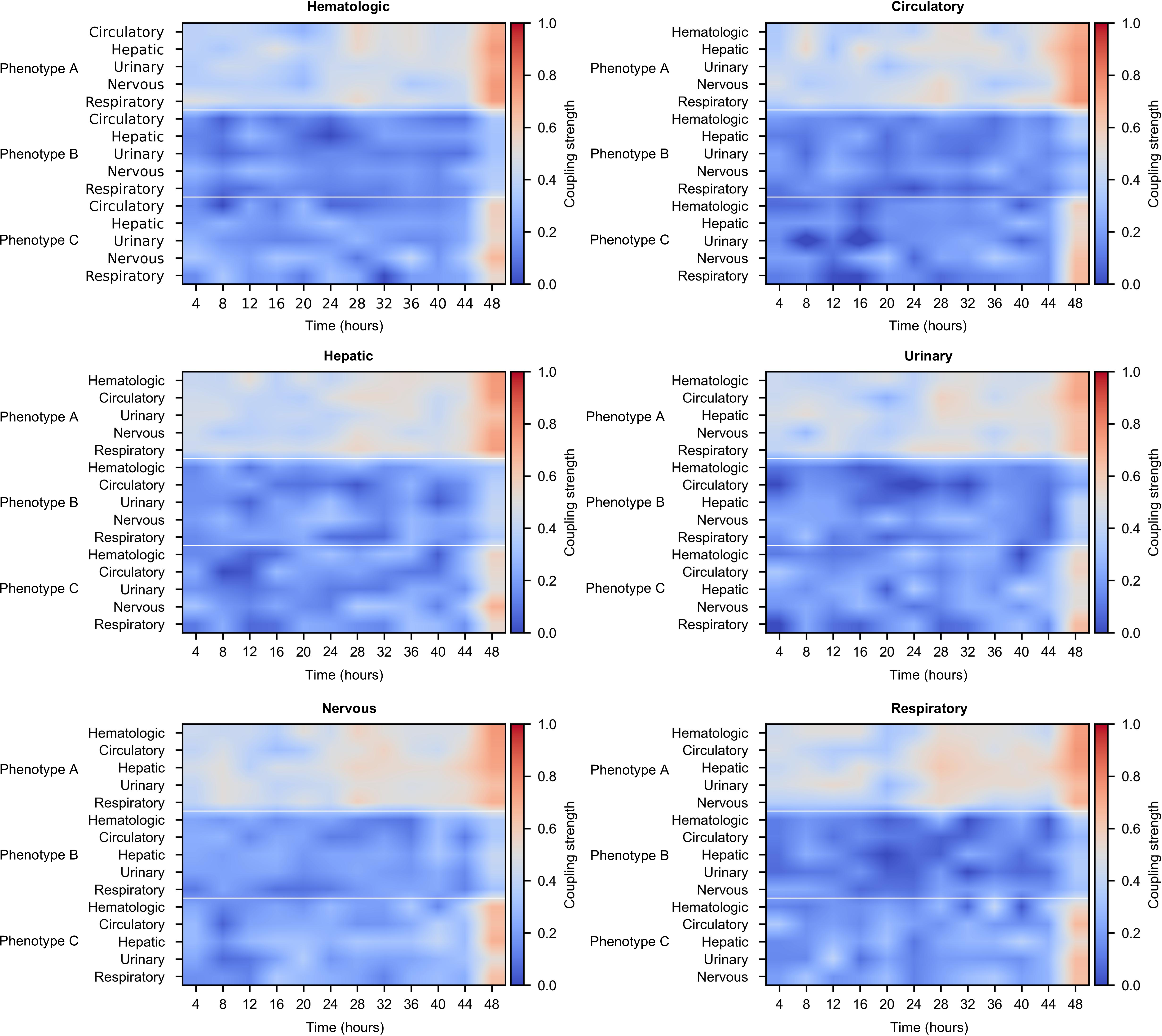


Supplementary Fig. S12. Heatmaps of coupling strength between current and historical organ states across phenotypes. Time steps span from 0 to 48 hours after sepsis diagnosis. The organ systems analyzed include Hematologic, Circulatory, Hepatic, Urinary, Nervous, and Respiratory systems. Heatmap color intensity reflects the coupling strength between the current (48-hour) organ states and their respective historical states. Significant differences in coupling patterns were observed across phenotypes (P < 0.05).

## **Supplementary Fig. S13**


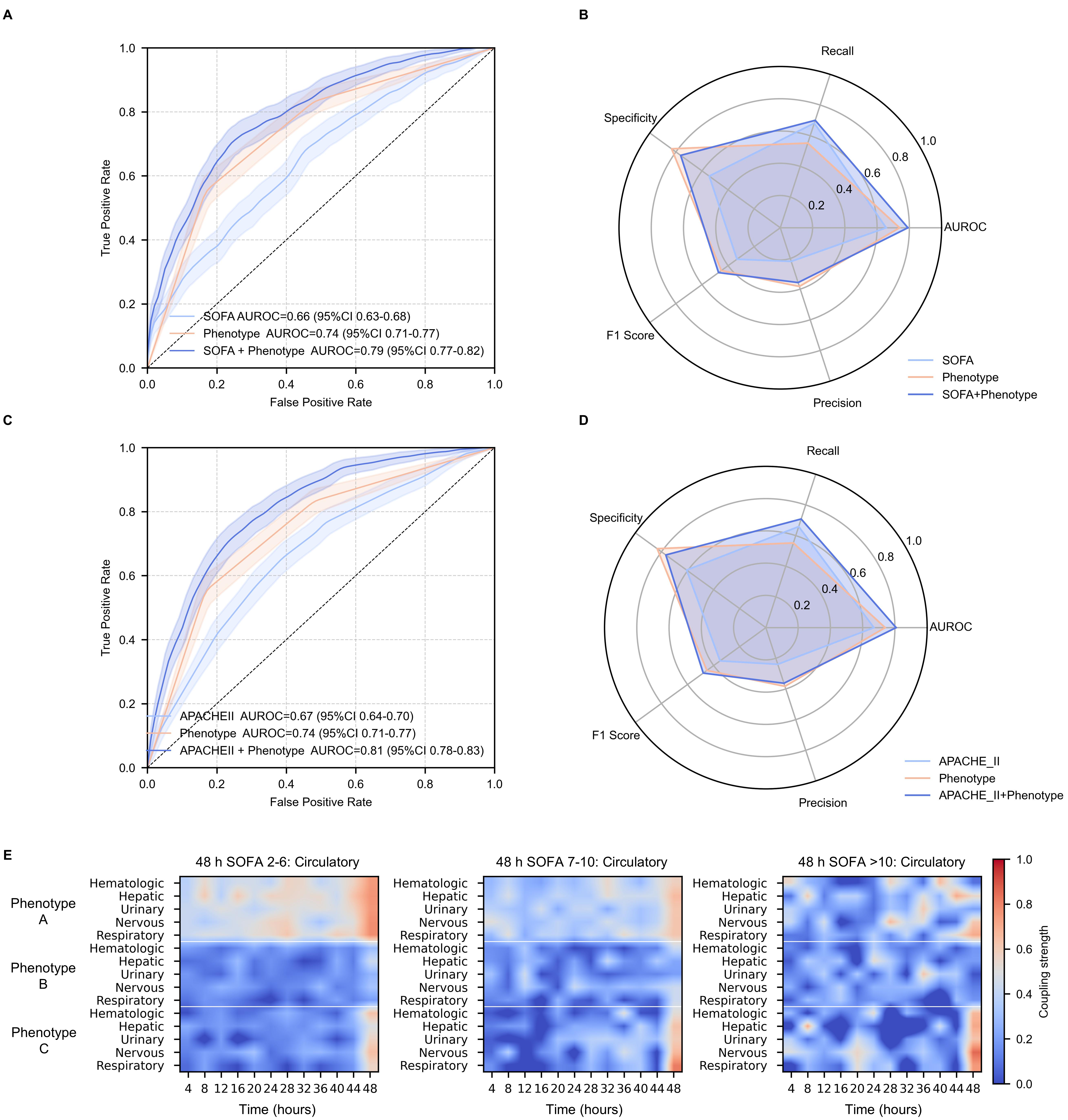


Supplementary Fig. S13. Analysis of SOFA scores and phenotype stratification. (A) ROC curves from logistic regression models predicting mortality using either SOFA scores (including baseline and 48-hour change) or phenotype alone. (B) Radar plots of predictive performance metrics from the logistic regression models (SOFA), including AUROC, F1 Score, Precision, Recall, and Specificity. (C) ROC curves from logistic regression models predicting mortality using either APACHE II scores or phenotype alone. (D) Radar plots of predictive performance metrics from the logistic regression models (APACHE II), including AUROC, F1 Score, Precision, Recall, and Specificity. (E) Asynchronous coupling strength between the circulatory system and other organ systems states across different SOFA groups and phenotypes. The *x*-axis represents time steps, and color intensity reflects coupling strength. Significant differences in interaction patterns across phenotypes were observed within each SOFA subgroup.

## **Supplementary Fig. S14**


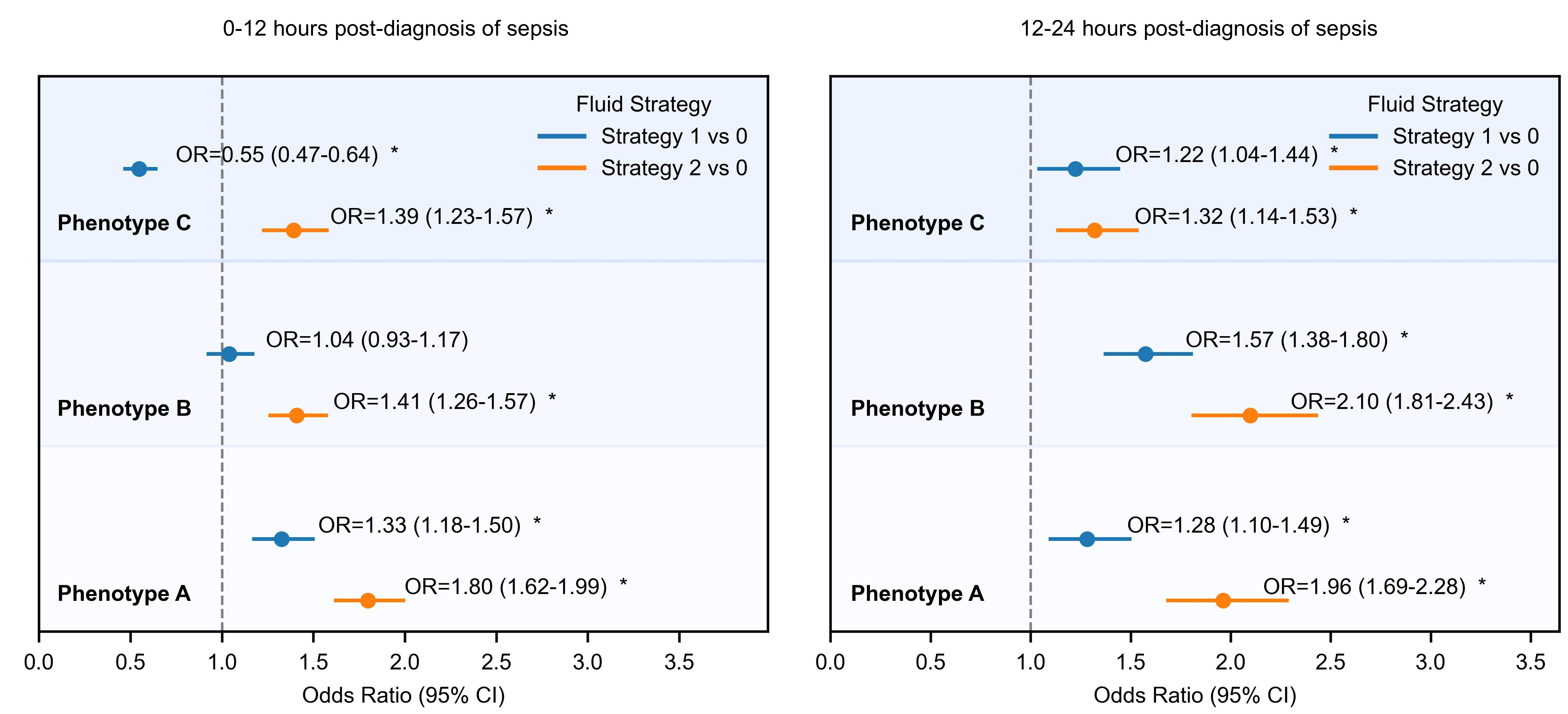


Supplementary Fig. S14. Odds ratios for mortality risk associated with fluid strategies across phenotypes in the MICU cohort, during 0-12 hours and 12-24 hours post sepsis diagnosis. *P<0.05 indicates statistical significance.

## **Supplementary Fig. S15**


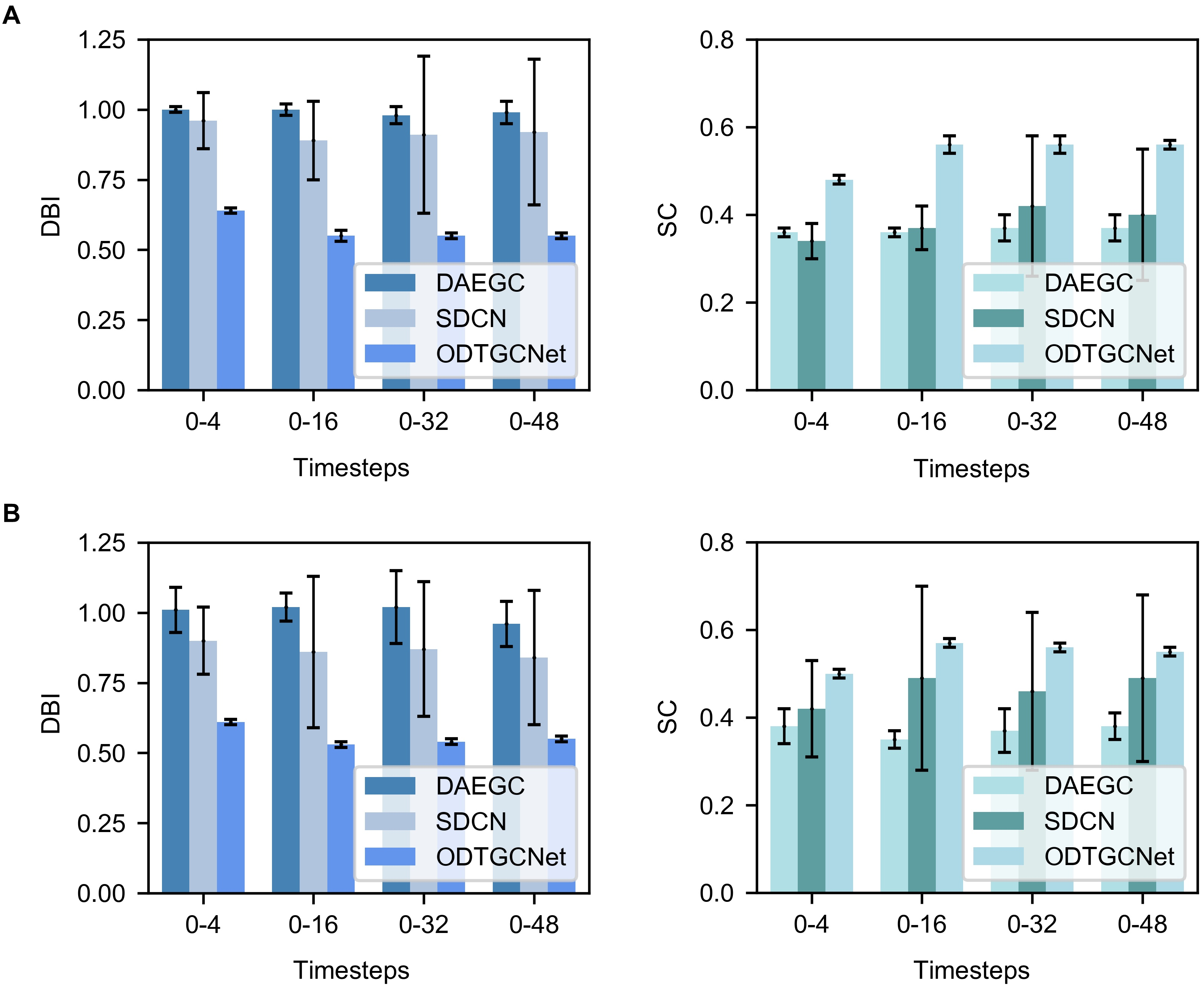


Supplementary Fig. S15. Temporal risk stratification performance across different time points. (A) DBI and SC scores on the MIMIC-III test set; (B) DBI and SC scores on the eICU test set.

## **Supplementary Fig. S16**


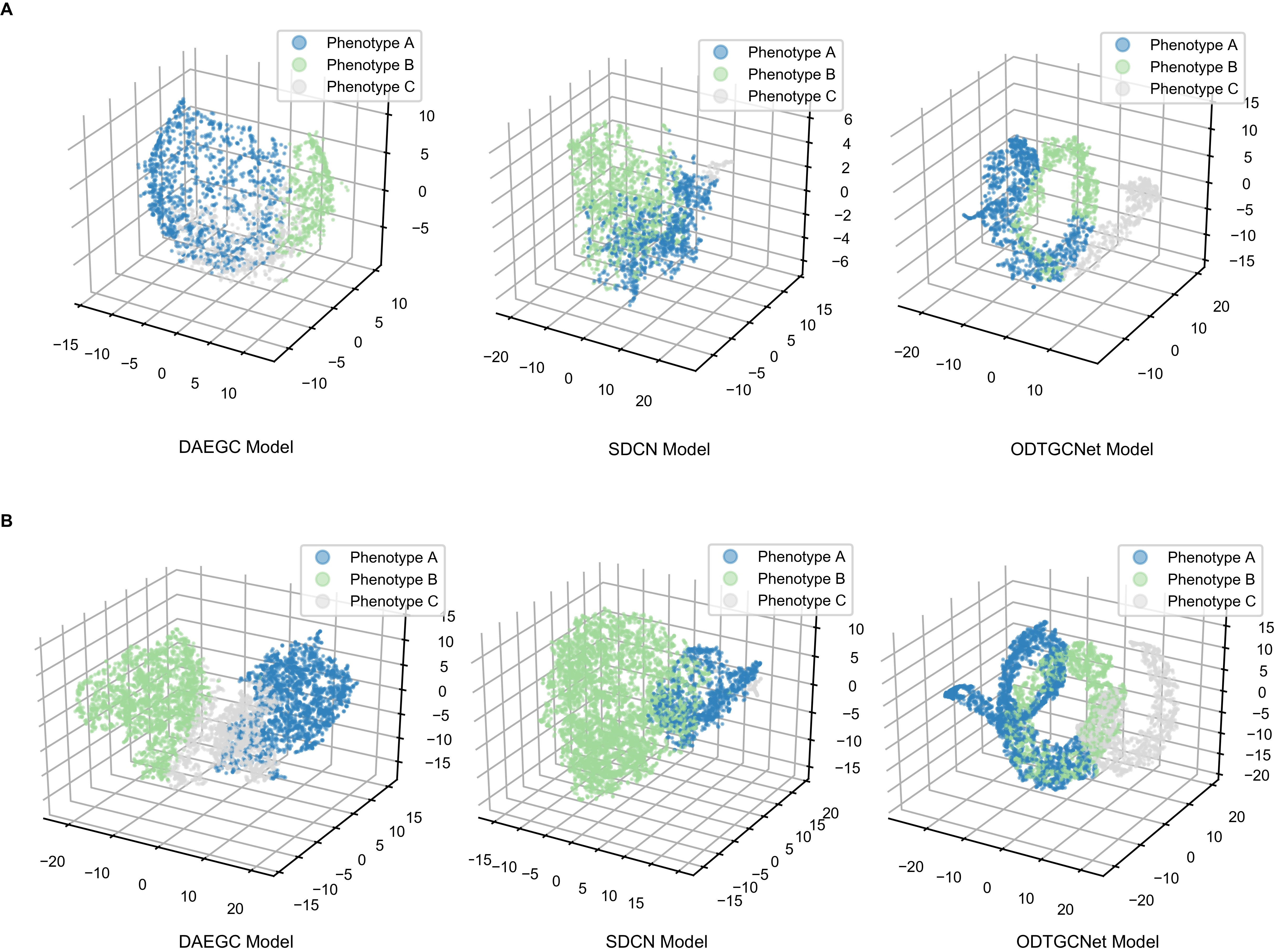


Supplementary Fig. S16. Visualization of clustering from different models. (A) Clustering on the MIMIC-III test set; (B) Clustering on the eICU test set.

## **Supplementary Fig. S17**


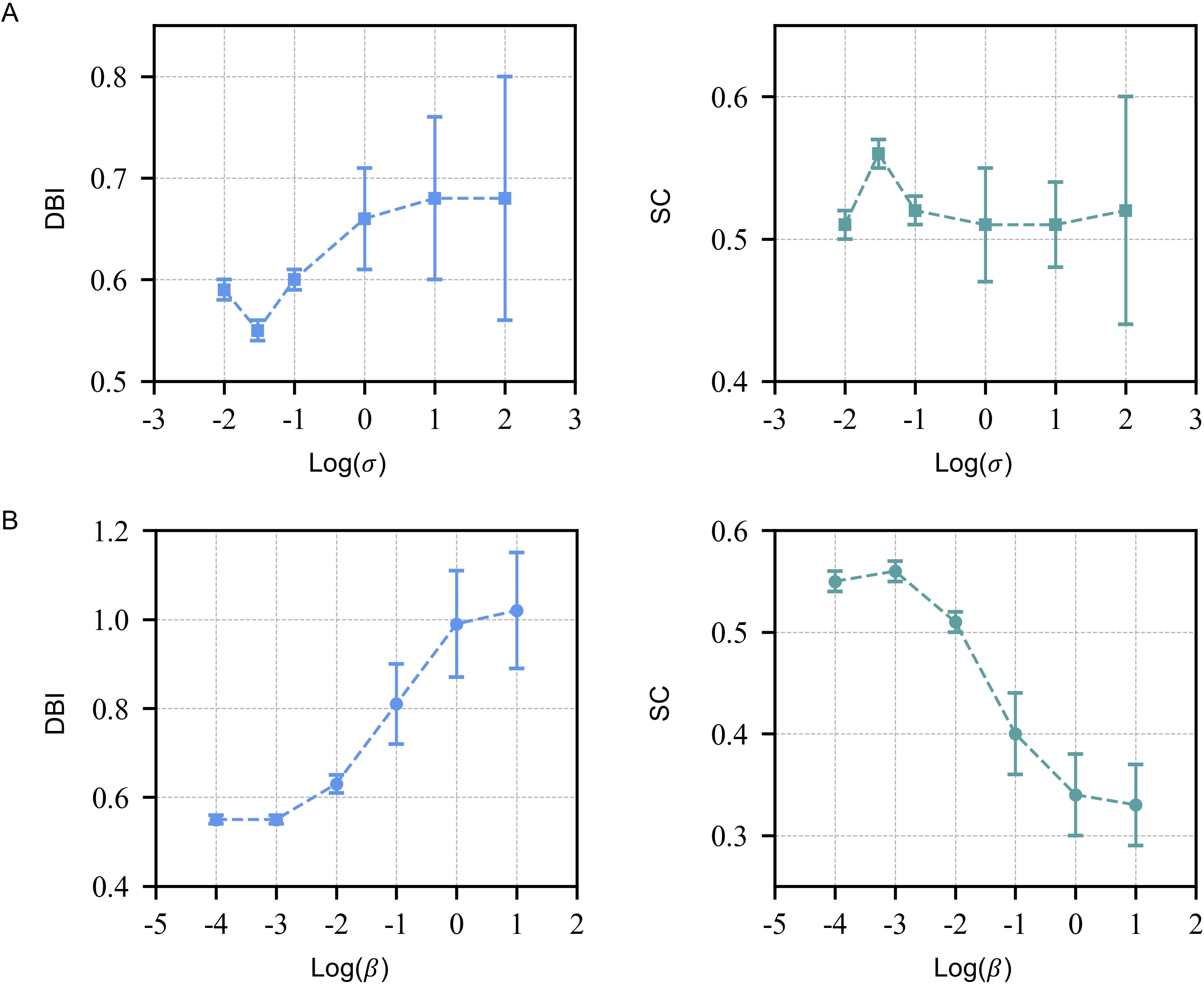


Supplementary Fig. S17. Impact of loss function coefficients on the performance of the ODTGCNet model on the MIMIC-III test set. (A) Effect of $\sigma$ on the DBI and SC scores; (B) Effect of $\beta$ on the DBI and SC scores.

## **Supplementary Fig. S18**


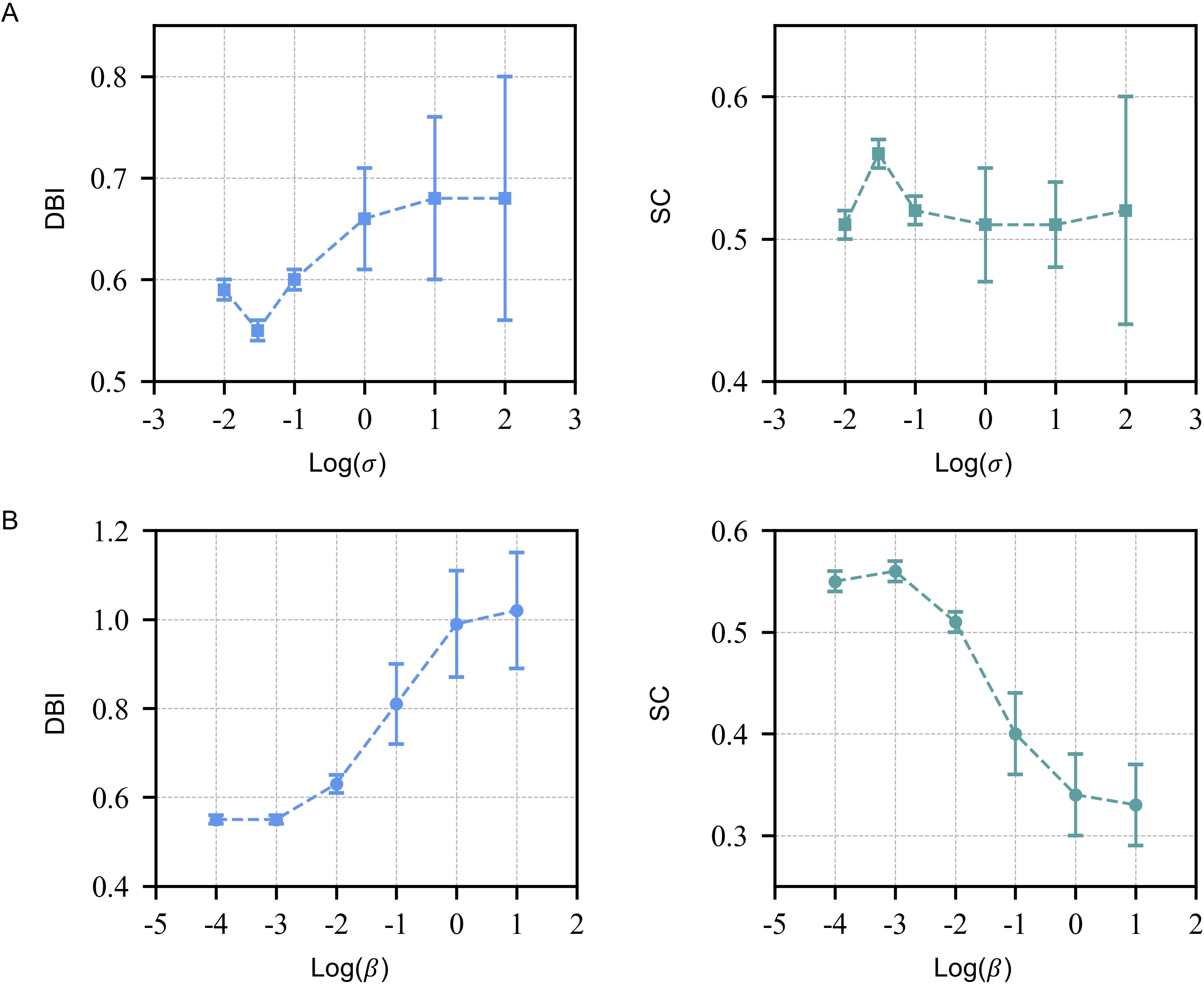


Supplementary Fig. S18. Impact of loss function coefficients on the performance of the ODTGCNet model on the eICU dataset. (A) Effect of $\sigma$ on the DBI and SC scores; (B) Effect of $\beta$ on the DBI and SC scores.

## **Supplementary Fig. S19**


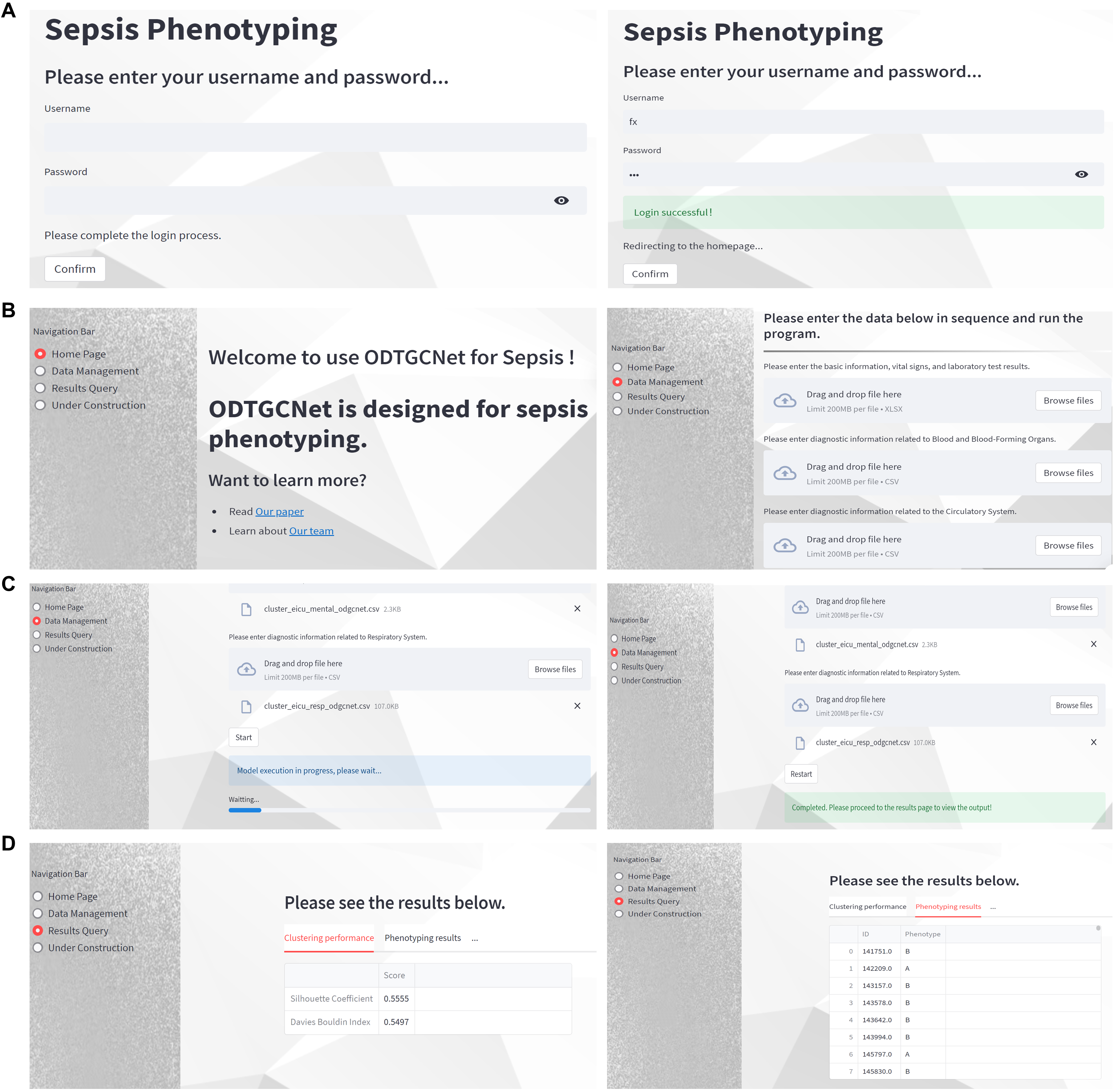


Supplementary Fig. S19. Screenshot of the sepsis clustering web interface. (A) Login page. (B) Navigation and data input interface. (C) Model execution interface. (D) Results interface, including clustering performance metrics and patient subtyping outcomes.

# **3. Supplementary Tables**

## **Supplementary Table S1**

Supplementary Table S1. Statistical Characteristics of Study Cohorts in MIMIC-III and eICU Databases.

|  | **MIMIC-III test set** | **eICU dataset** |
| --- | --- | --- |
| **Number of patients** | 2,432 | 6,208 |
| **Demographic** |  |  |
| **Gender (%)** |  |  |
| Male | 1,404 (57.7) | 3,240 (52.2) |
| Female | 1,028 (42.3) | 2,968 (47.8) |
| Age, years (mean (std)) | 65.41 (16.54) | 65.10 (15.87) |
| Weight, kg (mean (std)) | 83.09 (22.79) | 83.47 (29.00) |
| **Comorbidities (%)** |  |  |
| Cardiovascular diseases | 2,121 (87.2) | 5,540 (89.2) |
| Neurological diseases | 744 (30.6) | 1,950 (31.4) |
| Respiratory diseases | 1,463 (60.2) | 4,235 (68.2) |
| Hematologic diseases | 1,061 (43.6) | 1,148 (18.5) |
| Gastrointestinal diseases | 1,083 (44.5) | 984 (15.9) |
| Renal diseases | 1,245 (51.2) | 2,951 (47.5) |
| **Clinical scores (mean (std))** |  |  |
| SOFA score | 5.00 (2.71) | 5.27 (2.19) |
| GCS score | 14.42 (1.46) | 12.33 (2.75) |
| ECI score | 0.16 (1.81) | 2.05 (3.96) |
| **Vital signs** |  |  |
| Respiratory rate, breaths/min | 19.11 (4.51) | 21.78 (6.55) |
| Heart rate, beats/min | 88.53 (16.29) | 92.38 (18.05) |
| Systolic blood pressure, mmHg | 117.36 (16.27) | 117.44 (20.22) |
| Diastolic blood pressure, mmHg | 59.98 (10.41) | 64.40 (12.60) |
| Mean arterial pressure, mmHg | 77.66 (11.06) | 79.37 (14.33) |
| Oxygen saturation, % | 97.56 (2.55) | 96.68 (3.18) |
| Body temperature, °C | 36.88 (0.78) | 36.94 (0.69) |
| **Outcome** |  |  |
| ICU length of stay, days (mean (std)) | 6.88 (8.12) | 5.05 (5.54) |
| Hospital length of stay, days (mean (std)) | 13.80 (12.67) | 10.90 (10.13) |
| In-hospital mortality (%) | 384 (15.8) | 1,015 (16.3) |

SOFA (Sequential Organ Failure Assessment), GCS (Glasgow Coma Scale), ECI (Elixhauser Comorbidity Index).

## **Supplementary Table S2**

Supplementary Table S2. Sepsis organ interaction graph nodes and node features.

| **Node classification** | **Node name** | **Node features** |
| --- | --- | --- |
| **Patient nodes** | **Demographics** | age, sex, weight |
| **Physiological system nodes** | **Respiratory** | #5 community-acquired pneumonia, hospital-acquired pneumonia, chronic obstructive pulmonary disease, aspiration pneumonia, pleural effusion, acute respiratory failure |
|  | **Hematologic** | #4 acute posthemorrhagic anemia, unspecified anemia, disseminated intravascular coagulation, unspecified thrombocytopenia |
|  | **Hepatic** | #5 gastroesophageal reflux disease/reflux disease, acute/subacute hepatic necrosis, acute liver failure (severe hepatitis), alcoholic cirrhosis, cirrhosis (non-alcoholic), portal hypertension |
|  | **Circulatory** | #10 essential (primary) hypertension (unspecified), hypertensive renal disease (unspecified, without renal failure), hypertensive renal disease (with renal failure), subendocardial myocardial infarction (initial), old myocardial infarction, coronary atherosclerosis, mitral valve disorders; mitral regurgitation (mitral valve insufficiency), aortic valve disease; aortic stenosis, aortic regurgitation (aortic insufficiency), atrial fibrillation and flutter, acute heart failure |
|  | **Nervous** | #2 tobacco use disorder; nicotine dependence, depressive disorder (not elsewhere classified) |
|  | **Renal** | #4 acute kidney failure, acute kidney failure with tubular necrosis, chronic kidney disease, urinary tract infection; bacteriuria |
| **Physiological feature nodes** | **Respiratory** | carbon dioxide, arterial oxygen partial pressure, arterial carbon dioxide partial pressure, fraction of inspired oxygen, PaO_2_/FiO_2_, respiratory rate, oxygen saturation, pH value |
|  | **Hematologic** | international normalized ratio, prothrombin time, activated clotting time, platelet count |
|  | **Hepatic** | total bilirubin, albumin |
|  | **Circulatory** | white blood cell count, chloride, calcium, magnesium, potassium, sodium, bicarbonate, blood glucose, heart rate, systolic blood pressure, diastolic blood pressure, mean arterial pressure, body temperature, lactate, hemoglobin, base excess |
|  | **Nervous** | Glasgow coma scale |
|  | **Renal** | blood urea nitrogen, creatinine |

## **Supplementary Table S3**

Supplementary Table S3. The algorithmic pseudocode of the ODTGCNet model.

| **The algorithmic of the ODTGCNet** |
| --- |
| **Input:** Sepsis graph $G$, Node feature $X$, Number of clusters $k$, Epochs $E$, Total number of time steps $T$, Layers of GAT units |
| **Output:** Clustering results $C$ |
| **Initialization**: Use the K-Means algorithm to initialize cluster centers $\mu$ |
| ***for*** $e=1$ ***to*** $E$ |
| According to Eq. (11), compute the predicted label representation for the first time step |
| ***for*** $t=1$ ***to*** $T$ |
| According to Eq. (12), compute the predicted label $\hat{Y}$ for the first time step |
| Generate the representations [$Z^{1},\ldots,Z^{N-1}]$and the reconstructed feature $\tilde{X}$ using the conditional autoencoder as defined in Eq. (1) |
| According to Eq. (10), compute the attention output representation $\tilde{g}_{0}^{'}$ of the first layer of the GAT unit |
| ***for*** $n=1$ ***to*** $N$ |
| According to Eq. (7) of the adaptive information fusion layer, compute the fused representation $\tilde{g}_{n}$ |
| Based on Eq. (10), compute the output representation ${\tilde{G}^{n}}^{'}$ of the GAT unit |
| ***end*** |
| According to Eq. (17), compute the reconstructed matrix $\hat{A}$ |
| ***end*** |
| According to Eq. (12), compute the predicted label $\hat{Y}$ |
| Using Eq. (14) and (15), compute the distribution $Q$and the auxiliary distribution $P$ |
| Based on Eq. (5), (13), (16), (18), and (19), compute $\mathcal{L}_{CVAE}$, $\mathcal{L}_{C}$, $\mathcal{L}_{G}$, $\mathcal{L}_{AR}$, and $\mathcal{L}_{S}$ |
| According to Eq. (20), compute the loss function $\mathcal{L}$ |
| Perform backpropagation to update the model parameters |
| ***end for*** |
| According to Eq. (12), output the clustering result $C$ |

## **Supplementary Table S4**

Supplementary Table S4. Characteristics of sepsis phenotypes in the MIMIC-III database.

| **Characteristics** | **All patients** | **Phenotypes** | | | **P value** |
| --- | --- | --- | --- | --- | --- |
|  |  | **A** | **B** | **C** |  |
| **Number of patients** | 6,208 | 2,734 (44.0) | 1,917 (30.9) | 1,557 (25.1) |  |
| **Demographic information** |  |  |  |  |  |
| **Gender (%)** |  |  |  |  | 0.083 |
| Male | 3,240 (52.2) | 1,470 (53.8) | 980 (51.1) | 790 (50.7) |  |
| Female | 2,968 (47.8) | 1,264 (46.2) | 937 (48.9) | 767 (49.3) |  |
| **Race (%)** |  |  |  |  | 0.026* |
| African American | 556 (9.0) | 275 (10.1) | 158 (8.2) | 123 (7.9) |  |
| Asian | 129 (2.1) | 63 (2.3) | 36 (1.9) | 30 (1.9) |  |
| White | 4,720 (76.0) | 2,056 (75.2) | 1,478 (77.1) | 1,186 (76.2) |  |
| Hispanic | 361 (5.8) | 145 (5.3) | 110 (5.7) | 106 (6.8) |  |
| Native American | 56 (0.9) | 29 (1.1) | 16 (0.8) | 11 (0.7) |  |
| Unknown | 386 (6.2) | 166 (6.1) | 119 (6.2) | 101 (6.5) |  |
| Age, years (mean (std)) | 65.10 (15.87) | 58.23 (15.73) | 67.08 (14.30) | 74.40 (11.77) | 0.000* |
| Weight, kg (mean (std)) | 83.47 (29.00) | 87.99 (30.83) | 82.50 (30.08) | 76.71 (22.11) | 0.000* |
| **ICU types (%)** |  |  |  |  | 0.301 |
| Cardiac ICU | 367 (5.9) | 166 (6.1) | 112 (5.8) | 89 (5.7) |  |
| CCU-CTICU | 412 (6.6) | 199 (7.3) | 106 (5.5) | 107 (6.9) |  |
| CSICU | 156 (2.5) | 76 (2.8) | 39 (2.0) | 41 (2.6) |  |
| CTICU | 46 (0.7) | 18 (0.7) | 14 (0.7) | 14 (0.9) |  |
| Med-Surg ICU | 4,030 (64.9) | 1,740 (63.6) | 1,274 (66.5) | 1,016 (65.3) |  |
| MICU | 728 (11.7) | 310 (11.3) | 222 (11.6) | 196 (12.6) |  |
| Neuro ICU | 167 (2.7) | 74 (2.7) | 56 (2.9) | 37 (2.4) |  |
| SICU | 302 (4.9) | 151 (5.5) | 94 (4.9) | 57 (3.7) |  |
| **Comorbidities (%)** |  |  |  |  |  |
| Cardiovascular diseases | 5,540 (89.2) | 2,377 (86.9) | 1,738 (90.7) | 1,425 (91.5) | 0.000* |
| Neurological diseases | 1,950 (31.4) | 828 (30.3) | 585 (30.5) | 537 (34.5) | 0.010* |
| Respiratory diseases | 4,235 (68.2) | 1,687 (61.7) | 1,356 (70.7) | 1,192 (76.6) | 0.000* |
| Hematologic diseases | 1,148 (18.5) | 456 (16.7) | 350 (18.3) | 342 (22.0) | 0.000* |
| Gastrointestinal diseases | 984 (15.9) | 410 (15.0) | 307 (16.0) | 267 (17.1) | 0.174 |
| Renal diseases | 2,951 (47.5) | 1150 (42.1) | 923 (48.1) | 878 (56.4) | 0.000* |
| **Clinical scores** (mean (std)) |  |  |  |  |  |
| SOFA score | 5.27 (2.19) | 4.91 (2.00) | 5.31 (2.10) | 5.88 (2.47) | 0.000* |
| GCS score | 12.33 (2.75) | 12.80 (2.50) | 12.25 (2.76) | 11.60 (2.98) | 0.000* |
| ECI score | 2.05 (3.96) | 1.31 (3.18) | 2.03 (3.98) | 3.36 (4.77) | 0.000* |
| **Vital signs** |  |  |  |  |  |
| Respiratory rate, breaths/min | 21.78 (6.55) | 21.07 (6.04) | 21.86 (6.70) | 22.92 (7.04) | 0.000* |
| Heart rate, beats/min | 92.38 (18.05) | 93.35 (18.36) | 92.61 (17.97) | 92.16 (17.59) | 0.757 |
| Systolic blood pressure, mmHg | 117.44 (20.22) | 118.24 (20.61) | 118.05 (19.99) | 115.29 (19.69) | 0.000* |
| Diastolic blood pressure, mmHg | 64.40 (12.60) | 65.94 (12.93) | 64.43 (12.42) | 61.65 (11.73) | 0.000* |
| Mean arterial pressure, mmHg | 79.37 (14.33) | 80.54 (14.65) | 79.51 (14.10) | 77.15 (13.78) | 0.000* |
| Oxygen saturation, % | 96.68 (3.18) | 96.81 (2.82) | 96.66 (3.15) | 96.49 (3.77) | 0.397 |
| Body temperature, $^{\circ}$C | 36.94 (0.69) | 36.98 (0.69) | 36.94 (0.69) | 36.85 (0.69) | 0.000* |
| **Outcome** |  |  |  |  |  |
| ICU length of stay, days (mean (std)) | 5.05 (5.54) | 4.60 (5.44) | 5.23 (5.56) | 5.61 (5.64) | 0.000* |
| Hospital length of stay, days (mean (std)) | 10.90 (10.13) | 10.67 (9.83) | 11.33 (10.72) | 10.81 (9.87) | 0.039* |
| In-hospital mortality (%) | 1,015 (16.3) | 232 (8.5) | 316 (16.5) | 467 (30.0) | 0.000* |

SOFA (Sequential Organ Failure Assessment), GCS (Glasgow Coma Scale), ECI (Elixhauser Comorbidity Index).

*P<0.05 has significant different.

## **Supplementary Table S5**

Supplementary Table S5. Baseline characteristics of patients with different phenotypes in the CCU cohort (MIMIC-III).

| **Characteristics** | **Phenotypes** | | | **P value** |
| --- | --- | --- | --- | --- |
|  | **A** | **B** | **C** |  |
| **Number of patients** | 130 | 84 | 81 |  |
| **Demographics** |  | | |  |
| Age, years (mean (std)) | 67.58 (15.14) | 69.86 (15.18) | 75.51 (12.65) | 0.001* |
| Weight, kg (mean (std)) | 84.29 (24.97) | 81.28 (24.50) | 76.48 (21.11) | 0.037* |
| Male (%) | 86 (66.15) | 54 (64.29) | 39 (48.15) | 0.025* |
| **Race (%)** |  | | | 0.462 |
| Asian | 3 (2.321) | 2 (2.38) | 1 (1.23) |  |
| Black | 5 (3.85) | 10 (11.90) | 8 (9.88) |  |
| Hispanic | 1 (0.77) | 1 (1.19) | 1 (1.23) |  |
| White | 93 (71.64) | 50 (59.52) | 14 (70.37) |  |
| Unknown | 28 (21.54) | 21 (25.00) | 57 (17.28) |  |
| **Comorbidities (%)** |  | | |  |
| Cardiovascular diseases | 126 (96.92) | 80 (95.23) | 79 (97.53) | 0.694 |
| Neurological diseases | 43 (33.08) | 29 (34.52) | 19 (23.46) | 0.234 |
| Respiratory diseases | 71 (54.61) | 55 (65.47) | 61 (75.53) | 0.009* |
| Hematologic diseases | 54 (41.54) | 36 (42.86) | 36 (44.44) | 0.917 |
| Gastrointestinal diseases | 49 (37.69) | 54 (64.28) | 28 (34.57) | 0.789 |
| Renal diseases | 68 (52.31) |  | 61 (75.31) | 0.004* |
| **Outcome** |  | | |  |
| ICU length of stay, days (mean (std)) | 7.97 (12.74) | 7.32 (7.08) | 8.09 (8.54) | 0.131 |
| Hospital length of stay, day (mean (std)) | 12.30 (13.56) | 13.92 (13.07) | 14.07 (11.86) | 0.108 |
| In-hospital mortality (%) | 11 (8.46) | 14 (20.24) | 26 (32.10) | 0.000* |

*P<0.05 has significant different.

## **Supplementary Table S6**

Supplementary Table S6. Baseline characteristics of patients with different phenotypes in the CSRU cohort (MIMIC-III).

| **Characteristics** | **Phenotypes** | | | **P value** |
| --- | --- | --- | --- | --- |
|  | **A** | **B** | **C** |  |
| **Number of patients** | 380 | 136 | 39 |  |
| **Demographics** |  | | |  |
| Age, years (mean (std)) | 68.71 (11.35) | 70.94 (11.63) | 75.15 (12.19) | 0.001* |
| Weight, kg (mean (std)) | 85.74 (21.94) | 79.58 (19.10) | 77.73 (17.95) | 0.001* |
| Male (%) | 246 (64.73%) | 75 (55.15%) | 27 (69.23) | 0.095 |
| **Race (%)** |  | | | 0.117 |
| Asian | 2 (0.53) | 2 (1.47) | 0 |  |
| Black | 16 (4.21) | 5 (3.67) | 0 |  |
| Hispanic | 14 (3.68) | 1 (0.74) | 0 |  |
| White | 276 (72.63) | 29 (72.79) | 25 (64.10) |  |
| Unknown | 72 (18.95) | 99 (21.32) | 14 (35.90 |  |
| **Comorbidities (%)** |  | | |  |
| Cardiovascular diseases | 379 (99.74) | 135 (99.26) | 39 (100) | 0.679 |
| Neurological diseases | 87 (22.89) | 24 (17.65) | 4 (10.26) | 0.107 |
| Respiratory diseases | 131 (34.47) | 54 (39.70) | 22 (56.41) | 0.021* |
| Hematologic diseases | 123 (32.37) | 41 (30.15) | 13 (33.33) | 0.874 |
| Gastrointestinal diseases | 113 (29.74) | 39 (28.68) | 17 (43.59) | 0.176 |
| Renal diseases | 114 (30.00 | 50 (36.76) | 21 (53.85) | 0.007* |
| **Outcome** |  | | |  |
| ICU length of stay, days (mean (std)) | 4.08 (3.19) | 6.57 (8.03) | 13.16 (25.11) | 0.000* |
| Hospital length of stay, day (mean (std)) | 9.62 (5.94) | 12.19 (8.39) | 17.88 (24.35) | 0.000* |
| In-hospital mortality (%) | 4 (1.05%) | 6 (4.41%) | 10 (25.64%) | 0.000* |

*P<0.05 has significant different.

## **Supplementary Table S7**

Supplementary Table S7. Baseline characteristics of patients with different phenotypes in the MICU cohort (MIMIC-III).

| **Characteristics** | **Phenotypes** | | | **P value** |
| --- | --- | --- | --- | --- |
|  | **A** | **B** | **C** |  |
| **Number of patients** | 303 | 312 | 292 |  |
| **Demographics** |  | | |  |
| Age, years (mean (std)) | 60.11 (17.17) | 65.05 (16.74) | 68.19 (16.52) | 0.000* |
| Weight, kg (mean (std)) | 87.66 (25.66) | 83.86 (27.59) | 79.32 (20.82) | 0.000* |
| Male (%) | 154 (50.83) | 158 (50.64) | 160 (54.79) | 0.519 |
| **Race (%)** |  | | | 0.111* |
| Asian | 8 (2.64) | 5 (1.60) | 7 (2.39) |  |
| Black | 34 (11.22) | 29 (9.29) | 30 (10.27) |  |
| Hispanic | 14 (4.62) | 10 (3.21) | 7 (2.40) |  |
| White | 218 (71.95) | 237 (75.96) | 199 (68.15) |  |
| Unknown | 29 (9.57) | 31 (9.94) | 49 (16.78) |  |
| **Comorbidities (%)** |  | | |  |
| Cardiovascular diseases | 252 (83.17) | 274 (87.82) | 248 (84.93) | 0.257 |
| Neurological diseases | 95 (31.35) | 113 (36.22) | 81 (27.74) | 0.080 |
| Respiratory diseases | 214 (70.63) | 241 (77.24) | 222 (76.03) | 0.136 |
| Hematologic diseases | 184 (60.73) | 164 (52.56) | 177 (60.61) | 0.063 |
| Gastrointestinal diseases | 151 (49.84) | 172 (55.13) | 183 (62.67) | 0.007* |
| Renal diseases | 170 (56.11) | 201 (64.42) | 225 (77.05) | 0.000* |
| **Outcome** |  | | |  |
| ICU length of stay, days (mean (std)) | 6.39 (7.03) | 6.48 (6.68) | 7.29 (8.25) | 0.125 |
| Hospital length of stay, day (mean (std)) | 13.00 (11.28) | 14.16 (12.57) | 13.73 (13.20) | 0.182 |
| In-hospital mortality (%) | 24 (7.92) | 49 (15.70) | 124 (42.47) | 0.000* |

*P<0.05 has significant different.

## **Supplementary Table S8**

Supplementary Table S8. Baseline characteristics of patients with different phenotypes in the SICU cohort (MIMIC-III).

| **Characteristics** | **Phenotypes** | | | **P value** |
| --- | --- | --- | --- | --- |
|  | **A** | **B** | **C** |  |
| **Number of patients** | 140 | 114 | 81 |  |
| **Demographics** |  | | |  |
| Age, years (mean (std)) | 60.68 (15.25) | 63.81 (15.63) | 69.83 (14.83) | 0.000* |
| Weight, kg (mean (std)) | 85.99 (25.46) | 82.01 (17.40) | 77.75 (16.84) | 0.039* |
| Male (%) | 71 (50.71) | 58 (50.88) | 50 (61.73) | 0.228 |
| **Race (%)** |  | | | 0.063 |
| Asian | 4 (2.86) | 0 | 2 (2.47) |  |
| Black | 14 (10.00) | 8 (7.02) | 6 (7.41) |  |
| Hispanic | 5 (3.57) | 7 (6.14) | 0 |  |
| White | 108 (77.14) | 87 (76.32) | 59 (72.84) |  |
| Unknown | 9 (6.43) | 12 (10.53) | 14 (17.28) |  |
| **Comorbidities (%)** |  | | |  |
| Cardiovascular diseases | 119 (85.00) | 94 (82.46) | 65 (80.25) | 0.652 |
| Neurological diseases | 55 (39.29) | 51 (44.74) | 31 (38.27) | 0.584 |
| Respiratory diseases | 73 (52.14) | 67 (58.77) | 55 (67.90) | 0.072 |
| Hematologic diseases | 52 (37.14) | 44 (38.60) | 23 (28.40) | 0.297 |
| Gastrointestinal diseases | 67 (47.86) | 59 (51.75) | 53 (65.43) | 0.038* |
| Renal diseases | 46 (32.86) | 57 (50.00) | 49 (60.49) | 0.000* |
| **Outcome** |  | | |  |
| ICU length of stay, days (mean (std)) | 6.83 (6.46) | 7.49 (7.91) | 8.92 (7.95) | 0.032* |
| Hospital length of stay, day (mean (std)) | 15.10 (15.72) | 17.46 (13.61) | 19.52 (17.19) | 0.049* |
| In-hospital mortality (%) | 10 (7.14) | 14 (12.28) | 27 (33.33) | 0.000* |

*P<0.05 has significant different.

## **Supplementary Table S9**

Supplementary Table S9. Baseline characteristics of patients with different phenotypes in the TSICU cohort (MIMIC-III).

| **Characteristics** | **Phenotypes** | | | **P value** |
| --- | --- | --- | --- | --- |
|  | **A** | **B** | **C** |  |
| **Number of patients** | 174 | 105 | 61 |  |
| **Demographics** |  | | |  |
| Age, years (mean (std)) | 49.33 (19.20) | 63.58 (18.30) | 73.99 (14.13) | 0.000* |
| Weight, kg (mean (std)) | 86.49 (19.29) | 82.09 (17.29) | 72.44 (16.49) | 0.000* |
| Male (%) | 124 (71.26) | 70 (66.67) | 32 (52.46) | 0.028* |
| **Race (%)** |  | | | 0.039* |
| Asian | 0 | 0 | 0 |  |
| Black | 15 (8.62) | 7 (6.67) | 1 (1.64) |  |
| Hispanic | 15 (8.62) | 2 (1.90) | 1 (1.64) |  |
| White | 118 (67.82) | 84 (80.00) | 49 (80.33) |  |
| Unknown | 26 (14.94) | 12 (11.43) | 10 (16.39) |  |
| **Comorbidities (%)** |  | | |  |
| Cardiovascular diseases | 108 (62.07) | 76 (72.38) | 47 (77.05) | 0.049* |
| Neurological diseases | 61 (35.06) | 34 (32.38) | 17 (27.87) | 0.583 |
| Respiratory diseases | 95 (54.60) | 65 (61.90) | 37 (60.66) | 0.436 |
| Hematologic diseases | 60 (34.48) | 31 (29.52) | 23 (37.70) | 0.521 |
| Gastrointestinal diseases | 59 (33.91) | 38 (36.19) | 27 (44.26) | 0.351 |
| Renal diseases | 44 (25.29) | 49 (46.67) | 37 (60.66) | 0.000* |
| **Outcome** |  | | |  |
| ICU length of stay, days (mean (std)) | 8.05 (7.40) | 8.51 (6.23) | 9.12 (10.33) | 0.331 |
| Hospital length of stay, day (mean (std)) | 16.65 (14.99) | 16.85 (12.29) | 15.07 (12.05) | 0.332 |
| In-hospital mortality (%) | 15 (8.62) | 22 (20.95) | 25 (40.98) | 0.000* |

*P<0.05 has significant different.

## **Supplementary Table S10**

Supplementary Table S10. Key characteristics of Phenotypes A and C at 48 hours following sepsis diagnosis.

| **Characteristics** | **Phenotype A** | **Phenotype C** | **P-value** |
| --- | --- | --- | --- |
| Creatinine (mg/dL) | 1.22 (1.09) | 1.99 (1.53) | 0.000* |
| BUN (mg/dL) | 20.24 (13.69) | 41.25 (25.77) | 0.000* |
| HCO₃⁻ (mmol/L) | 24.35 (3.68) | 21.86 (4.80) | 0.000* |
| pH | 7.40 (0.05) | 7.37 (0.07) | 0.000* |
| PaCO₂ (mmHg) | 40.88 (6.64) | 38.70 (7.75) | 0.000* |
| PaO₂ (mmHg) | 122.36 (50.83) | 119.15 (49.01) | 0.417 |
| Respiratory rate (breaths/min) | 19.17 (4.60) | 21.21 (5.44) | 0.000* |
| SpO₂ (%) | 97.03 (2.04) | 96.54 (3.19) | 0.120 |
| PaO₂/FiO₂ ratio (mmHg) | 275.32 (113.65) | 254.73 (100.41) | 0.008* |

*P<0.05 has significant different.

## **Supplementary Table S11**

Supplementary Table S11. Forest plot of mortality risk associated with phenotypes and clinical variables.

| **Variable** | **OR** | **95% CI** | **P-value** |
| --- | --- | --- | --- |
| Phenotype | 5.13 | 4.01-6.57 | 0.000* |
| APACHE_II | 1.08 | 1.04-1.11 | 0.000* |
| Age | 0.99 | 0.99-1.00 | 0.088 |
| Gender | 0.95 | 0.75-1.20 | 0.641 |

*P<0.05 indicates statistical significance.

## **Supplementary Table S12**

Supplementary Table S12. Results of likelihood ratio tests comparing models with ICU type × fluid strategy interaction terms to reduced models without interaction, conducted across all ICU types and with CSRU patients excluded. A P value < 0.05 was considered statistically significant.

|  | **Phenotypes** | **0-12 h** | **12-24 h** |
| --- | --- | --- | --- |
| All ICU types | Phenotype A | 0.021* | 0.054 |
|  | Phenotype B | 0.104 | 0.289 |
|  | Phenotype C | 0.409 | 0.159 |
| Excluding CSRU | Phenotype A | 0.345 | 0.958 |
|  | Phenotype B | 0.396 | 0.293 |
|  | Phenotype C | 0.622 | 0.854 |

*P<0.05 indicates statistical significance.

## **Supplementary Table S13**

Supplementary Table S13. Clustering performance of different models (mean(std)).

| **Models** | **MIMIC-III** | | **eICU** | |
| --- | --- | --- | --- | --- |
|  | **DBI** | **SC** | **DBI** | **SC** |
| **DEC** | 1.10 (0.13) | 0.33 (0.06) | 1.14 (0.13) | 0.31 (0.07) |
| **IDEC** | 1.09 (0.15) | 0.35 (0.08) | 1.00 (0.11) | 0.39 (0.08) |
| **DAEGC** | 0.99 (0.04) | 0.37 (0.03) | 0.96 (0.08) | 0.38 (0.03) |
| **SDCN** | 0.92 (0.26) | 0.40 (0.15) | 0.84 (0.24) | 0.49 (0.19) |
| **ODTGCNet** | 0.55 (0.01) | 0.56 (0.01) | 0.55 (0.01) | 0.55 (0.01) |

## **Supplementary Table S14**

Supplementary Table S14. Comparison of clustering performance of risk stratification models at 4 hours after sepsis diagnosis.

| **Time steps** | **Models** | **MIMIC-III** | | **eICU** | |
| --- | --- | --- | --- | --- | --- |
|  |  | **DBI** | **SC** | **DBI** | **SC** |
| **0-4** | **DEC** | 1.08 (0.13) | 0.33 (0.06) | 1.09 (0.19) | 0.34 (0.11) |
|  | **IDEC** | 0.98 (0.13) | 0.41 (0.09) | 0.96 (0.14) | 0.43 (0.09) |
|  | **DAEGC** | 1.00 (0.01) | 0.36 (0.01) | 1.01 (0.08) | 0.38 (0.04) |
|  | **SDCN** | 0.96 (0.10) | 0.34 (0.04) | 0.90 (0.12) | 0.42 (0.11) |
|  | **ODTGCNet** | 0.64 (0.01) | 0.48 (0.01) | 0.61 (0.01) | 0.50 (0.01) |
| **0-16** | **DEC** | 1.08 (0.14) | 0.35 (0.06) | 1.11 (0.15) | 0.33 (0.09) |
|  | **IDEC** | 1.04 (0.18) | 0.39 (0.09) | 0.98 (0.12) | 0.42 (0.09) |
|  | **DAEGC** | 1.00 (0.02) | 0.36 (0.01) | 1.02 (0.05) | 0.35 (0.02) |
|  | **SDCN** | 0.89 (0.14) | 0.37 (0.05) | 0.86 (0.27) | 0.49 (0.21) |
|  | **ODTGCNet** | 0.55 (0.02) | 0.56 (0.02) | 0.53 (0.01) | 0.57 (0.01) |
| **0-32** | **DEC** | 1.09 (0.12) | 0.34 (0.06) | 1.14 (0.14) | 0.32 (0.07) |
|  | **IDEC** | 1.07 (0.16) | 0.37 (0.08) | 1.00 (0.11) | 0.40 (0.08) |
|  | **DAEGC** | 0.98 (0.03) | 0.37 (0.03) | 1.02 (0.13) | 0.37 (0.05) |
|  | **SDCN** | 0.91 (0.28) | 0.42 (0.16) | 0.87 (0.24) | 0.46 (0.18) |
|  | **ODTGCNet** | 0.55 (0.01) | 0.56 (0.02) | 0.54 (0.01) | 0.56 (0.01) |

## **Supplementary Table S15**

Supplementary Table S15. Performance comparison of model structure ablation.

| **Models** | **MIMIC-III** | | **eICU** | |
| --- | --- | --- | --- | --- |
|  | **DBI** | **SC** | **DBI** | **SC** |
| **ODTGCNet_VAE_** | 0.66 (0.03) | 0.49 (0.02) | 0.66 (0.02) | 0.48 (0.01) |
| **ODTGCNet_AE_** | 0.71 (0.05) | 0.46 (0.03) | 0.67 (0.03) | 0.47 (0.02) |
| **ODTGCNet_Att-_** | 1.03 (0.07) | 0.32 (0.02) | 0.90 (0.17) | 0.38 (0.07) |
| **ODTGCNet_GCN_** | 0.69 (0.06) | 0.49 (0.03) | 0.77 (0.08) | 0.44 (0.03) |
| **ODTGCNet** | 0.55 (0.01) | 0.56 (0.01) | 0.55 (0.01) | 0.55 (0.01) |

## **Supplementary Table S16**

Supplementary Table S16. Performance comparison of loss functions ablation

| **Models** | **MIMIC-III** | | **eICU** | |
| --- | --- | --- | --- | --- |
|  | **DBI** | **SC** | **DBI** | **SC** |
| **ODTGCNet**$\mathcal{L}_{\boldsymbol{S}}$**-** | 0.88 (0.15) | 0.38 (0.05) | 1.07 (0.07) | 0.31 (0.02) |
| **ODTGCNet**$\mathcal{L}_{\boldsymbol{AR}}$**-** | 0.55 (0.02) | 0.55 (0.01) | 0.59 (0.04) | 0.53 (0.03) |
| **ODTGCNet**$\mathcal{L}_{\boldsymbol{C}}$**-** | 0.57 (0.01) | 0.55 (0.01) | 0.59 (0.02) | 0.52 (0.01) |
| **ODTGCNet** | 0.55 (0.01) | 0.56 (0.01) | 0.55 (0.01) | 0.55 (0.01) |

## **Supplementary Table S17**

Supplementary Table S18. Clustering performance of different data processing methods (mean(std)).

| **Data processing** | **DBI** | **SC** |
| --- | --- | --- |
| ODTGCNet | 0.55 (0.01) | 0.56 (0.01) |
| MICE | 0.55 (0.02) | 0.55 (0.02) |
| Winsorization | 0.56 (0.01) | 0.54 (0.01) |

## **Supplementary Table S18**

Supplementary Table S18. Fluid category item IDs from MIMIC-III.

| **Tables** | **Item IDs** |
| --- | --- |
| inputevents_mv  and  inputevents_cv | 225158,225943,226089,225168,225828,225823,220862,220970,220864,225159,220995,225170,225825,227533,225161,227531,225171,225827,225941,225823,225825,225941,225825,228341,225827,30018,30021,30015,30296,30020,30066,30001,30030,30060,30005,30321,3000630061,30009,30179,30190,30143,30160,30008,30168,30186,30211,30353,30159,30007,30185,30063,30094,30352,30014,30011,30210,46493,45399,46516,40850,30176,30161,30381,30315,42742,30180,46087,41491,30004,42698,42244 |

# Supplementary Reference

1. Johnson AEW, Pollard TJ, Shen L, et al. MIMIC-III, a freely accessible critical care database. *Scientific Data* 2016; **3**(1): 160035.

2. Pollard TJ, Johnson AEW, Raffa JD, Celi LA, Mark RG, Badawi O. The eICU Collaborative Research Database, a freely available multi-center database for critical care research. *Scientific Data* 2018; **5**(1): 180178.

3. Shi C, Wei B, Wei S, Wang W, Liu H, Liu J. A quantitative discriminant method of elbow point for the optimal number of clusters in clustering algorithm. *EURASIP Journal on Wireless Communications and Networking* 2021; **2021**(1): 31

4. Shahapure KR, Nicholas C. Cluster quality analysis using silhouette score. Proceedings of the 7th IEEE International Conference on Data Science and Advanced Analytics (DSAA); 2020: IEEE; 2020. p. 747-8.

5. Petrovic S. A comparison between the silhouette index and the davies-bouldin index in labelling ids clusters. Proceedings of the 11th Nordic workshop of secure IT systems; 2006: Citeseer; 2006. p. 53-64.

6. Xie J, Girshick R, Farhadi A. Unsupervised deep embedding for clustering analysis. Proceedings of the International Conference on Machine Learning; 2016 Jun 20-22; New York, NY; 2016. p. 478-87.

7. Guo X, Gao L, Liu X, Yin J. Improved deep embedded clustering with local structure preservation. Proceedings of the 26th International Joint Conference on Artificial Intelligence (IJCAI); 2017 Aug 19-25; Melbourne, AUSTRALIA; 2017. p. 1753-9.

8. Wang C, Pan S, Hu R, Long G, Jiang J, Zhang C. Attributed graph clustering: A deep attentional embedding approach. Proceedings of the 28th International Joint Conference on Artificial Intelligence; 2019 Aug 10-16; Macao, PEOPLES R CHINA; 2019. p. 3670-6.

9. Bo D, Wang X, Shi C, et al. Structural deep clustering network. Proceedings of the 29th World Wide Web Conference (WWW); 2020 Apr 20-24; Taipei, TAIWAN; 2020. p. 1400-10.
